# Therapeutic Effect and Mechanism Study of *Rhodiola wallichiana* var. *cholaensis* Injection to Acute Blood Stasis Using Metabolomics Based on UPLC-Q/TOF-MS

**DOI:** 10.1155/2019/1514845

**Published:** 2019-11-03

**Authors:** Nan Ran, Zhiqiang Pang, Xuewa Guan, Guoqiang Wang, Jinping Liu, Pingya Li, Jingtong Zheng, Fang Wang

**Affiliations:** ^1^Department of Pathogen Biology, College of Basic Medical Sciences, Jilin University, Changchun 130021, China; ^2^Research Center of Natural Drug, School of Pharmaceutical Sciences, Jilin University, Changchun 130021, China

## Abstract

In traditional Chinese medicine theory, blood stasis syndrome (BSS), characterized by blood flow retardation and blood stagnation, is one of the main pathologic mechanisms and clinical syndromes of cardiovascular diseases (CVDs). *Rhodiola wallichiana* var. *cholaensis* injection (RWCI) is made from dry roots and stems of RWC via the processes of decoction, alcohol precipitation, filtration, and dilution. Studies indicated the extracts of RWC could alleviate CVDs; however, the mechanism had not been illustrated. In the present study, the acute blood stasis rat model was established to investigate the pathogenesis of BSS and the therapeutic mechanism of RWCI against BSS. Hemorheological parameters (whole blood viscosity and plasma viscosity) and inflammatory factors (TNF-*α* and IL-6) were used to evaluate the success of the BSS rat model and RWCI efficacy. 14 and 33 differential metabolites were identified from plasma and urine samples using the metabolomics approach based on ultrahigh-performance liquid chromatography coupled with quadrupole time-of-flight mass spectrometry. The results of multivariate analysis displayed that there were significant separations among model, control, and treatment groups, but the high-dose RWCI treatment group was closer to the control group. 9 perturbed metabolic pathways were related to BSS's development and RWCI intervention. 5 metabolic pathways (arachidonic acid metabolism, linoleic acid metabolism, alpha-linolenic acid metabolism, retinol metabolism, and steroid hormone biosynthesis) showed apparent correlations. These differential metabolites and perturbed metabolic pathways might provide a novel view to understand the pathogenesis of BSS and the pharmacological mechanism of RWCI.

## 1. Introduction

In recent years, cardiovascular diseases (CVDs) have become a leading cause of death worldwide [1]. In order to control the increasing morbidity and mortality of CVDs, it is necessary to develop novel and effective therapeutic strategies. Integrating the multichannel, multicomponent, and multitarget characteristics, traditional Chinese medicine (TCM) based on cardiovascular drugs has been widely used to prevent and treat CVDs [2, 3]. In TCM theory, blood stasis syndrome (BSS) is one of the main pathologic mechanisms and clinical syndromes of CVDs [4]. BSS is featured by blood flow retardation and blood stagnation and caused by several factors, for example, Qi deficiency, Qi stagnation, phlegm turbidity, cold coagulation, and blood insufficiency [5, 6]. As one of the common TCM symptoms, BSS is always accompanied by the change of clinical manifestations and signs, hemorheological disorder, and vascular endothelial cell (VEC) damage and inflammation [7, 8]. Furthermore, BSS may result in multiple organ dysfunction [9]. Previous studies have reported that TCM might significantly improve the BSS by activating circulation to remove blood stasis [10]. However, the pathophysiological mechanisms have not been elucidated.

*Rhodiola*, growing in cool and moist areas, has various species including *Rhodiola wallichiana* var. *cholaensis* (RWC), *Rhodiola rosea*, *Rhodiola crenulata*, and *Rhodiola sachalinensis* [11]. Currently, *Rhodiola* has been applied to improve coronary heart disease, bronchial asthma, and chronic fatigue syndrome in clinical practice [12]. RWC has broad therapeutic effects on human diseases, such as neuroprotective, antidepressive, antitumour, and cardioprotective activities, with milder effects and less frequent adverse effects [13]. The chemical constituents of RWC injection (RWCI) have been comprehensively studied. On the one hand, a total of 49 compounds including 10 organic acids, 9 phenylethanoids, 10 phenylpropanoids, 2 flavonoid glycosides, 7 monoterpene glycosides, 7 octylglycosides, and 4 other types of compounds were identified using the ultrahigh-performance liquid chromatography coupled with quadrupole time-of-flight mass spectrometry (UPLC-Q/TOF-MS) analysis method [14]. On the other hand, the fingerprint RWCI had been established by HPLC [15]. Because of the diversity of components and biological activities, RWC has various functions in medicine and health, for instance, immune modulation, enhanced immunity [16], antitumor activity [17], and strengthening the ability of antianoxia [18]. RWCI is made from dry roots and stems of RWC via the processes of decoction, alcohol precipitation, filtration, and dilution. Recently, a study revealed that the RWC capsule had a significant effect on angina pectoris, one type of coronary heart disease [19]. RWCI is used to promote blood circulation and clear blood stasis, while the precise pharmacological mechanisms of the prevention and treatment of BSS are rarely reported.

Metabolomics, an emerging omics research approach, is employed to analyse the change of low-molecular-weight metabolites (<1 kDa) in the complex biosystems influenced by endogenous and exogenous factors [20]. As a branch of system biology, metabolomics adopts a holistic research strategy to illustrate the metabolic process of the organism [21]. Unlike the genome and proteome, metabolome, a bridge between the genome and the phenotype, is viewed as the endpoint of biological processes [22]. The comprehensive thinking of metabolomics is consistent with the viewpoint of “whole,” “dynamic process,” and “dialectics” in TCM theory. Therefore, metabolomics is widely used to investigate the pharmacological effect of TCM, identify the compounds of Chinese herbs, and discover potential biomarkers related to diseases [23]. Especially, the UPLC-Q/TOF-MS method was developed as a very effective biochemical analytical tool for precise identification of important biomarkers, especially for large-scale untargeted metabolic profiling in complex biological samples because of the high resolution of chromatographic peaks, high throughput, and high sensitivity for complex mixtures [24–26]. In conclusion, metabolomics is a beneficial and worthwhile tool to explore the potential therapeutic mechanism of TCM and the pathogenesis of diseases.

To further explore the pathogenesis of BSS and the therapeutic mechanisms of RWCI against BSS, in the present study, the acute blood stasis rat model was established by ice-water bath combined with adrenaline. The success of the model and RWCI therapeutic effect were evaluated by hemorheological parameters (whole blood viscosity and plasma viscosity) and inflammatory factors. Subsequently, we analysed the change of metabolic profiling in plasma and urine samples from experimental rats using metabolomics based on UPLC-Q/TOF-MS. Potential distinct metabolites were used for further metabolic pathway analysis. Differential metabolites and perturbed metabolic pathways have been hypothesized to illustrate the pathogenesis of BSS and the molecular mechanism of RWCI against BSS.

## 2. Materials and Methods

### 2.1. Experimental Reagents

RWCI (Lot No. X1001170405) was provided by Tonghua Yusheng Pharmaceutical Co., Ltd. (Jilin, China; Z20060361). Adrenaline hydrochloride injections (Lot No. 1611281) were manufactured by Suicheng Pharmaceutical Co., Ltd. (Henan, China; H41021054). Compound danshen tablets (CDSTs) (Lot No. C17A050) were obtained from Hutchison Whampoa Guangzhou Baiyun Shan Chinese Medicine Co., Ltd. (Guangzhou, China; Z44023372). HPLC-grade acetonitrile (Lot No. 175165) was purchased from Fisher Chemical Company (Shanghai, China). Heparin sodium (Lot No. 1304049) and pentobarbital sodium (Lot No. 3289010) were produced by Sigma-Aldrich (St. Louis, MO, USA). Purified deionized water from the Milli-Q water purification system (Millipore, Billerica, MA, USA) was used for the preparation of aqueous solutions. All-*trans*-retinoic acid (Lot No. 57F-3895), 9-*cis*-retinoic acid (Lot No. 107F-1633), 12(s)-HPETE (Lot No. 58F-1750), and 5-HPETE (Lot No. 241F-1496) were purchased from Sigma-Aldrich (St. Louis, MO, USA). The ELISA kits of tumor necrosis factor alpha (TNF-*α*) (Lot No. A28280745) and interleukin-6 (IL-6) (Lot No. A20680741) were purchased from Multisciences (Lianke) Biotech, CO., Ltd. (Guangzhou, China).

### 2.2. Experimental Rats and Animal Model

Forty-eight healthy female SD rats (200 ± 20 g) were purchased from Yi-Si Animal Co., Ltd. (Jilin, China; Certificate No. SCXK (JI)-2016-0003). Those animals were raised in a comfortable environment with a temperature and humidity of 25 ± 2°C and 50 ± 10%. Moreover, 12-hour circulation of light and dark was supplied. Enough chow and water were provided for rats during the experiment. The whole experiment followed Nutrition Guidelines for the Care and Use of Laboratory Animals and was approved by the Institutional Animal Care and Use Committee of Jilin University (No. SCXK-2013-0001, September 24, 2017).

The animals were randomly and equally divided into six groups including the model group (M), control group (C), high-dose (5 ml/kg/day, TH), middle-dose (2.5 ml/kg/day, TM), and low-dose (1.25 ml/kg/day, TL) RWCI treatment groups, and CDST group (0.3 g/kg/day) (positive control group) after adapting to the environment for seven days. The corresponding administration dose was calculated by referring to *Pharmacopoeia of the People's Republic of China* (2015 edition, Volume I) [27] and *Experimental Methodology of Pharmacology* compiled by Wei et al. [28]. The BSS rat model was established according to previous researches [29, 30]. In brief, the treated animals were administered by gavage with corresponding doses of RWCI and CDSTs for eight days, respectively. The animals in model and control groups were given normal saline. Except those in the control group, rats in other groups were placed in ice-cold water for 5 min daily for eight consecutive days and made to keep their heads above the water surface. At the eighth day, rats, except those in the control group, were subcutaneously injected adrenaline twice at a dose of 0.8 mg/kg in four-hour intervals. Moreover, ice-cold bath was performed after two hours of the first injection. Rats in the control group received an equal volume of saline by the way of subcutaneous injection.

### 2.3. Sample Collection and Processing

After 30 min of the last injection of epinephrine, the rats in treatment groups were administered by gavage with corresponding doses of RWCI and CDSTs. Urine samples from every rat were collected for twelve hours by putting rats in metabolic cages and were then immediately stored at −80°C until analysis. In this period, urine samples were stored at 4°C. Then, all rats were intraperitoneally anesthetized with 3% pentobarbital sodium (1.5 mL/kg) after the collection of urine samples. Blood samples, from the abdominal aorta, were collected and mixed with 1% heparin sodium dilution (10 mg/mL) in 5 mL vacuum blood-collection tubes with heparin sodium anticoagulation for the hemorheological test and metabolomics analysis, respectively, after the collection of urine samples. Plasma was separated from blood by centrifuging at 3000 rpm for 10 min at 4°C. 1 mL was used for the plasma viscosity test. 200 *μ*L was immediately stored at −80°C for metabolomics analysis.

### 2.4. Hemorheology and Inflammatory Factor Detection

Whole blood viscosity (at high, middle, and low shear rates of 120s^−1^, 60s^−1^, and 10s^−1^) and plasma viscosity (at the shear rate of 120s^−1^) were detected by using an LBY-N6 blood viscometer (Precil, Beijing, China) at 37°C. These experiments were completed within 2 hours after blood sample collection. The levels of cytokines (TNF-*α* and IL-6) in plasma samples were detected using the corresponding ELISA kits as recommended by the product's instructions.

### 2.5. Sample Preparation for Metabolomics Analysis

Both plasma and urine samples were thawed on ice before metabolomics analysis. Plasma samples were centrifuged at 3,000 rpm for 10 min at 4°C. The mixture of plasma (200 *μ*L) and acetonitrile (600 *μ*L) was vortexed for 3 min. Next, they were settled at room temperature for 10 min and then centrifuged at 12,000 rpm for 10 min at 4°C. After the precooling at −80°C, 500 *μ*L supernatant was lyophilized at −60°C and 10.0 pa air pressure for 18 hours. The residue was redissolved in 100 *μ*L 80% acetonitrile and then centrifuged at 12,000 g for 15 min at 4°C. An aliquot of 3 *μ*L was injected for metabolomics analysis. Urine samples were centrifuged at 12,000 rpm for 10 min at 4°C. Moreover, the supernatant (1 mL) was separated for metabolomics analysis.

### 2.6. UPLC-Q/TOF-MS Conditions

The UPLC-Q/TOF-MS-based metabolomics analysis of plasma and urine samples was conducted by the Waters ACQUITY UPLC system (Waters Corporation, Milford, MA, USA) equipped with the BEH C18 column (2.1  mm × 100  mm, 1.7 mm; Waters Corporation, Milford, MA, USA), coupled with the Waters Xevo G2-S Q/TOF mass spectrometer (Waters Corporation, Milford, MA, USA) with an electrospray ionization in both positive (ESI^+^) and negative (ESI^−^) ion modes. Column temperature was set as 30°C, sample manager temperature as 15°C, sample volume as 5 μL, and flow rate of mobile phases in the UPLC system as 0.4 mL/min. The mobile phases consisted of eluent A (0.1% formic acid in water, v/v) and eluent B (0.1% formic acid in acetonitrile, v/v). The gradient procedure was optimized as follows: 10% B from 0 to 2 min, 10–90% B from 2 to 26 min, 90% B from 26 to 28 min, 90–10% B from 28 to 28.1 min, and 10% B from 28.1 to 30 min. The parameters of chromatography and mass spectrometry were based on the previous study of our research team [31]. In brief, the capillary voltages and sample cone voltage were 2.6 kV and 40 V in the ESI^+^ mode, respectively. However, they were 2.2 kV and 40 V in the ESI^−^ mode. The cone gas flow rate was 50 L/h under the 120°C source temperature condition. The desolvation gas rate flow was 800 L/h at 300°C desolvation temperature. Low and high collision energies were set as 6.0 V and 20–40 V, respectively, in the MSE mode of mass spectrometry. Leucine-enkephalin (concentration: 300 ng/mL; flow rate: 20 *μ*L/min) was regarded as the lock-mass solution.

Quality control (QC) samples of plasma and urine were prepared by mixing the equal volume (20 *μ*L) of all preprocessed plasma and urine samples, respectively, which were used to ensure the stability and repeatability of UPLC-Q/TOF-MS analysis. QC samples were continuously tested six times in ESI^+^ and ESI^−^ modes, respectively, before the test of plasma and urine samples, which were used to evaluate the system method repeatability. The QCs were closely clustered in both positive and negative ion modes in both urinary and plasma samples, indicating the system method was stable. The intermediate precision and repeatability of the systematic method were validated by selecting ten chromatographic peaks of ions with high abundances in ESI^+^ and ESI^−^ modes, respectively, from the QC sequencing datasheet, covering the whole analysis process. Then, QC samples were tested again prior to the test of every group in plasma and urine samples to ensure the stability and suitability of MS analysis.

### 2.7. Data Processing

The raw data from metabolomics analysis were processed by the MarkerLynx XS v4.1 software (Waters, Milford, CT, USA) for alignment, deconvolution, data reduction, normalization, etc., which exported datasheet about the pair of mass and retention time with the corresponding intensities of all detected peaks. The main parameters were based on previous research. Retention time was set as 0 to 30 min, mass as 100 Da to 1,200 Da, mass tolerance as 0.10, minimum intensity as 5%, marker intensity threshold as 2,000, mass window as 0.10 Da, retention time window as 0.20 min, and noise elimination level as 6 [31].

The data in the datasheet were performed for multivariate analysis (principle component analysis (PCA) and orthogonal projections to latent structures discriminant analysis (OPLS-DA)) using the MarkerLynx XS v4.1 software and SIMCA-P software (v14.1; Umetrics, Umeå, Sweden). OPLS-DA, producing S-plots and permutation plots, contributed to finding significantly distinct metabolites in the metabolic process. The quality parameters (R2X, R2Y, and Q2) were employed to access the ability of the main components of variables to build models and samples, fitness between the model and samples, and prediction ability of the systematic model. Variable importance of project (VIP) values were also estimated statistically to indicate a significant difference between the groups. Metabolites were chosen based on VIP values (VIP > 1) from the OPLS-DA model and *P* values (*P* < 0.05) from the *t*-test for further identification of distinct metabolites and relative metabolic pathway analysis. The distinct metabolites were identified by matching accurate molecular weight and characteristic tandem mass spectrometry (MS/MS) fragmentation to the Human Metabolome Database (HMDB, Version 4.0) [31]. If three or more than three MS/MS fragments of a metabolite were matched to spectrum view in the HMDB, we thought that this metabolite was a potential distinct metabolite. All metabolites included in statistical analyses were confirmed by the HMDB with the parameters set as follows: adducts were *M* + H˥^+^ and *M* + Na˥^+^ for ESI^+^ and M-H˥^−^ and *M* + FA-H˥^−^ for ESI^−^. The tolerance of mass was set as 10 ppm. Some metabolites were further demonstrated by referring to the chemical standards. The identification and comparison of some metabolites against the chemical standard samples were performed according to the retention time, the accurate molecular weight, and the characteristic MS/MS fragments. Then, the receiver-operating characteristic (ROC) curve was used to estimate the accuracy of identified metabolites as potential biomarkers. The differential metabolites with area under the curve (AUC) >0.9 were considered potential biomarkers because of high sensitivity and specialty. Metabolic pathway analysis based on the identified distinct metabolites was performed by MetaboAnalyst 4.0 [33], which could get important metabolic pathways by setting the impact-value threshold above 0.10. The metabolic network analysis was performed with the Cytoscape software (v3.6.1) based on the data from the Kyoto Encyclopedia of Genes and Genomes (KEGG; updated on January 1, 2019) database [34]. The heatmap of all distinct metabolites identified in the plasma and urine samples came from R's ComplexHeatmap (v1.19.1) package.

### 2.8. Statistical Analysis

Hemorheological data were expressed as mean values ± standard deviation (SD). Multiple comparisons among groups were performed by one-way analysis of variance (ANOVA) by using the GraphPad 6.0 software (GraphPad Software Inc., San Diego, CA, USA). Data normality was evaluated by the Kolmogorov–Smirnov test. Statistical homogeneity of variance was estimated by using the *F*-test. Comparison between two groups was performed by the *t*-test. In the case of homogeneity of variance, *P* value was calculated using Student's *t* test; otherwise, *P* value was calculated by Welch's *t*-test. Nonnormal data were analysed by the Wilcoxon rank-sum test. All the statistical analyses about the identification of distinct metabolites were performed with R (v3.3.3). *P* < 0.05 indicated differences had statistical significance.

## 3. Results

### 3.1. Effects of RWCI on Hemorheology and Inflammation in Rats with BSS

Compared with healthy rats, BSS rats gradually moved slowly in action. The colors of lips, claws, and eyes were dark red in BSS model rats, which were significantly improved after RWCI and CDST treatment. In addition, the bodies of BSS rats were longer than those of healthy rats. Compared with the control group, both whole blood viscosity and plasma viscosity were significantly increased in the model group (*P* < 0.01) (see Table 1), which indicated the BSS model was successfully established and abnormal blood flow had appeared in BSS rats. However, these results were significantly decreased in RWCI and CDST treatment groups compared with the model group (*P* < 0.05), which showed RWCI had a potential to inhibit and improve the hemorheological disorder, thereby preventing the occurrence of BSS.

**Figure 1:**
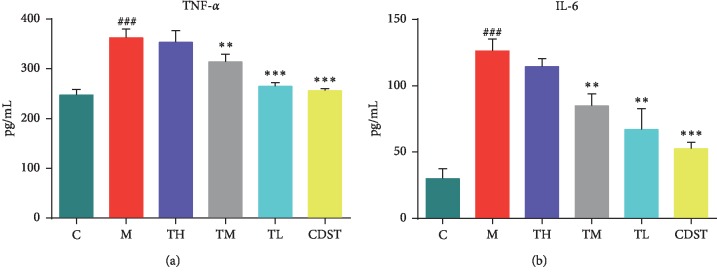
Effects of RWCI on the levels of inflammatory factors (TNF-*α* and IL-6) in BSS rats. C: control group; M: model group; TH: high-dose RWCI treatment group; TM: middle-dose RWCI treatment group; TL: low-dose RWCI treatment group; CDST: compound danshen tablet group. Compared with the control group, ###, ##, and # represent *P* < 0.001, *P* < 0.01, and *P* < 0.05, respectively. Compared with the model group, ^*∗∗∗*^, ^*∗∗*^, and ^*∗*^ represent *P* < 0.001, *P* < 0.01, and *P* < 0.05, respectively.

In addition to hemorheological parameters, the effects of inflammatory factors, including TNF-*α* and IL-6, on the BSS rats were also evaluated in the present study (see Figure 1). Compared to the control group, the levels of TNF-*α* and IL-6 were significantly increased (*P* < 0.001) in BSS model rats. The abnormal levels of cytokines were markedly improved in high-dose and middle-dose RWCI and CDST treatment groups (*P* < 0.05). However, the low dose of RWCI showed no significant effect with only the decreased trend in rats with BSS. The results indicated that BSS might also have a great relationship with inflammation.

### 3.2. Multivariate Analysis Based on Metabolomics Data of Plasma and Urine Samples

**Figure 2:**
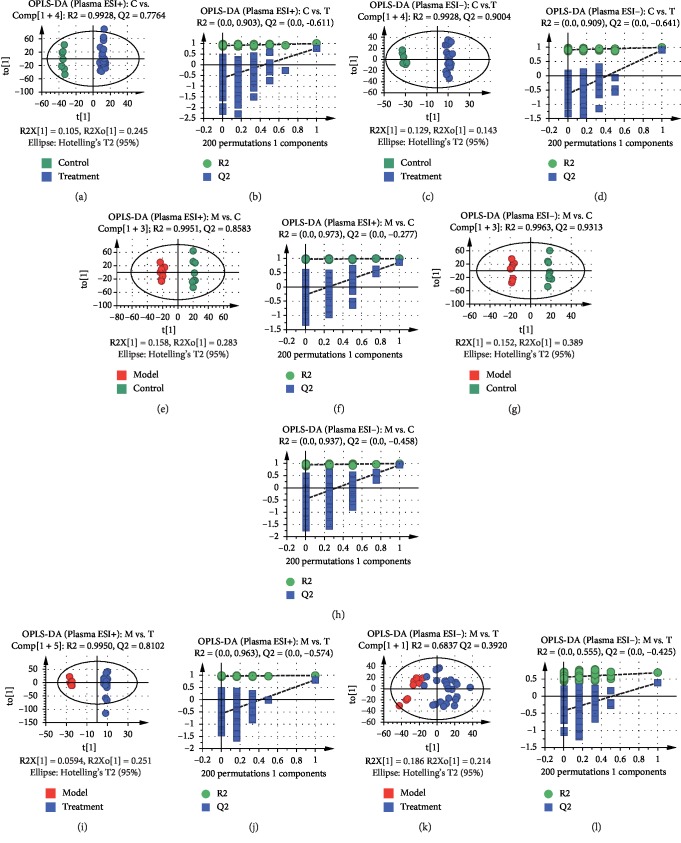
OPLS-DA score plots and permutation test results of metabolomics analysis based on plasma samples: results between the control group and the RWCI treatment groups in the ESI^+^ mode (a, b) and in the ESI^−^ mode (c, d); results between the model group and the control group in the ESI^+^ mode (e, f) and in the ESI^−^ mode (g, h); results between the model group and the RWCI treatment groups in the ESI^+^ mode (i, j) and in the ESI^−^ mode (k, l). All *P* values were less than 0.01, calculated by the CV-ANOVA test.

**Figure 3:**
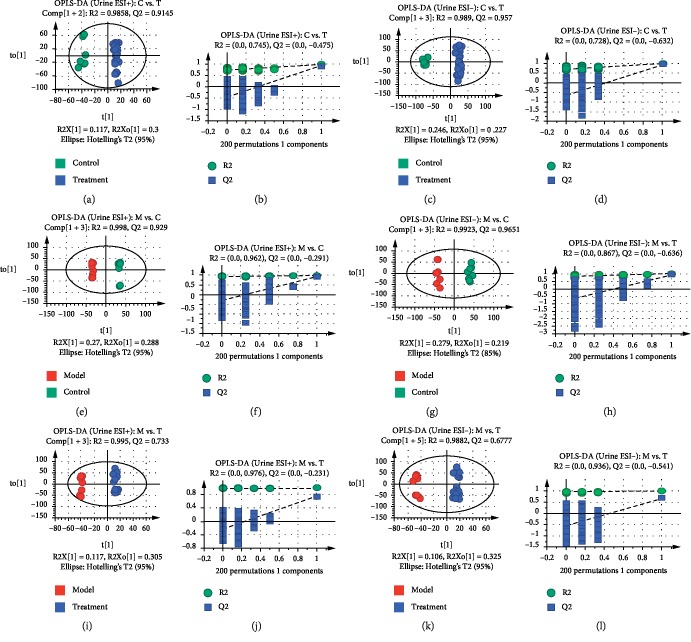
OPLS-DA score plots and permutation test results of metabolomics analysis based on urine samples: results between the control group and the RWCI treatment groups in the ESI^+^ mode (a, b) and in the ESI^−^ mode (c, d); results between the model group and the control group in the ESI^+^ mode (e, f) and in the ESI^−^ mode (g, h); results between the model group and the RWCI treatment groups in the ESI^+^ mode (i, j) and in the ESI^−^ mode (k, l). All *P* values were less than 0.01, calculated by the CV-ANOVA test.

The validation of the systematic method was evaluated by calculating the relative standard deviation (RSD) of retention times and *m/z* for repeatability and intermediate precision in ESI^+^ and ESI^−^ modes of plasma and urine samples, respectively. All RSD values were less than 3.3%, indicating this method could be used for subsequent analysis (see [Supplementary-material supplementary-material-1] and [Supplementary-material supplementary-material-1]) [35]. The PCA plots of plasma and urine samples in ESI^+^ and ESI^−^ modes showed significant separations in the model group and other groups. The model was reliable because Q2 was over 0.4 except in PCA of the plasma sample in the ESI^−^ mode [36]. Moreover, high-dose RWCI group and CDST group were closer to the control group than middle-dose and low-dose RWCI groups (see [Supplementary-material supplementary-material-1]), manifesting rats in RWCI and CDST treatment groups had similar metabolic patterns with healthy rats. Moreover, the RWCI curative effect displayed dosage dependence. OPLS-DA, a supervised clustering model, was performed to find discriminatory metabolic markers associated with BSS. The plasma and urine samples between the control group and the RWCI treatment groups, between the model group and the control group, and between the model group and the RWCI treatment groups had significant separations in OPLS-DA plots in ESI^+^ and ESI^−^ modes. Permutation tests (*n* = 200) were used to validate the OPLS-DA model (see Figures 2 and 3). All R2 and Q2 values from tests were less than original data, and the intersection of blue regression lines of Q2 points and Y-axis was less than zero, which showed the validity and reliability of the model for further analysis (see [Supplementary-material supplementary-material-1]).

### 3.3. Identification of Differential Metabolites in Plasma and Urine Samples

**Figure 4:**
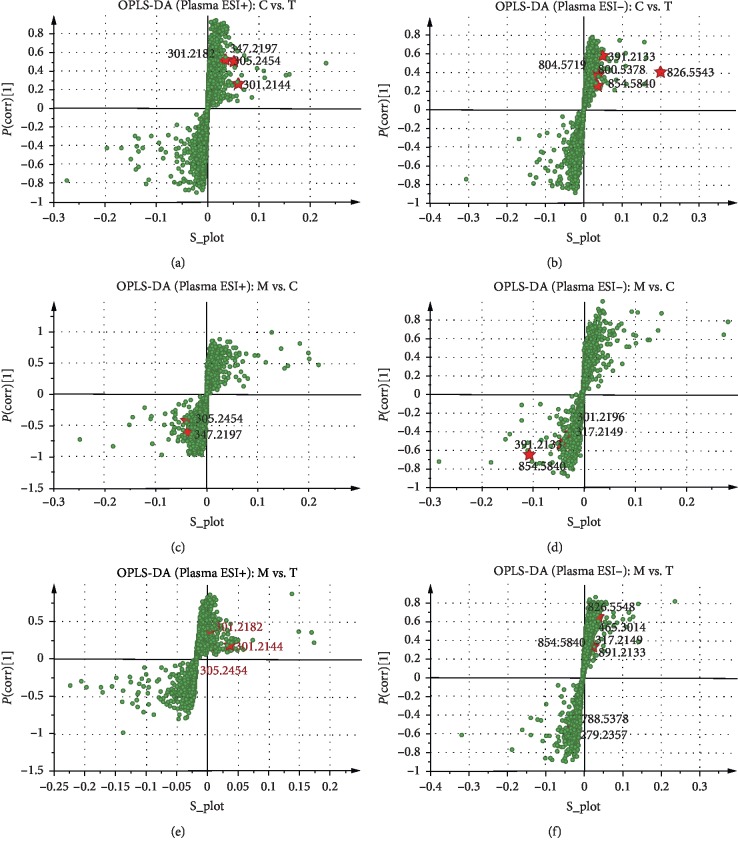
OPLS-DA S-plots of metabolomics analysis based on plasma samples. All differential metabolites are shown in S-plots, including the identified results between the control group and the RWCI treatment groups in the ESI^+^ mode (a) and in the ESI^−^ mode (b), the identified results between the model group and the control group in the ESI^+^ mode (c) and in the ESI^−^ mode (d), and the identified results between the model group and the RWCI treatment groups in the ESI^+^ mode (e) and in the ESI^−^ mode (f). C: control group; M: model group; T: RWCI treatment groups. The *p*(corr)[1]-axis shows the reliability of each variable in *X*.

**Figure 5:**
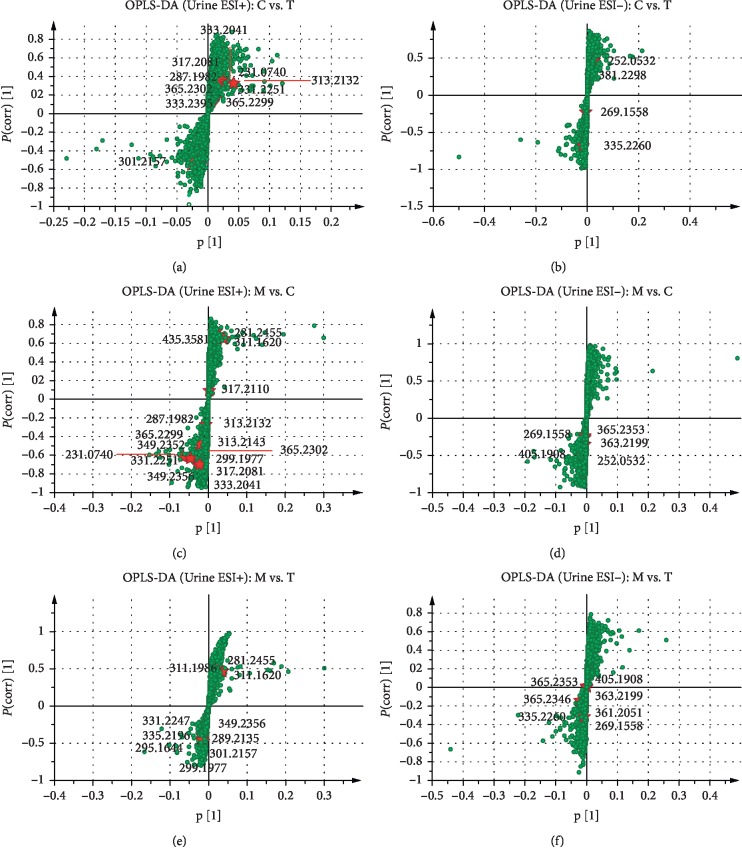
OPLS-DA S-plots of metabolomics analysis based on urine samples. All differential metabolites are shown in S-plots, including the identified results between the control group and the RWCI treatment groups in the ESI^+^ mode (a) and in the ESI^−^ mode (b), the identified results between the model group and the control group in the ESI^+^ mode (c) and in the ESI^−^ mode (d), and the identified results between the model group and the RWCI treatment groups in the ESI^+^ mode (e) and in the ESI^−^ mode (f). C: control group; M: model group; T: RWCI treatment groups. The *p*(corr)[1]-axis shows the reliability of each variable in *X*.

Fourteen and 33 differential metabolites significantly contributed to the occurrence of BSS found in plasma and urine samples, respectively (see Tables 2 and 3). The MS/MS spectrum of discriminatory metabolites is shown in Figures [Supplementary-material supplementary-material-1]–[Supplementary-material supplementary-material-1] (plasma samples) and Figures [Supplementary-material supplementary-material-1]–[Supplementary-material supplementary-material-1] (urine samples). Moreover, linoleic acid, alpha-linolenic acid, all-*trans*-retinoic acid, 9-*cis*-retinoic acid, arachidonic acid, and leukotriene a4 were identified in both plasma and urine samples. Potential distinct metabolites identified in plasma and urine samples were marked in OPLS-DA S-plots in the form of exact mass (see Figures 4 and 5). The ROC curve was used to estimate the accuracy of identified metabolites as potential biomarkers. In plasma samples, expect cortexolone and PC (14:0/20:3), other 12 differential metabolites with AUC > 0.9 were considered potential biomarkers because of high sensitivity and specialty (see [Supplementary-material supplementary-material-1]). Similarly, in urine samples, 26 differential metabolites with AUC > 0.9 were considered potential biomarkers (see [Supplementary-material supplementary-material-1]).

In plasma and urine samples, retinoic acids including all-*trans*-retinoic acid and 9-*cis*-retinoic acid were identified by matching the retention time, the accurate molecular weight, and the characteristic MS/MS fragments to chemical standard spectra (see Figures [Supplementary-material supplementary-material-1], [Supplementary-material supplementary-material-1], [Supplementary-material supplementary-material-1], and [Supplementary-material supplementary-material-1]). In urine samples, HPETEs including 12(s)-HPETE and 5-HPETE also were identified by comparing with chemical standard spectra (see Figures [Supplementary-material supplementary-material-1] and [Supplementary-material supplementary-material-1]).

**Figure 6:**
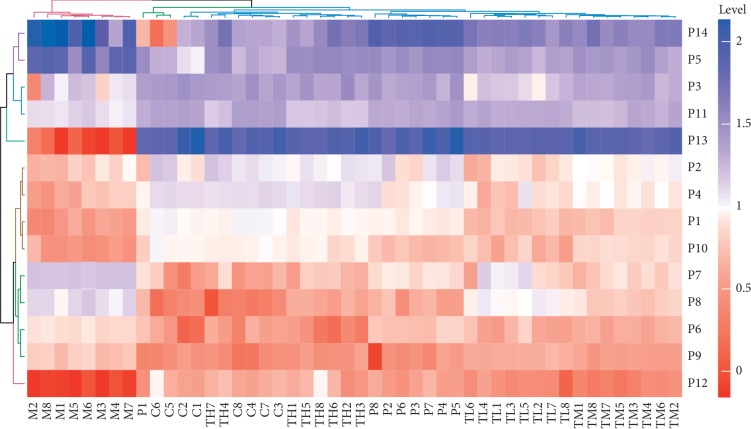
Heatmap of differential metabolites identified from plasma samples.

**Figure 7:**
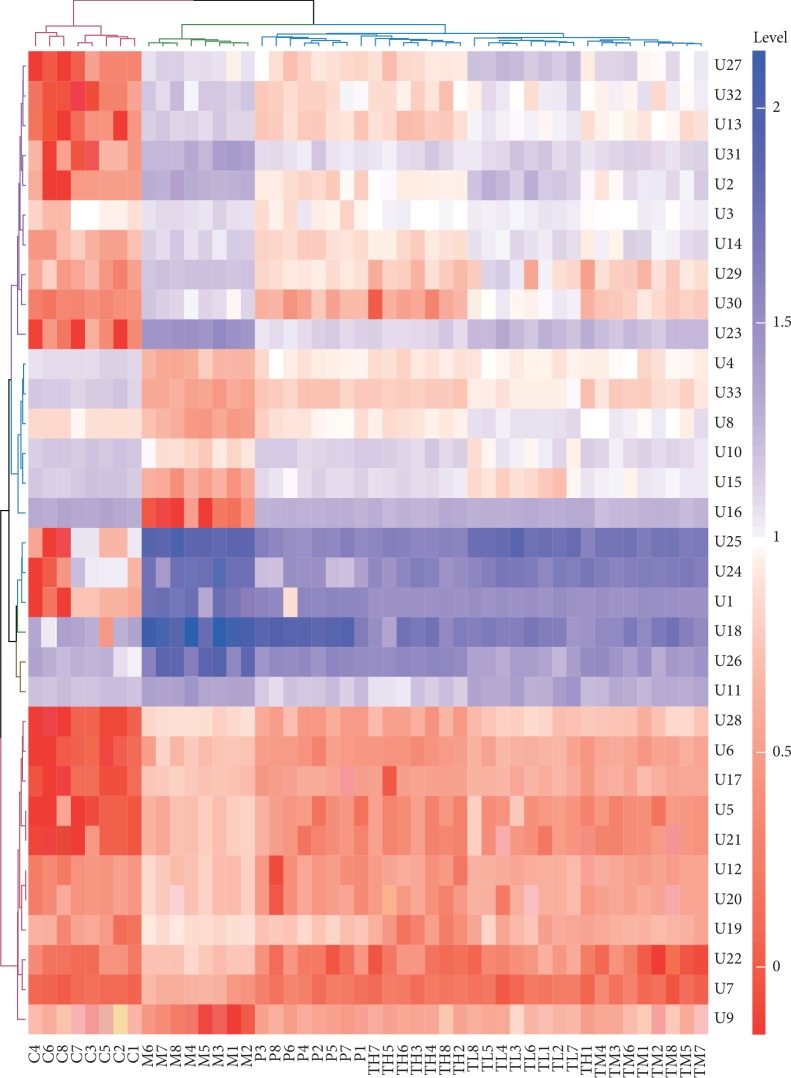
Heatmap of differential metabolites identified from urine samples.

The relative levels of differential metabolites identified from plasma and urine samples are shown in Table 4. The levels of P1-4 and P11-13 were decreased in plasma samples of BSS rats; however, the levels of P5-10 and P14 were increased. Similarly, the levels of U4, U8-10, U15, U16, and U33 were decreased in urine samples of BSS rats; however, the levels of U1-3, U5-7, U11-14, and U17-32 were increased. The post hoc analysis for multiple comparisons was performed for pairwise comparison between the model group and the control group and between the model group and the treatment groups (see [Supplementary-material supplementary-material-1]). The levels of distinct metabolites had a very significant difference between the model group and the control group (*P* < 0.01), between the model group and the high-dose RWCI treatment group (*P* < 0.01), and between the model group and the CDST treatment group (*P* < 0.01). All distinct metabolites levels were significantly different between the model group and the middle-dose and low-dose RWCI treatment groups (*P* < 0.05). In contrast, there was no significant difference between the control group and the high-dose RWCI treatment group (*P* > 0.05). RWCI had a potential to alleviate the disordered levels of these metabolites. In addition, the potential distinct metabolites were visually presented using heatmaps. In the heatmap of plasma samples, all samples were clustered as two groups, which indicated the levels of metabolites had a small difference in the control group and treatment groups (see Figure 6). However, all samples were clustered as three groups in the heatmap of urine samples, including the control group, model group, and treatment groups (see Figure 7).

### 3.4. Relative Metabolic Pathway Analysis

**Figure 8:**
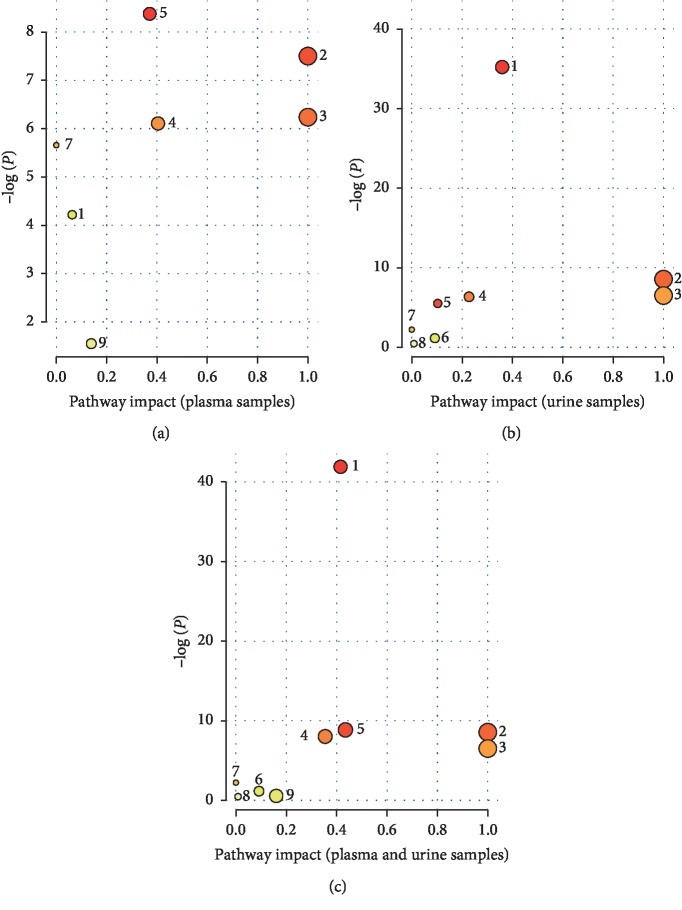
Metabolic pathway analysis performed by MetaboAnalyst 4.0. Perturbed metabolic pathways were identified in plasma samples (a), urine samples (b), and both plasma and urine samples (c). The numbers represent different metabolic pathways in (a), (b), and (c). 1: steroid hormone biosynthesis; 2: linoleic acid metabolism; 3: alpha-linolenic acid metabolism; 4: retinol metabolism; 5: arachidonic acid metabolism; 6: tryptophan metabolism; 7: biosynthesis of unsaturated fatty acids; 8: steroid biosynthesis; 9: glycerophospholipid metabolism. The values of pathway impact represent the significance of metabolic pathways.

The disturbed metabolic pathways associated with the occurrence of BSS were analysed in the present study. 7 perturbed metabolic pathways were identified in plasma samples, including steroid hormone biosynthesis, linoleic acid metabolism, alpha-linolenic acid metabolism, retinol metabolism, arachidonic acid metabolism, biosynthesis of unsaturated fatty acids, and glycerophospholipid metabolism (see Figure 8(a)). 8 perturbed metabolic pathways were investigated in urine samples, including steroid hormone biosynthesis, linoleic acid metabolism, alpha-linolenic acid metabolism, retinol metabolism, arachidonic acid metabolism, tryptophan metabolism, biosynthesis of unsaturated fatty acids, and steroid biosynthesis (see Figure 8(b)). Therefore, 9 perturbed metabolic pathways (steroid hormone biosynthesis, linoleic acid metabolism, alpha-linolenic acid metabolism, retinol metabolism, arachidonic acid metabolism, tryptophan metabolism, biosynthesis of unsaturated fatty acids, steroid biosynthesis, and glycerophospholipid metabolism) were potentially related with the pathogenesis of BSS's development and the pharmacological mechanism of RWCI intervention (see Figure 8(c)). In Figure 8, the pathway impact value represents the significance of the metabolic pathway. The −log (*P*) value represents the importance of the metabolic pathway enrichment analysis. The size of the circle is positively correlated with the values of pathway impact and −log (*P*). The detailed information of disordered metabolic pathways is presented in [Supplementary-material supplementary-material-1]. In this study, the pathway impact value and *P* value were set to 0.10 and 0.01 according to previous researches [31, 37, 38]. 5 of the metabolic pathways (steroid hormone biosynthesis, linoleic acid metabolism, alpha-linolenic acid metabolism, retinol metabolism, and arachidonic acid metabolism) showed evident changes in BSS rats according to impact >0.1 and *P* < 0.01, which indicated they might be significantly associated with BSS's development and RWCI intervention. The glycerophospholipid metabolism pathway had a potential relativity because impact >0.1 but *P* > 0.05.

**Figure 9:**
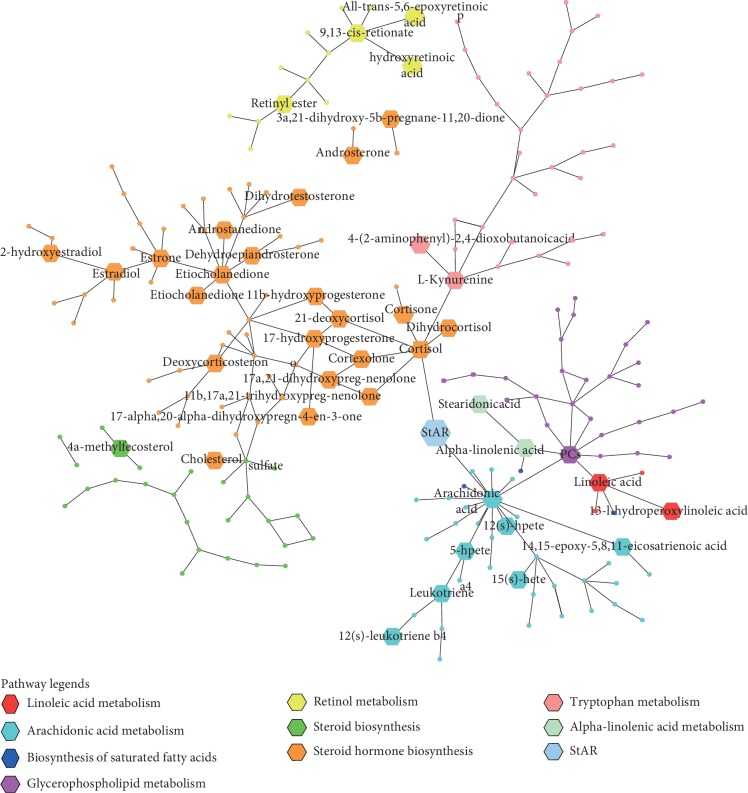
Metabolic network of all differential metabolites identified in plasma and urine samples. StAR: steroidogenic acute regulatory protein.

A metabolic network of all differential metabolites identified in plasma and urine samples is displayed in Figure 9. 9 perturbed metabolic pathways are presented in different colors. Retinol metabolism was not involved in the formation of the whole network.

## 4. Discussion

In recent years, the UPLC/MS technique has been widely used to explore the pathological mechanism of diseases and the pharmacological activity of medicines by identifying the distinct metabolites. For example, UPLC/MS-based metabolomics methods were used to identify the differential metabolites in the rat model of postmenopausal osteoporosis, thereby illustrating its pathogenesis and the therapeutic effects of *Acanthopanax senticosus* [39]. Furthermore, a UPLC/MS-based metabolomics study found that the antiulcer activity of *C. alternifolius* tubers might be related to *α*-carbonic anhydrase inhibitory, anti-inflammatory, and analgesic activity by their antioxidant activity and downregulation of several inflammatory mediators [40]. We also explored the inhibitory effect of methotrexate on rheumatoid arthritis inflammation using UPLC-Q/TOF-MS [41]. In addition to investigating pharmacological activity, the UPLC/MS technique was also used in metabolite identification, which might provide new insights for the development and progression of diseases. The metabolite biomarkers, such as aristolochic acid, kynurenic acid, and hippuric acid, might be associated with nephrotoxicity [42–45]. UPLC/MS was also used to analyse and discover reliable biomarkers for the diagnosis and prognosis of hepatitis B-related acute-on-chronic liver failure (ACLF) [46]. Researchers found that 17 metabolites were related to prognosis of hepatitis B-related ACLF.

In the present study, UPLC-Q/TOF-MS-based metabolomics was utilized to identify distinct metabolites associated with the occurrence of BSS and the disturbed metabolic pathways in BSS rats, thereby uncovering its pathogenesis and the therapeutic effect of RWCI against BSS. Hemorheology was employed to explore the flow properties of blood in the vessel [47]. In the current study, whole blood viscosity and plasma viscosity, the main hemorheological parameters, were higher in BSS rats than in healthy control rats. Increased blood viscosity was related to the decrease of blood flow speed and tissue hypoxia, which might further lead to metabolic disorders. However, whole blood viscosity and plasma viscosity were significantly improved in BSS rats after RWCI treatment, indicating RWCI might have a potential role in activating blood circulation and eliminating BSS. In addition, other studies showed that thrombin time, prothrombin time, and activated partial thromboplastin time were significantly shortened and fibrinogen content was significantly increased in BSS rats, which indicated that the blood of rats with BSS is in a state of being easily coagulated [5, 48]. The coagulation dysfunction is a vital risk factor for thrombosis and cardiovascular diseases [49]. The levels of TNF-*α* and IL-6 were significantly increased in BSS model rats compared to healthy rats. Moreover, high dose and middle dose of RWCI were effective in the improvement of inflammation in BSS rats. Plasma and urine samples were commonly used for metabolomics analysis. 14 and 33 differential metabolites were identified in plasma and urine samples from BSS rats using metabolomics based on UPLC-Q/TOF-MS, involving 9 disturbed metabolic pathways. 5 metabolic pathways were significantly related to BSS's development and RWCI intervention. The levels of these differential metabolites in BSS rats could be improved after RWCI treatment.

Decreased blood flow velocity and increased blood viscosity were main clinical signs of BSS in ancient TCM theory and modern researches [50]. The primary risk factors leading to the occurrence of BSS were anger and sorrow of the seven emotions in TCM theory, and exogenous stimulating factors like cold [51]. Based on this theory, an acute blood stasis rat model was built in the current study. Increased whole blood viscosity and plasma viscosity showed the success of acute blood stasis rat model establishment. CSMTs have been widely applied in clinical vascular diseases by promoting blood flow and resolving blood stasis in China and other Asian countries [52]; therefore, they were chosen as the positive control drug in the present study.

Lipid metabolism, such as steroid hormone biosynthesis, linoleic acid metabolism, alpha-linolenic acid metabolism, arachidonic acid metabolism, biosynthesis of unsaturated fatty acids, and glycerophospholipid metabolism, was perturbed in BSS rats in this study, which was consistent with previous researches [5, 53, 54]. Phosphatidylcholines (PCs) were main phospholipids in the cell membrane structure, involving various biological processes like signal transduction [55] and anti-inflammatory effect [56]. 5 homologues of PCs were identified in plasma samples in the present study, displaying different changing trends. Results showed increased levels of PC (15:0/18:2) and PC (20:4/18:0) as well as the reduced concentrations of PC (14:0/20:3), PC (14:0/20:1), and PC (16:0/20:4) in BSS rats, indicating the metabolic disorder of PCs might be associated with the occurrence and progression of BSS. PCs could be resolved into lysophosphatidylcholine (LysoPC) and unsaturated fatty acids including linoleic acid, alpha-linolenic acid, and arachidonic acid by phospholipase A2 [5]. LysoPC damaged the normal function of vascular endothelial cells and induced thrombogenesis by decreasing the mRNA levels of nitric oxide synthetase and inhibiting the transcriptional activity of nuclear factor kappa-light-chain-enhancer of activated B cells (NF-κB) and tissue factors.

The pathogenesis of blood stasis was thought to be related to inflammatory factors and immune response in recent studies [57, 58]. Arachidonic acid and its metabolites (eicosanoids) could promote the production of proinflammatory cytokines and inflammatory response [59]. The activation of phospholipase A2 by the protein kinase C pathway would cause the release of arachidonic acid in the blood stasis state, thereby being resolved into various proinflammatory eicosanoids, like leukotrienes (LTs), prostaglandins (PGs), and thromboxanes by lipoxygenase and cyclooxygenase [60], which might then promote the secretion of inflammatory factors like tumor necrosis factor-*α* (TNF-*α*) and interleukin-1 (IL-1) [37]. Leukotriene a4 (LTA4) hydrolase can catalyze LTA4 into LTB4, an important proinflammatory lipid mediator, which was involved in a series of pathological and physiological processes like the chemotaxis, aggregation, and activation of polymorphonuclear leucocytes and monocytes [61]. In the current study, the levels of arachidonic acid, LTA4, 12(s)-LTB4, 15(s)-HETE, 14,15-epoxy-5,8,11-eicosatrienoic acid, 12(s)-HPETE, and 5-HPETE were found to be higher in BSS model rats than in healthy rats. However, RWCI could improve the fluctuant levels of arachidonic acid and eicosanoids in BSS rats. Therefore, we speculated that the initiation of BSS might be associated with inflammation caused by the disorder of arachidonic acid metabolism. Moreover, RWCI could help prevent these disorders and inhibit the inflammatory state of BSS.

Linoleic acid, a shortest-chain polyunsaturated fatty acid, was the precursor of arachidonic acid. As an essential amino acid, alpha-linolenic acid was employed to produce docosahexaenoic acid and eicosapentaenoic acid (EPA), the latter of which could suppress inflammation by targeting IL-6 and TNF-*α* [62]. Stearidonic acid was a metabolic intermediate in the conversion process of alpha-linolenic acid and EPA. Concentrations of linoleic acid, alpha-linolenic acid, and stearidonic acid were decreased in rats with BSS compared with healthy rats. However, the disturbance of these levels could be ameliorated after RWCI treatment. In previous research, metabolic disorders of linoleic acid and alpha-linolenic acid were also found in BSS and were then improved by TCM, for example, the treatment of the acute blood stasis model rat with Taohong Siwu decoction [63].

Steroid hormone biosynthesis mainly included the production of androgens and estrogens. Steroidogenic acute regulatory protein (StAR) could mediate cholesterol into mitochondria to synthesize pregnenolone [64], which was regulated by arachidonic acid- and cyclic adenosine monophosphate-transduced signals [65]. Cortisol had an important effect on regulating the stress response of the organism [66]. A study indicated that hydrocortisone could amplify the responses of the leg vasoconstrictor to the cold pressor test and vascular smooth muscle to catecholamines [67]. Dehydroepiandrosterone sulfate, converted from dehydroepiandrosterone, could inhibit inflammation in atherosclerosis by activating peroxisome proliferator-activated receptors and regulating the NF-κB signal pathway [68]. In addition, the disorder of steroid hormone biosynthesis was related to primary dysmenorrhea accompanied by BSS [69]. Steroid hormones levels were increased in acute blood stasis model rats compared with healthy rats and were improved with RWCI treatment, indicating that inflammation and endocrine disorder might participate in the development of BSS. These results were in accord with those of previous researches [54, 70].

Retinoic acids were involved in various cell activities in the biological process, for example, the secretion of matrix metalloproteinase, cell communication and differentiation, and the expression of inflammatory factors like IL-6 and TNF-*α* [71]. Previous studies have shown that the decreased levels of retinoic acid in asthma subjects might lead to airway hyperresponsiveness and accelerate disease progression by activating NF-κB [72, 73]. In the present study, the levels of compounds participated in retinol metabolism, such as all-*trans*-retinoic acid, 9-*cis*-retinoic acid, retinyl ester, and hydroxyretinoic acid, were decreased in BSS rats. Moreover, their levels were increased after RWCI therapy. These results indicated RWCI might improve retinol metabolism disorder, thereby relieving the syndrome of blood stasis.

Kynurenine, as a main catabolite of tryptophan, participated in the regulation of innate and adaptive immunity in biological processes. It might inhibit the proliferation of immune cells such as T cells and natural killer cells. In addition, it could negatively modulate immunogenic dendritic cells [74]. Tryptophan metabolism had a potential correlation with blood stasis in the current research. The increased level of kynurenine in BSS rats could be improved by RWCI treatment.

## 5. Conclusions

In the current study, metabolomics was utilized to explore the pathogenesis of BSS and pharmacological effect of RWCI. The change of clinical manifestations and signs, hemorheological parameter, and inflammation in BSS rats could be markedly improved after RWCI treatment. 14 and 33 differential metabolites in plasma and urine samples were identified by metabolomics based on UPLC-Q/TOF-MS, mainly involving 9 disturbed metabolic pathways. Thereinto, 5 metabolic pathways (steroid hormone biosynthesis, linoleic acid metabolism, alpha-linolenic acid metabolism, retinol metabolism, and arachidonic acid metabolism) were significantly related to the occurrence of BSS. The pathogenesis of BSS might be associated with inflammatory responses and endocrine disorder. However, RWCI had a potential to prevent the disorder of metabolic pathways, indicating RWCI might be considered a novel TCM to ameliorate BSS. These results might provide corresponding experimental basis for clinical application. Subsequent researches should further explore the changes of genes and proteins associated with disturbed metabolic pathways, thereby uncovering relevant signal pathways related to the occurrence of BSS.

## Figures and Tables

**Table 1:** Effects of RWCI on whole blood viscosity and plasma viscosity in rats with BSS.

Group	Whole blood viscosity (mPa·s)			Plasma viscosity (mPa·s)
	10s^−1^	60s^−1^	120s^−1^	120s^−1^
C	11.51 ± 0.97^*∗∗*^	7.14 ± 0.74^*∗∗*^	5.95 ± 0.78^*∗∗*^	1.06 ± 0.32^*∗∗*^
M	15.38 ± 1.22	9.02 ± 0.68	7.39 ± 0.72	1.42 ± 0.17
TH	12.03 ± 1.11^*∗∗*^	7.63 ± 0.88^*∗∗*^	6.66 ± 0.69^*∗*^	1.19 ± 0.6^*∗∗*^
TM	12.77 ± 1.21^*∗∗*^	7.72 ± 0.65^*∗∗*^	6.69 ± 0.67^*∗*^	1.26 ± 0.54^*∗∗*^
TL	13.25 ± 0.82^*∗∗*^	8.15 ± 0.76^*∗∗*^	7.01 ± 0.63^*∗*^	1.39 ± 0.37^*∗*^
CDST	11.84 ± 1.34^*∗∗*^	7.43 ± 0.79^*∗∗*^	6.54 ± 0.63^*∗∗*^	1.13 ± 0.60^*∗∗*^

^*∗*^ *P* < 0.05 and ^*∗∗*^*P* < 0.01 vs. model group. C: control group; M: model group; TH: high-dose RWCI treatment group; TM: middle-dose RWCI treatment group; TL: low-dose RWCI treatment group; CDST: compound danshen tablet group.

**Table 2:** Differential metabolites identified in plasma samples.

No.	Compound name	Formula	MS fragmentation^E^	*t*_R_ (min)	Mass (Da)	KEGG ID	VIP	ESI	Error (ppm)	Pathway
P1	Linoleic acid	C_18_H_32_O_2_	91.0550, 119.0844, 121.099	23.98	279.2357	C01595	1.13	−	10	Linoleic acid
P2	Alpha-linolenic acid	C_18_H_30_O_2_	91.0550, 119.0844, 121.0991	21.6	279.2301	C06427	1.42	+	2	Alpha-linolenic acid
P3	Retinoic acids	C_20_H_28_O_2_	123.0932, 149.1257, 301.2041	14.34	301.2144	C00777	3.86	+	6	Retinol
P4	Retinyl ester	C_20_H_30_O_2_	134.8988, 146.9695, 283.2684	21.42	301.2196	C02075	1.14	−	8	Retinol
P5	Arachidonic acid	C_20_H_32_O_2_	106.9040, 162.8759, 190.8695	22.9	305.2454	C00219	3.63	+	7	Arachidonic acid
P6	Leukotriene a4	C_20_H_30_O_3_	243.9020, 245.8972, 317.2140	15.37	317.2149	C00909	1.35	−	8	Arachidonic acid
P7	Cortexolone	C_21_H_30_O_4_	121.0644, 123.0799, 171.1152	9.76	347.2197	C05488	2.45	+	6	Steroid hormone biosynthesis
P8	21-Deoxycortisol	C_21_H_30_O_4_	134.8972, 243.8986, 245.8962	9.81	391.2133	C05497	2.27	−	2	Steroid hormone biosynthesis
P9	Cholesterol sulfate	C_27_H_46_O_4_S	112.9903, 327.2350, 383.1917	27.57	465.3014	C18043	1.13	−	6	Steroid hormone biosynthesis
P10	PC (15:0/18:2)	C_41_H_78_NO_8_P	122.9904, 279.2365, 393.2784	25.8	788.5378	C00157	1.04	−	9	Arachidonic acid, alpha-linolenic acid, linoleic acid, and glycerophospholipid
P11	PC (14:0/20:3)	C_42_H_78_NO_8_P	183.0163, 259.2466, 303.2379	27.99	800.5378	C00157	1.75	−	9	
P12	PC (14:0/20:1)	C_42_H_82_NO_8_P	112.9912, 183.0157, 305.2471	27.1	804.5719	C00157	1.17	−	5	
P13	PC (16:0/20:4)	C_44_H_80_NO_8_P	239.0619, 255.2331, 303.2323	28.1	826.5543	C00157	1.71	−	7	
P14	PC (20:4/18:0)	C_46_H_84_NO_8_P	255.2368, 279.2371, 303.2366	25.4	854.584	C00157	1.15	−	9	

Retinoic acids include all-*trans*-retinoic acid (C00777) and 9-*cis*-retinoic acid (C15493). *t*_R_: retention time; ^E^tandem mass spectrum ion mass fragments in MS/MS: three major fragment ions with high abundances of each distinct metabolite are listed; ESI^+^: Q/TOF mass spectrometer with an electrospray ionization in the positive mode; ESI^−^: Q/TOF mass spectrometer with an electrospray ionization in the negative mode; PC: phosphatidylcholine. PCs are involved in arachidonic acid metabolism, alpha-linolenic acid metabolism, linoleic acid metabolism, and glycerophospholipid metabolism.

**Table 3:** Differential metabolites identified in urine samples.

No.	Compound name	Formula	MS fragmentation^E^	*t*_R_ (min)	Mass (Da)	KEGG ID	VIP	ESI	Error (ppm)	Pathway
U1	L-Kynurenine	C_10_H_12_N_2_O_3_	146.0583, 147.1145, 165.0665	6.95	231.074	C00328	1.78	+	0	Tryptophan metabolism
U2	4-(2-Aminophenyl)-2,4-dioxobutanoic acid	C_10_H_9_NO_4_	144.0485, 160.0436, 165.0595	0.67	252.0532	C01252	1.9	−	7	Tryptophan metabolism
U3	Estrone	C_18_H_22_O	295.0858, 171.1138, 271.1667	7.84	269.1558	C00468	1.31	−	4	Steroid hormone biosynthesis
U4	Linoleic acid	C_18_H_32_O	281.0719, 97.1016, 121.1001	24.15	281.2455	C01595	1.54	+	7	Linoleic acid metabolism
U5	Androstanedione	C_19_H_28_O	296.9614, 109.1013, 149.0218	8.99	287.1982	C00674	1.77	+	8	Steroid hormone biosynthesis
U6	Dehydroepiandrosterone	C_19_H_28_O	296.9611, 145.0980, 173.1301	13.35	289.2135	C01227	1.21	+	9	Steroid hormone biosynthesis
U7	Estradiol	C_18_H_24_O_2_	119.0852, 145.0992, 243.1364	8.15	295.1644	C00951	1.19	+	8	Steroid hormone biosynthesis
U8	Stearidonic acid	C_18_H_28_O_2_	133.0992, 149.0206, 159.1133	8.01	299.1977	C16300	1.09	+	1	Alpha-linolenic acid metabolism
U9	Alpha-linolenic acid	C_18_H_30_O_2_	109.1004, 119.0844, 121.0991	10.41	301.2138	C06427	1.01	+	0	Alpha-linolenic acid metabolism
U10	Retinoic acids	C_20_H_28_O_2_	123.0932, 149.1257, 301.2041	17.13	301.2157	C00777	1.2	+	2	Retinol metabolism
U11	2-Hydroxyestradiol	C_18_H_24_O_3_	135.0426, 149.0214, 161.0941	22.04	311.162	C05301	2.83	+	1	Steroid hormone biosynthesis
U12	Etiocholanedione	C_19_H_28_O_2_	96.9611, 109.1007, 175.1449	7.44	311.1986	C03772	1.77	+	1	Steroid hormone biosynthesis
U13	Dihydrotestosterone	C_19_H_30_O_2_	109.1005, 215.0669, 227.1756	8.99	313.2132	C03917	1.71	+	2	Steroid hormone biosynthesis
U14	Androsterone	C_19_H_30_O_2_	107.0849, 145.0990, 147.1144	10.03	313.2143	C00523	1.52	+	2	Steroid hormone biosynthesis
U15	Hydroxyretinoic acid	C_20_H_28_O_3_	105.0706, 189.0532, 215.1055	8.4	317.2081	C16677	1.33	+	0	Retinol metabolism
U16	All-*trans*-5,6-epoxyretinoic acid	C_20_H_28_O	3105.0706, 189.0532, 215.1055	11.1	317.211	C16680	1.36	+	0	Retinol metabolism
U17	Deoxycorticosterone	C_21_H_30_O_3_	217.0565, 289.2139, 313.2133	6.03	331.2247	C03205	1.24	+	6	Steroid hormone biosynthesis
U18	17-Hydroxyprogesterone	C_21_H_30_O_3_	109.1005, 271.0565, 289.2139	11.55	331.2251	C01176	3.56	+	5	Steroid hormone biosynthesis
U19	11b-Hydroxyprogesterone	C_20_H_28_O_4_	269.1883, 287.1989, 333.2040	9.79	333.204	C05498	1.83	+	6	Steroid hormone biosynthesis
U20	17-Alpha,20-alpha dihydroxypregn-4-en-3-one	C_21_H_32_O_3_	143.0840, 177.1606, 261.1457	7.66	333.2395	C04518	1.05	+	9	Steroid hormone biosynthesis
U21	13-l-Hydroperoxylinoleic acid	C_18_H_32_O_4_	156.8872, 163.0721, 267.0013	13.5	335.2196	C04717	1.5	+	1	Linoleic acid metabolism
U22	12(s)-Leukotriene b4	C_20_H_32_O_4_	113.0288, 141.8739, 247.0743	10.41	335.226	C04853	1.26	−	10	Arachidonic acid metabolism
U23	3a,21-Dihydroxy-5b-pregnane-11,20-dione	C_21_H_32_O_4_	97.0710, 313.2144, 349.2357	11.51	349.2352	C05478	2.16	+	6	Steroid hormone biosynthesis
U24	17a,21-Dihydroxypregnenolone	C_21_H_32_O_4_	157.0990, 313.2144, 349.2357	6.19	349.2356	C05487	5.12	+	5	Steroid hormone biosynthesis
U25	Cortisol	C_21_H_30_O_5_	329.1620, 331.1892, 361.2032	8.65	361.2015	C00735	1.21	−	2	Steroid hormone biosynthesis
U26	Leukotriene a4	C_20_H_30_O_3_	243.1349, 245.0459, 255.0635	9.89	363.2199	C00909	1.77	−	6	Arachidonic acid metabolism
U27	11b,17a,21-Trihydroxypregnenolone	C_21_H_32_O_5_	109.0996, 157.0980, 242.1904	5.01	365.2299	C05489	1.81	+	6	Steroid hormone biosynthesis
U28	Dihydrocortisol	C_21_H_32_O_5_	157.0980, 329.2062, 365.2264	9.66	365.2302	C05471	1.21	+	6	Steroid hormone biosynthesis
U29	15(s)-HETE	C_20_H_32_O_3_	113.0290, 175.0275, 245.8979	10.53	365.2346	C04742	1.3	−	3	Arachidonic acid metabolism
U30	14,15-Epoxy-5,8,11-eicosatrienoic acid	C_20_H_32_O_3_	113.0293, 219.1411, 229.0205	8.99	365.2353	C14771	3.39	−	5	Arachidonic acid metabolism
U31	HPETEs	C_20_H_32_O_4_	215.1032, 249.0927, 335.2313	3.87	381.2298	C05965	1.79	−	4	Arachidonic acid metabolism
U32	Cortisone	C_21_H_28_O_5_	231.1381, 259.1132, 317.2115	9.43	405.1908	C00762	1.02	−	3	Steroid hormone biosynthesis
U33	4a-Methylfecosterol	C_29_H_48_O	81.0721, 97.1024, 413.2669	25.21	435.3581	C15776	1	+	4	Steroid biosynthesis

Retinoic acids include all-*trans*-retinoic acid (C00777) and 9-*cis*-retinoic acid (C15493). HPETEs include 12(s)-HPETE (C05965) and 5-HPETE (C05356). *t*_R_: retention time; ^E^tandem mass spectrum ion mass fragments in MS/MS: three major fragment ions with high abundances of each distinct metabolite are listed; ESI^+^: Q/TOF mass spectrometer with an electrospray ionization in the positive mode; ESI^−^: Q/TOF mass spectrometer with an electrospray ionization in the negative mode.

**Table 4:** Relative levels of differential metabolites identified in plasma and urine samples.

No.	Levels	*P* values	No.	Levels	*P* values
P1	C > T > P > M	<0.0001	U11	M > T ≈ P ≈ C	0.0390
P2	C > T ≈ P > M	0.0407	U12	M > T ≈ P > C	0.0045
P3	C > T ≈ P > M	0.0093	U13	M > T ≈ P > C	0.0010
P4	C > T > P > M	0.0207	U14	M > T > P > C	0.0300
P5	M > T > P > C	0.0012	U15	C > P ≈ T > M	0.0030
P6	M > P > T > C	<0.0001	U16	C ≈ P ≈ T > M	0.0030
P7	M > T ≈ P > C	0.0010	U17	M > T ≈ P ≈ C	0.0107
P8	M > T > P > C	0.0002	U18	M > T ≈ P > C	0.0156
P9	M > T ≈ P ≈ C	0.0002	U19	M > T ≈ P ≈ C	0.0041
P10	M > T ≈ P > C	0.0122	U20	M > P > T > C	0.0032
P11	C > T ≈ P > M	<0.0001	U21	M > T ≈ P > C	0.0385
P12	C ≈ T ≈ P > M	0.0006	U22	M > T ≈ P ≈ C	0.0097
P13	C ≈ T ≈ P > M	<0.0001	U23	M > T ≈ P > C	0.0095
P14	M > P > T > C	0.0193	U24	M > T > P > C	0.0079
U1	M > P > T > C	0.0047	U25	M > P ≈ T > C	0.0075
U2	M > T > P > C	<0.0001	U26	M > T > P > C	0.0137
U3	M > T ≈ P > C	0.0141	U27	M > P ≈ T > C	0.0044
U4	C > T ≈ P > M	0.0001	U28	M > P ≈ T > C	0.0174
U5	M > T ≈ P > C	0.0139	U29	M > T > P > C	0.0054
U6	M > T ≈ P ≈ C	0.0076	U30	M > P > T > C	0.0244
U7	M > T ≈ P ≈ C	0.0322	U31	M > P > T > C	0.0092
U8	C > P > T > M	0.0409	U32	M > P ≈ T > C	0.0285
U9	C > P ≈ T > M	0.0032	U33	C > T > P > M	0.0007
U10	C > T ≈ P > M	0.0045			

The change fold was calculated by getting the average metabolite intensity in corresponding groups of plasma and urine samples and then calculating the ratios of the natural exponential values between the control group and other groups. Multiple-group comparison was performed by ANOVA, thereby getting *P* values. M: model group; T: RWC treatment groups; P: compound danshen tablet (CDST) group; C: control group.

## Data Availability

The original data employed to support the results of this study can be obtained from the corresponding author upon request.

## Supplementary Materials

Supplementary Materials Tables S1 and S2 are used to validate the systematic stability and repeatability of UPLC-Q/TOF-MS during the test of plasma and urine samples by calculating repeatability and intermediate precision. Figure S1 shows PCA results of plasma and urine samples in ESI^+^ and ESI^−^ modes based on UPLC-Q/TOF-MS. Table S3 shows the details of quality parameters (R2X, R2Y, and Q2) from cross-validation of the OPLS-DA model. The pairwise-comparison *p* values of the post hoc analysis for multiple comparisons in rats are shown in Table S4. Table S5 indicates the relative information of perturbed metabolic pathways in plasma and urine samples. Figures S2 to S16 show mass spectra of all 14 metabolites identified in plasma samples. Among them, retinoic acids include all-*trans*-retinoic acid (Figure S4) and 9-*cis*-retinoic acid (Figure S5). Figures S17 to S51 show mass spectra of all 33 metabolites identified in plasma samples. Among them, retinoic acids include all-*trans*-retinoic acid (Figure S26) and 9-*cis*-retinoic acid (Figure S27). HPETEs include 12(s)-HPETE (Figure S48) and 5-HPETE (Figure S49). The ROC curve is used to evaluate the accuracy of distinct metabolites identified in plasma and urine samples as potential biomarkers (see Figures S52 and S53).
