# Supplementary material for: Therapeutic Effect and Mechanism Study of Rhodiola wallichiana var. cholaensis Injection to Acute Blood Stasis Using Metabolomics Based on UPLC-Q/TOF-MS
Source: Evid Based Complement Alternat Med. 2019 Nov 3;2019:1514845. doi: 10.1155/2019/1514845 (PMC6874959; doi:10.1155/2019/1514845)
Supplement: Supplementary Materials — Tables S1 and S2 are used to validate the systematic stability and repeatability of UPLC-Q/TOF-MS during the test of plasma and urine samples by calculating repeatability and intermediate precision. Figure S1 shows PCA results of plasma and urine samples in ESI+ and ESI− modes based on UPLC-Q/TOF-MS. Table S3 shows the details of quality parameters (R2X, R2Y, and Q2) from cross-validation of the OPLS-DA model. The pairwise-comparison p values of the post hoc analysis for multiple comparisons in rats are shown in Table S4. Table S5 indicates the relative information of perturbed metabolic pathways in plasma and urine samples. Figures S2 to S16 show mass spectra of all 14 metabolites identified in plasma samples. Among them, retinoic acids include all-trans-retinoic acid (Figure S4) and 9-cis-retinoic acid (Figure S5). Figures S17 to S51 show mass spectra of all 33 metabolites identified in plasma samples. Among them, retinoic acids include all-trans-retinoic acid (Figure S26) and 9-cis-retinoic acid (Figure S27). HPETEs include 12(s)-HPETE (Figure S48) and 5-HPETE (Figure S49). The ROC curve is used to evaluate the accuracy of distinct metabolites identified in plasma and urine samples as potential biomarkers (see Figures S52 and S53). [file 1514845.f1.pdf]

Supplementary Materials

The Therapeutic Effect and Mechanism Study of *Rhodiola wallichianavar. Cholaensis* Injection to Acute Blood Stasis Using Metabolomics Based on UPLC-Q/TOF-MS

Nan Ran,[1] Zhiqiang Pang,[1] Xuewa Guan,[1] Guoqiang Wang,[1] Jinping Liu,[2] Pingya Li,[2] Jingtong Zheng,[1]and Fang Wang[1]

1 Department of Pathogen Biology, College of Basic Medical Sciences, Jilin University, Changchun 130021, China.
2 Research Center of Natural Drug, School of Pharmaceutical Sciences, Jilin University, Changchun 130021, China.

Correspondence should be addressed to Fang Wang; wf@jlu.edu.cn

Table S1: Validation of systematic stability and repeatability of UPLC-MS/MS (plasma samples).

Modes	NO	RT/ min	m/z	m/z in Spectrum		RT of Chromatogram	
				Repeatability (RSD%)	Precision (RSD%)	Repeatability (RSD%)	Precision (RSD%)
ESI+	P-1	25.01	284.2935	0.0025	0.0031	0.0010	0.0013
	P-2	27.44	338.3405	0.0026	0.0054	0.0016	0.0021
	P-3	27.14	413.2658	0.0029	0.0041	0.0014	0.0037
	P-4	17.99	496.3424	0.0024	0.0047	0.0000	0.0010
	P-5	20.62	524.3738	0.0019	0.0052	0.0003	0.0007
	P-6	27.12	614.3820	0.0072	0.0016	1.5469	0.3746
	P-7	27.62	786.6028	0.0095	0.0009	0.0012	0.5438
	P-8	27.14	830.5452	0.0013	0.0028	1.3148	0.4056
	P-9	17.99	991.6757	0.0019	0.0007	0.3564	0.4783
	P-10	20.60	1047.7395	0.0034	0.0079	0.5601	0.0013
ESI-	P-1	0.61	215.0349	0.0054	0.0033	0.0021	0.0020
	P-2	24.83	355.2599	0.0051	0.0032	0.0019	0.0052
	P-3	18.03	480.3106	0.0054	0.0025	0.0020	0.0021
	P-4	18.02	540.3315	0.0042	0.0040	0.0019	0.6609
	P-5	20.69	640.2898	0.0039	0.0021	0.9837	0.1702
	P-6	0.53	792.8534	0.0056	0.0031	0.4623	0.0034
	P-7	0.53	860.8389	0.0050	0.0045	0.6138	0.0089

P-8	0.53	928.8245	0.0059	0.0023	1.2341	0.1918
P-9	17.02	1083.6509	0.0059	0.0035	0.1985	0.0495
P-10	17.04	1131.6497	0.0047	0.0030	0.8927	0.2078

m/z, mass to charge ratio, Th; RT, retention time, min, PA, peak area. Precision is intermediate precision, RSD, Relative standard deviation.

Table S2: Validation of systematic stability and repeatability of UPLC-MS/MS (urine samples).

Modes	NO	RT/min	m/z	m/z in Spectrum		RT of Chromatogram	
				Repeatability (RSD%)	Precision (RSD%)	Repeatability (RSD%)	Precision (RSD%)
ESI+	U-1	22.79	282.2778	0.0008	0.0034	1.3827	2.3819
	U-2	27.43	338.3406	0.0013	0.0053	3.2382	0.1414
	U-3	27.15	413.2659	0.0038	0.0055	2.4864	1.4159
	U-4	26.14	540.5339	0.0024	0.0013	0.9178	2.5259
	U-5	28.05	663.4549	0.0000	0.0073	1.2749	0.0084
	U-6	25.83	758.5719	0.0019	0.0062	2.0198	0.9476
	U-7	25.80	834.6038	0.0003	0.0005	0.8134	1.5849
	U-8	27.78	992.7438	0.0046	0.0025	0.1487	2.0185
	U-9	27.77	1075.7151	0.0027	0.0164	0.9847	0.8915
	U-10	27.23	1194.8242	0.0015	0.0528	1.0941	1.3580
ESI-	U-1	6.43	201.0249	0.0024	0.0004	0.0245	0.2429
	U-2	6.31	297.1016	0.0048	0.0028	0.2594	0.1531
	U-3	8.10	357.1038	0.0014	0.0025	0.9147	0.2563
	U-4	5.00	461.1867	0.1141	0.0059	1.2314	1.0313
	U-5	4.32	567.1711	0.0057	0.0024	0.8357	0.9719
	U-6	8.81	659.3276	0.0012	0.0052	0.1479	0.1972
	U-7	6.26	715.2203	0.0013	0.0002	0.1928	0.9816
	U-8	24.79	802.5512	0.0008	0.0015	1.4862	0.8215
	U-9	28.99	885.5432	0.0014	0.0154	0.5839	0.0015
	U-10	12.27	987.5047	0.0094	0.0015	1.4004	0.0948

m/z, mass to charge ratio, Th; RT, retention time, min, PA, peak area. Precision is intermediate precision, RSD, Relative standard deviation.

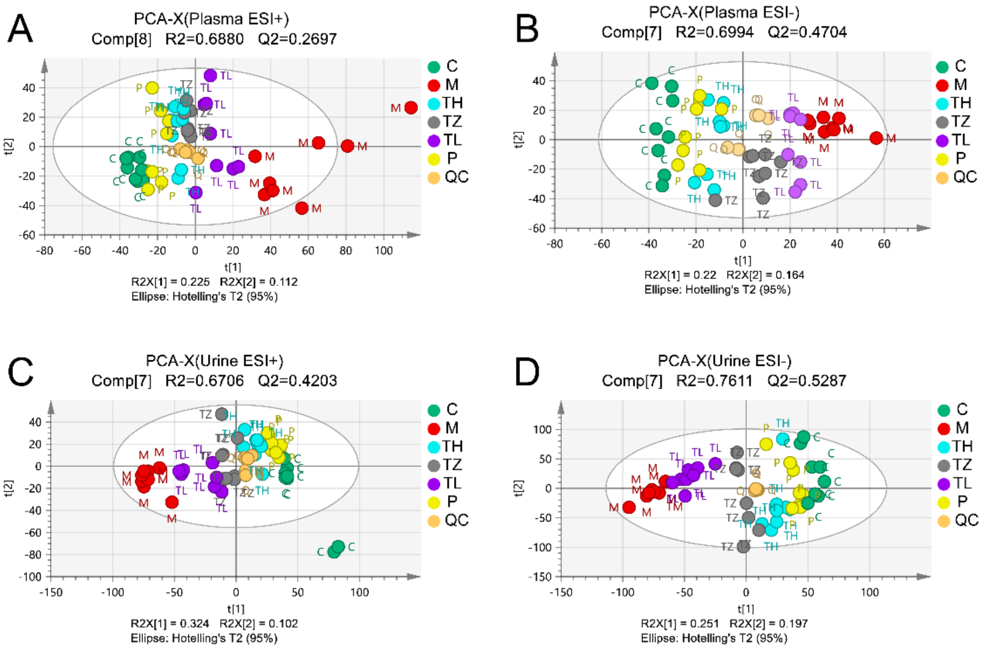

Figure S1: PCA analysis results based on metabolomic data using UPLC -Q/TOF-MS. All plasma samples in ESI^+ modes (A) and in ESI^- modes (B). All urine samples in ESI^+ modes (C) and in ESI^- modes (D).

Table S3: Quality parameters (R2X, R2Y, Q2) from cross-validation of OPLS-DA model.

OPLS-DA Models	R2X	R2Y	Q2	sample
C vs. T in ESI^+ mode	0.541	0.997	0.824	
M vs. C in ESI^+ mode	0.601	0.995	0.859	
M vs. T in ESI^+ mode	0.587	0.995	0.810	plasma
C vs. T in ESI^- mode	0.647	0.993	0.900	
M vs. C in ESI^- mode	0.731	0.996	0.931	
M vs. T in ESI- mode	0.401	0.684	0.392	
C vs. T in ESI^+ mode	0.472	0.986	0.916	
M vs. C in ESI^+ mode	0.774	0.998	0.929	
M vs. T in ESI^+ mode	0.554	0.995	0.733	urine
C vs. T in ESI^- mode	0.669	0.989	0.957	
M vs. C in ESI^- mode	0.781	0.992	0.965	
M vs. T in ESI^- mode	0.699	0.988	0.678	

Table S4: The pairwise-comparison *P*-values of the post-hoc analysis for multiple comparisons in rats.

No.	C vs. M	M vs. TH	M vs. TM	M vs. TL	C vs. TH	M vs. P
P1	0.0005	0.0058	0.0039	0.0149	0.8195	0.0099
P2	0.0089	0.0039	0.0328	0.0001	0.2874	0.0040
P3	0.0046	0.0071	0.0219	0.0148	0.2924	0.0096
P4	0.0091	0.0033	0.0203	0.0417	0.4628	0.0085
P5	0.0032	0.0015	0.0029	0.0009	0.5278	0.0035

P6	0.0003	0.0009	0.0213	0.0028	0.7821	0.0035
P7	0.0061	0.0037	0.0487	0.0009	0.0819	0.0063
P8	0.0007	0.0081	0.0399	0.0149	0.2869	0.0026
P9	0.0007	0.0040	0.0424	0.0099	0.0991	0.0002
P10	0.0087	0.0014	0.0019	0.0209	0.1490	0.0006
P11	0.0009	0.0018	0.0371	0.0091	0.3003	0.0060
P12	0.0027	0.0004	0.0389	0.0049	0.0802	0.0015
P13	0.0003	0.0019	0.0003	0.0248	0.0932	0.0062
P14	0.0067	0.0014	0.0359	0.0249	0.2949	0.0069
U1	0.0048	0.0004	0.0289	0.0147	0.1097	0.0016
U2	0.0008	0.0014	0.0179	0.0049	0.4901	0.0044
U3	0.0068	0.0068	0.0003	0.0032	0.5987	0.0010
U4	0.0018	0.0037	0.0035	0.0007	0.4272	0.0019
U5	0.0059	0.0057	0.0195	0.0184	0.4329	0.0063
U6	0.0064	0.0094	0.0150	0.0147	0.3841	0.0063
U7	0.0081	0.0026	0.0150	0.0429	0.3916	0.0093
U8	0.0019	0.0024	0.0336	0.0310	0.3386	0.0063
U9	0.0015	0.0048	0.0183	0.0049	0.2558	0.0090
U10	0.0037	0.0002	0.0004	0.0001	0.3424	0.0026
U11	0.0027	0.0093	0.0098	0.0040	0.6915	0.0006
U12	0.0041	0.0002	0.0209	0.0140	0.1964	0.0001
U13	0.0063	0.0049	0.0183	0.0091	0.0919	0.0046
U14	0.0052	0.0038	0.0009	0.0004	0.1974	0.0029
U15	0.0063	0.0040	0.0020	0.0319	0.5296	0.0082
U16	0.0009	0.0092	0.0108	0.0294	0.2781	0.0046
U17	0.0062	0.0004	0.0393	0.0067	0.0991	0.0026
U18	0.0005	0.0048	0.0381	0.0037	0.0819	0.0026
U19	0.0062	0.0090	0.0009	0.0139	0.1975	0.0068
U20	0.0093	0.0003	0.0098	0.0421	0.7815	0.0026
U21	0.0024	0.0047	0.0098	0.0098	0.1498	0.0073
U22	0.0004	0.0048	0.0297	0.0018	0.2845	0.0070
U23	0.0073	0.0060	0.0193	0.0407	0.0614	0.0039
U24	0.0085	0.0084	0.0038	0.0298	0.1040	0.0031
U25	0.0073	0.0093	0.0001	0.0480	0.0701	0.0073
U26	0.0065	0.0027	0.0029	0.0367	0.1958	0.0026
U27	0.0070	0.0027	0.0208	0.0058	0.5901	0.0061
U28	0.0058	0.0003	0.0298	0.0004	0.2904	0.0038
U29	0.0063	0.0049	0.0004	0.0046	0.6714	0.0005
U30	0.0004	0.0094	0.0040	0.0498	0.0881	0.0010
U31	0.0073	0.0024	0.0248	0.0249	0.1789	0.0063
U32	0.0045	0.0049	0.0003	0.0050	0.4195	0.0054
U33	0.0006	0.0004	0.0492	0.0184	0.3904	0.0006

Table S5: The relative information of perturbed metabolic pathways in plasma and urine samples.

Pathway name	Match Status	*P*	-log(*P*)	FDR	Impact
Steroid hormone biosynthesis	21/70	3.0297E-19	42.641	2.454E-17	0.41534
Linoleic acid metabolism	3/5	1.7723E-4	8.6381	0.0071776	1.0
Arachidonic acid metabolism	6/36	2.8367E-4	8.1677	0.0076591	0.42491
Retinol metabolism	4/17	8.4926E-4	7.0711	0.017198	0.37113
Alpha-linolenic acid metabolism	3/9	0.0013803	6.5854	0.022361	1.0
Glycerophospholipid metabolism	1/30	0.56531	0.57039	1.0	0.13889
Tryptophan metabolism	2/41	0.30608	1.1839	1.0	0.09149
Biosynthesis of unsaturated fatty acids	3/42	0.10212	2.2816	1.0	0.0
Steroid biosynthesis	1/35	0.62234	0.47427	1.0	0.0083

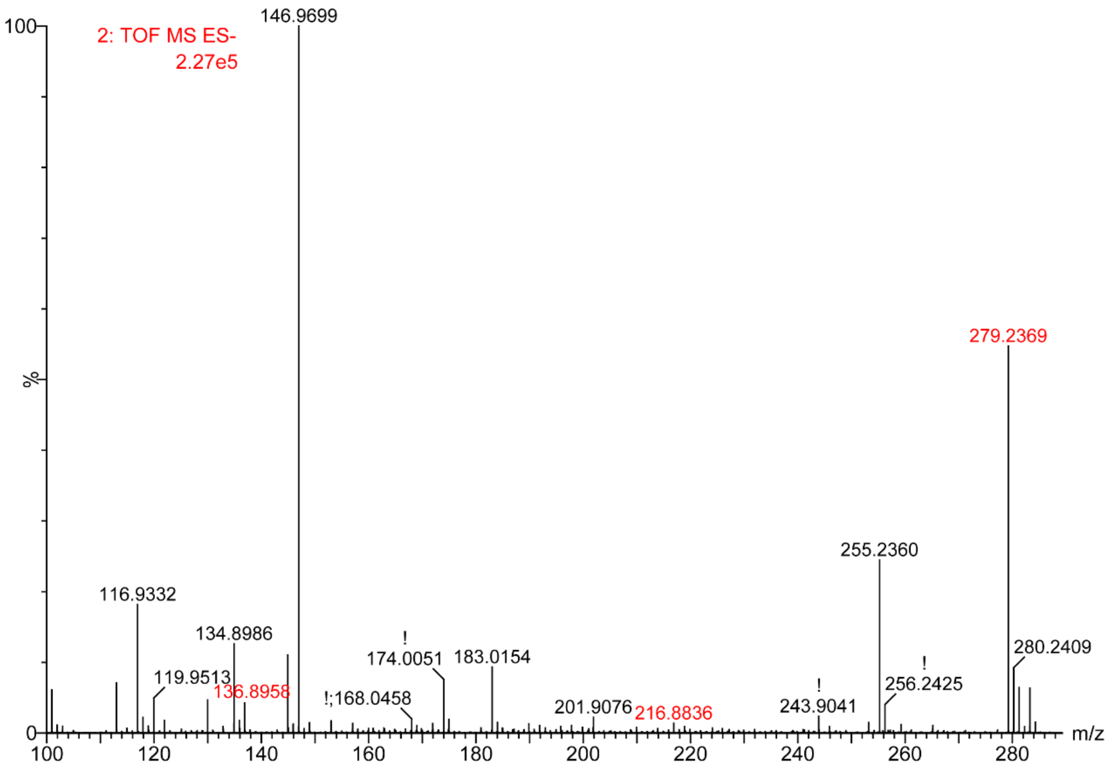

P1: Linoleic acid

Figure S2: MS/MS spectra of the identified potential marker Linoleic acid in plasma samples.

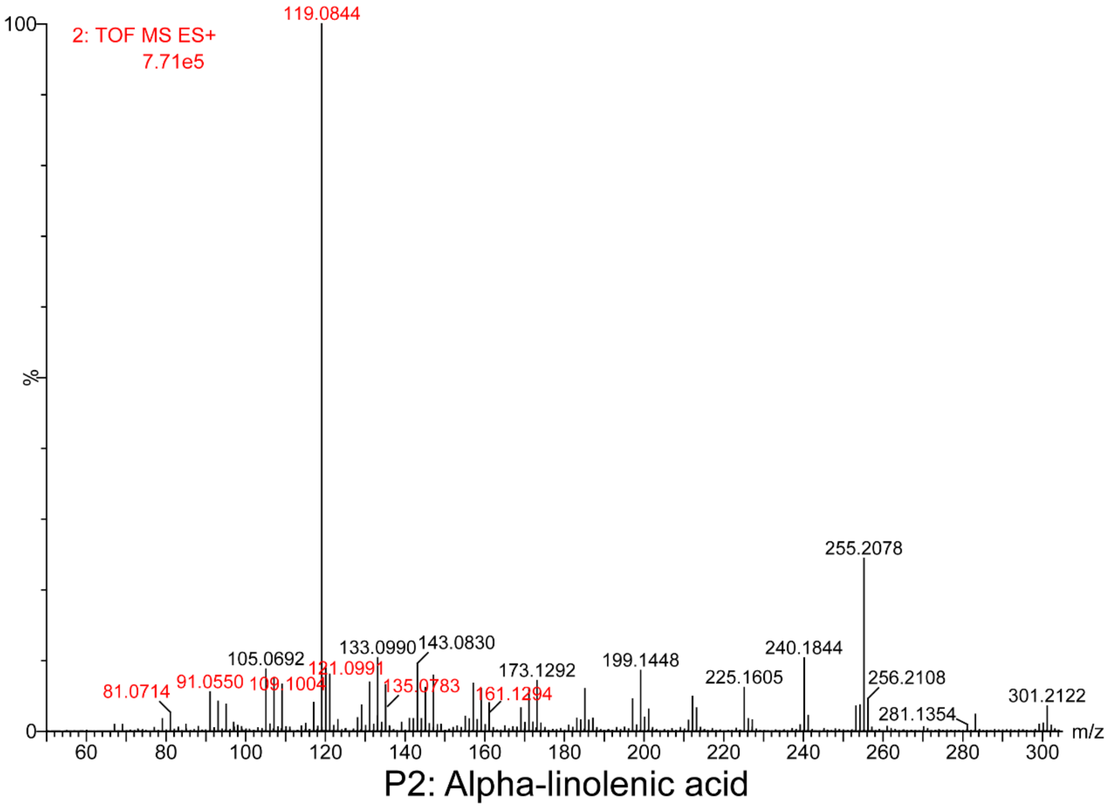

Figure S3: MS/MS spectra of the identified potential marker Alpha-linolenic acid in plasma samples.

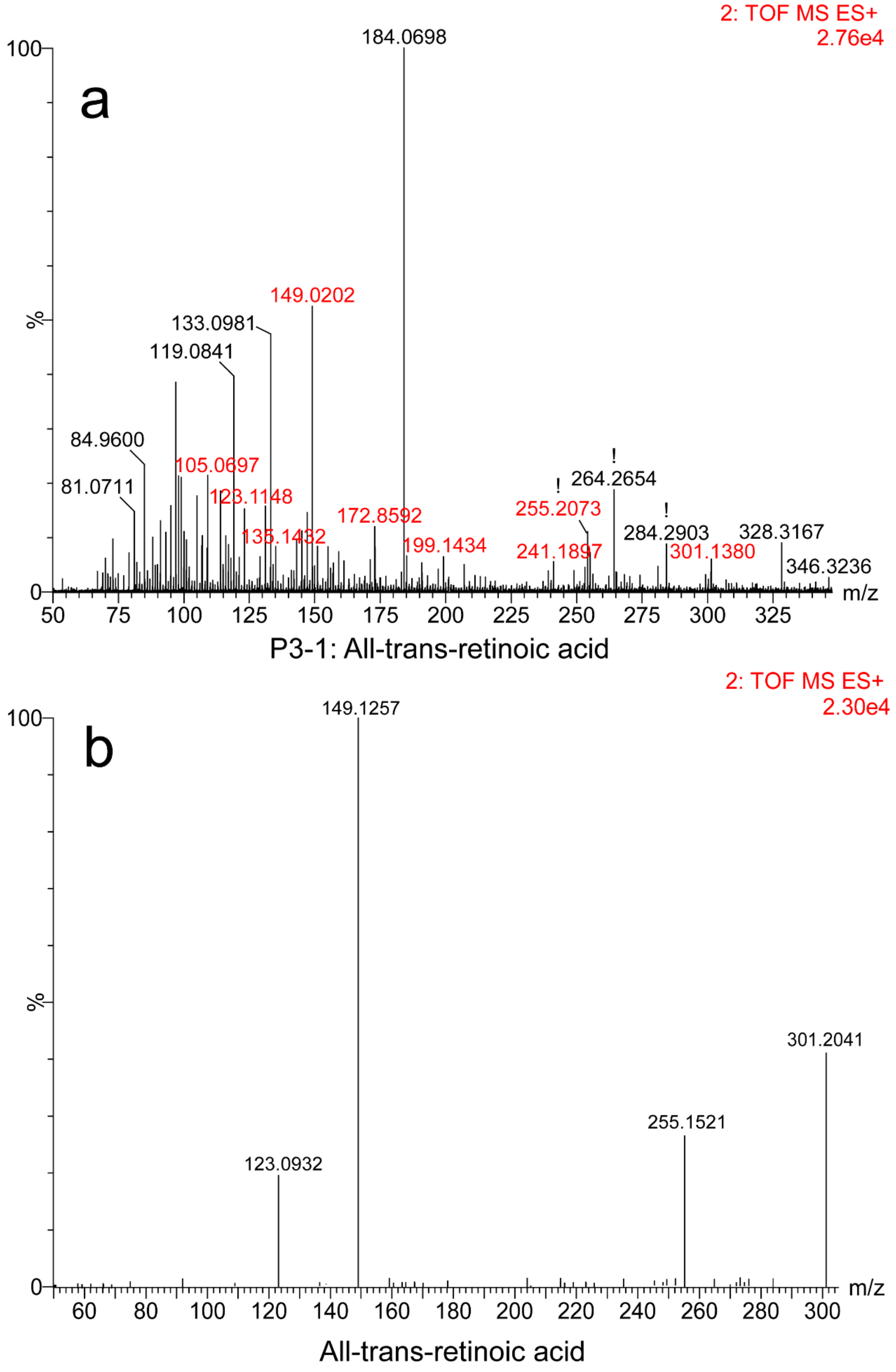

Figure S4: (a) MS/MS spectra of the identified potential marker All-trans-retinoic acid in plasma samples. (b) The reference standard chemical spectra of All-trans-retinoic acid in experimental condition.

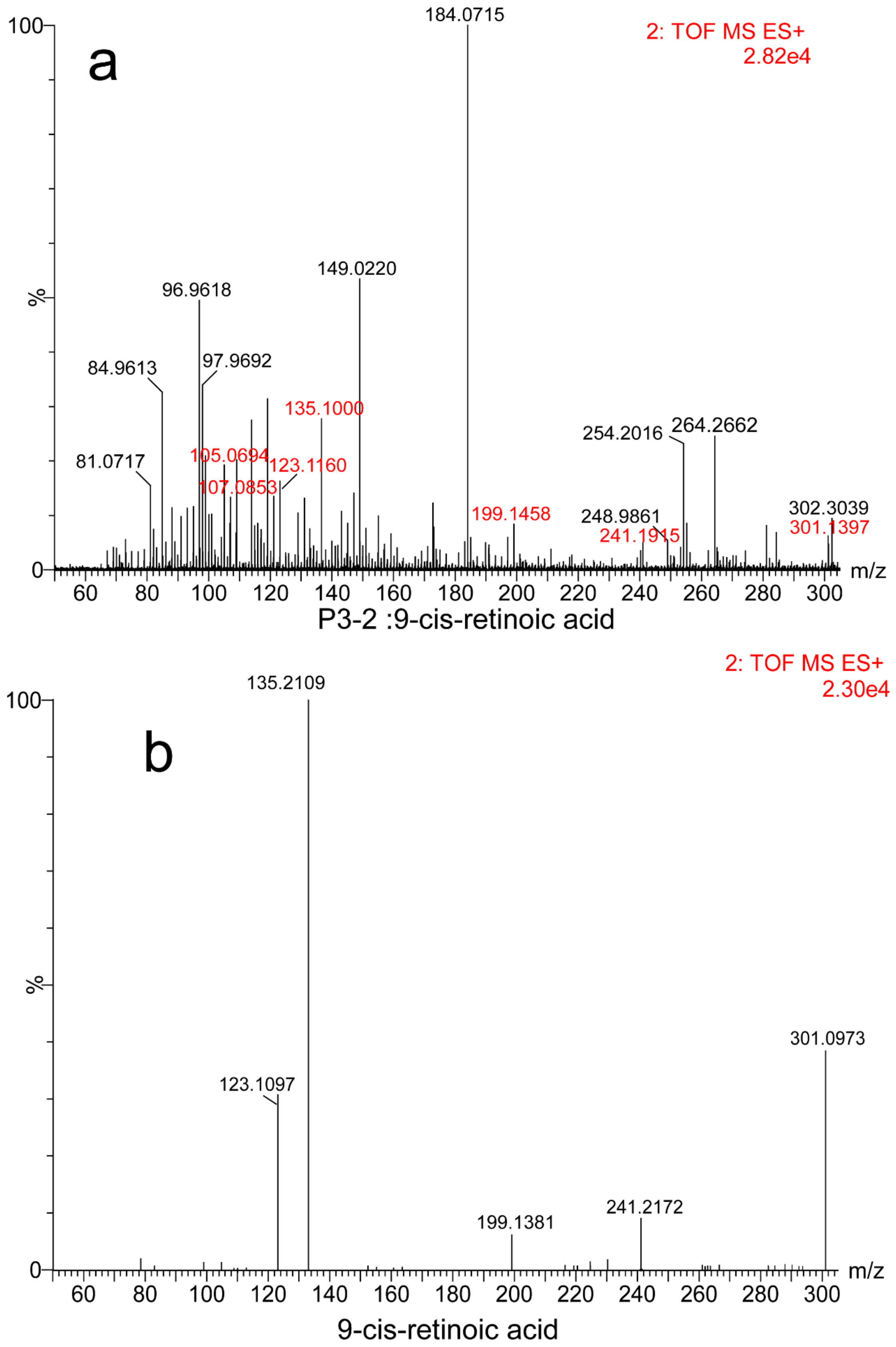

Figure S5: (a) MS/MS spectra of the identified potential marker 9-cis-retinoic acid in plasma samples. (b) The reference standard chemical spectra of 9-cis-retinoic acid in experimental condition.

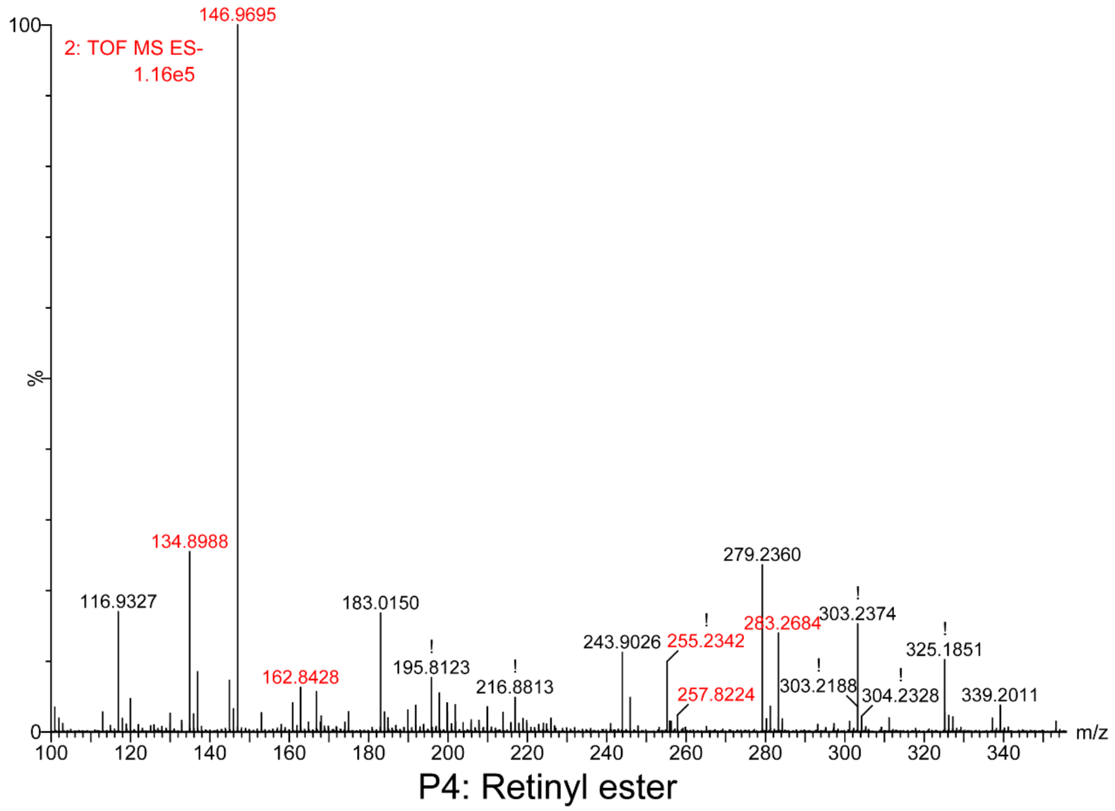

Figure S6: MS/MS spectra of the identified potential marker Retinyl ester in plasma samples.

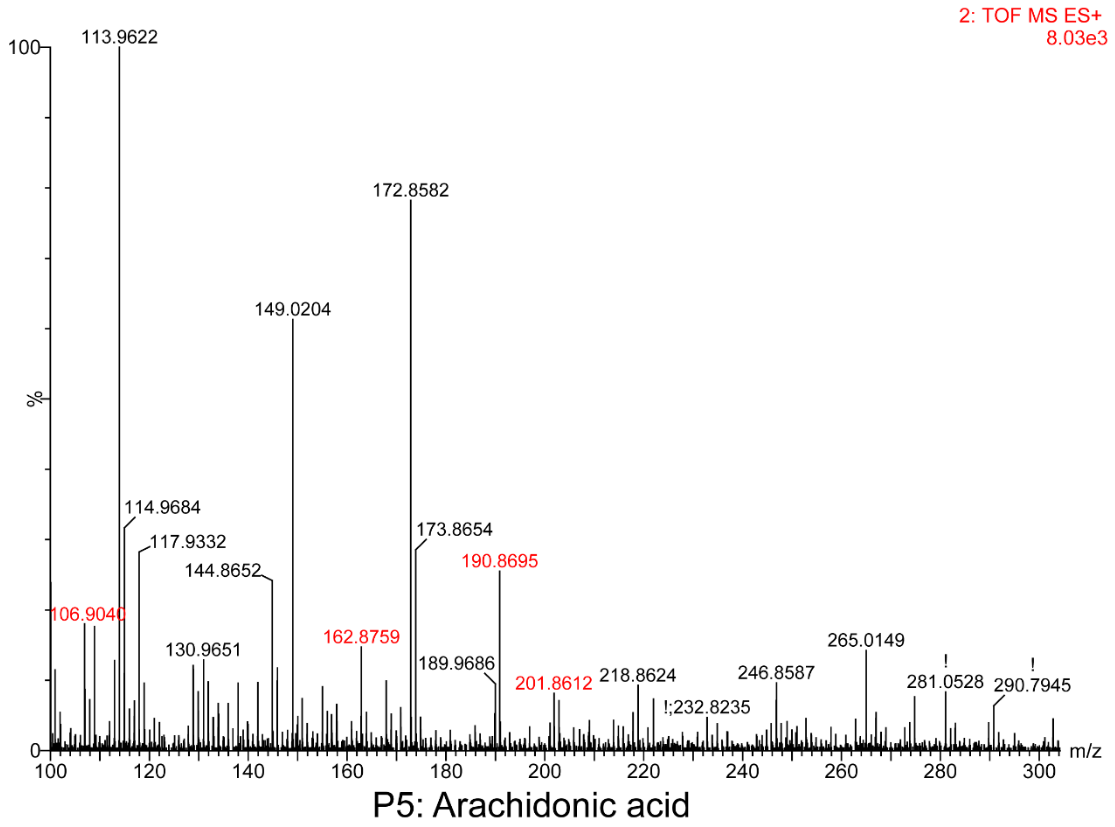

Figure S7: MS/MS spectra of the identified potential marker Arachidonic acid in plasma samples.

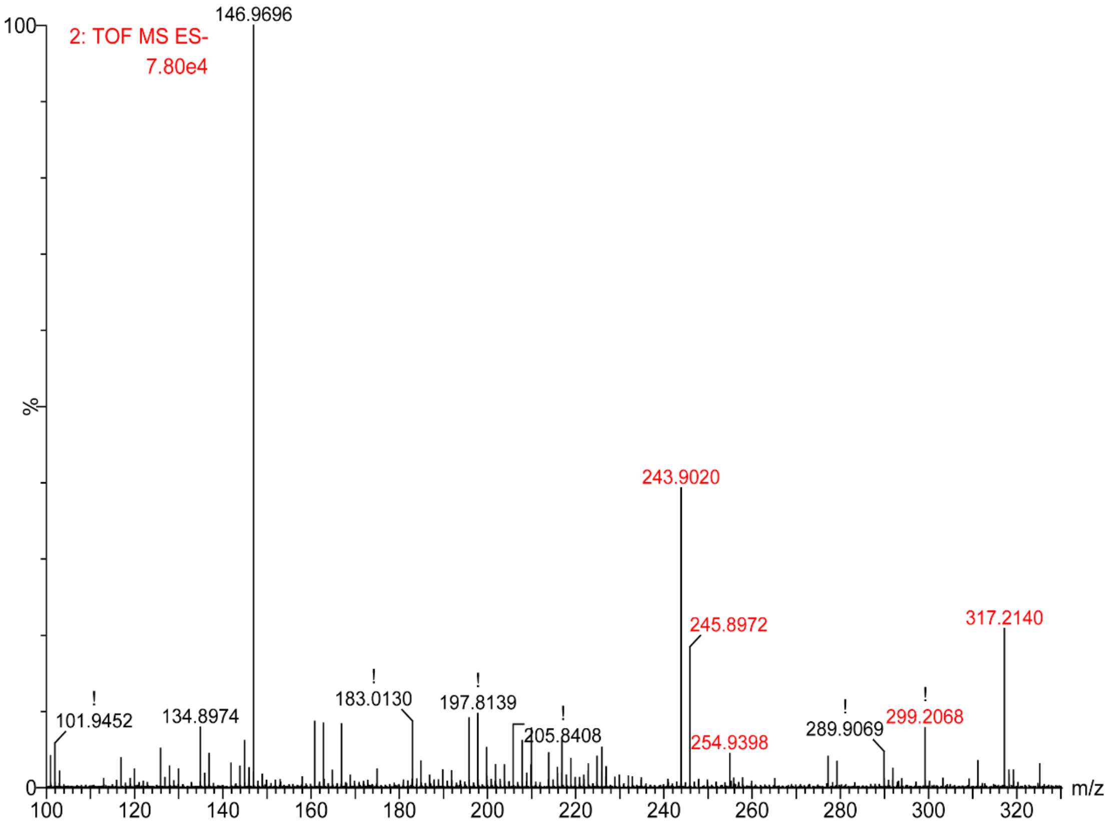

P6: Leukotriene a4

Figure S8: MS/MS spectra of the identified potential marker Leukotriene a4 in plasma samples.

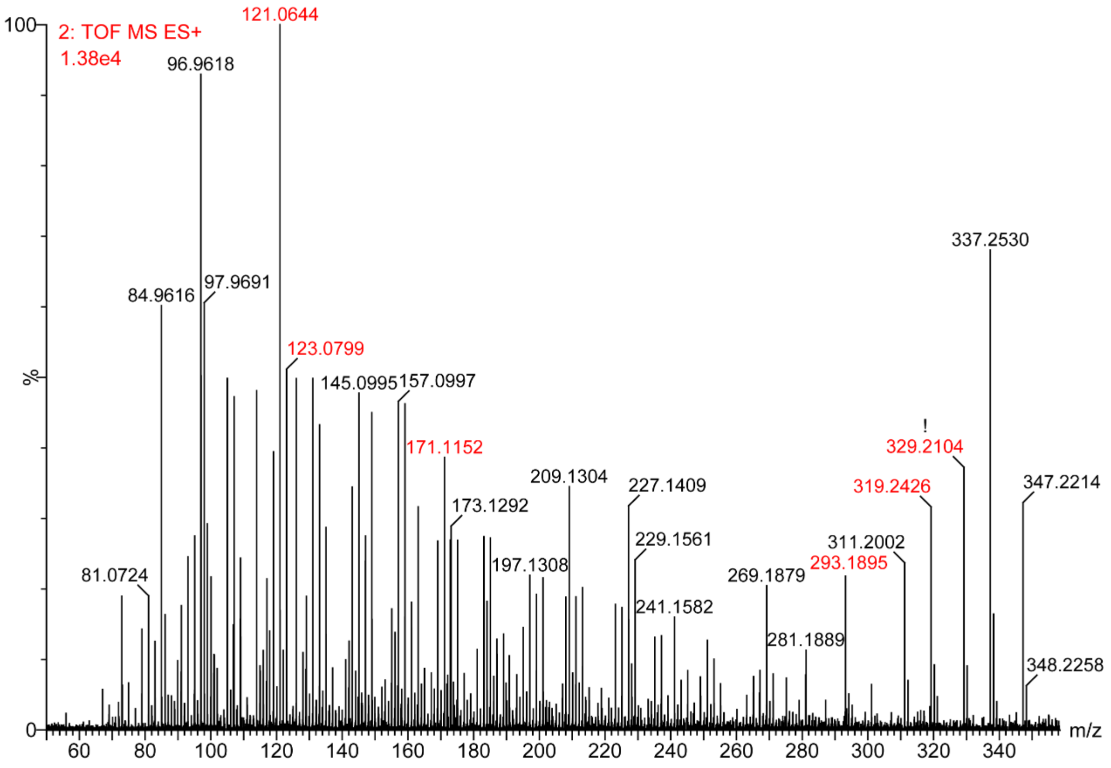

P7: Cortexolone

Figure S9: MS/MS spectra of the identified potential marker Cortexolone in plasma samples.

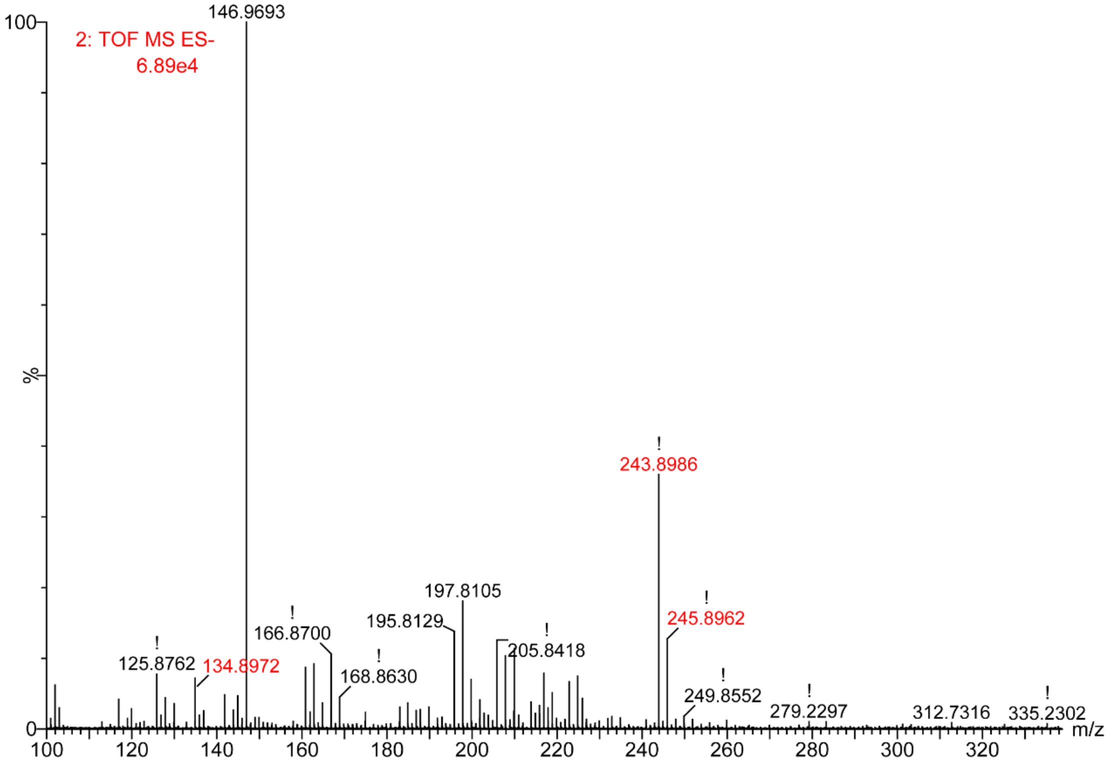

P8: 21-deoxycortisol

Figure S10: MS/MS spectra of the identified potential marker 21-deoxycortisol in plasma samples.

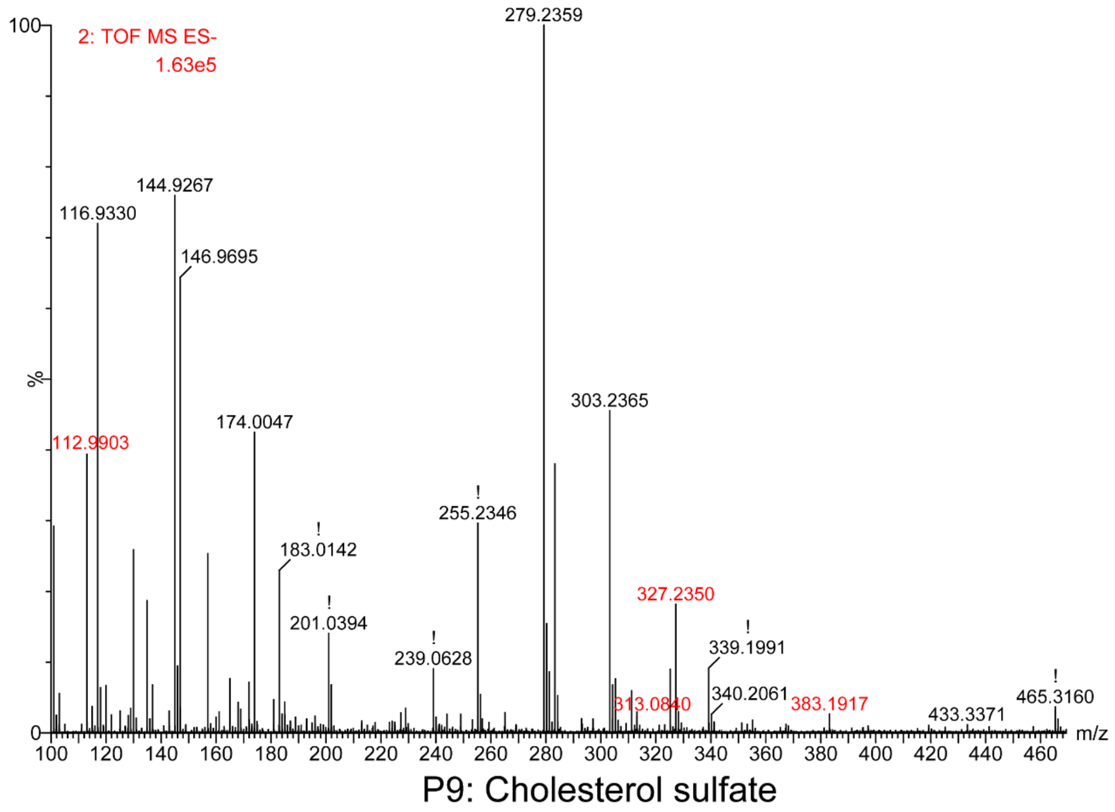

Figure S11: MS/MS spectra of the identified potential marker Cholesterol sulfate in plasma samples.

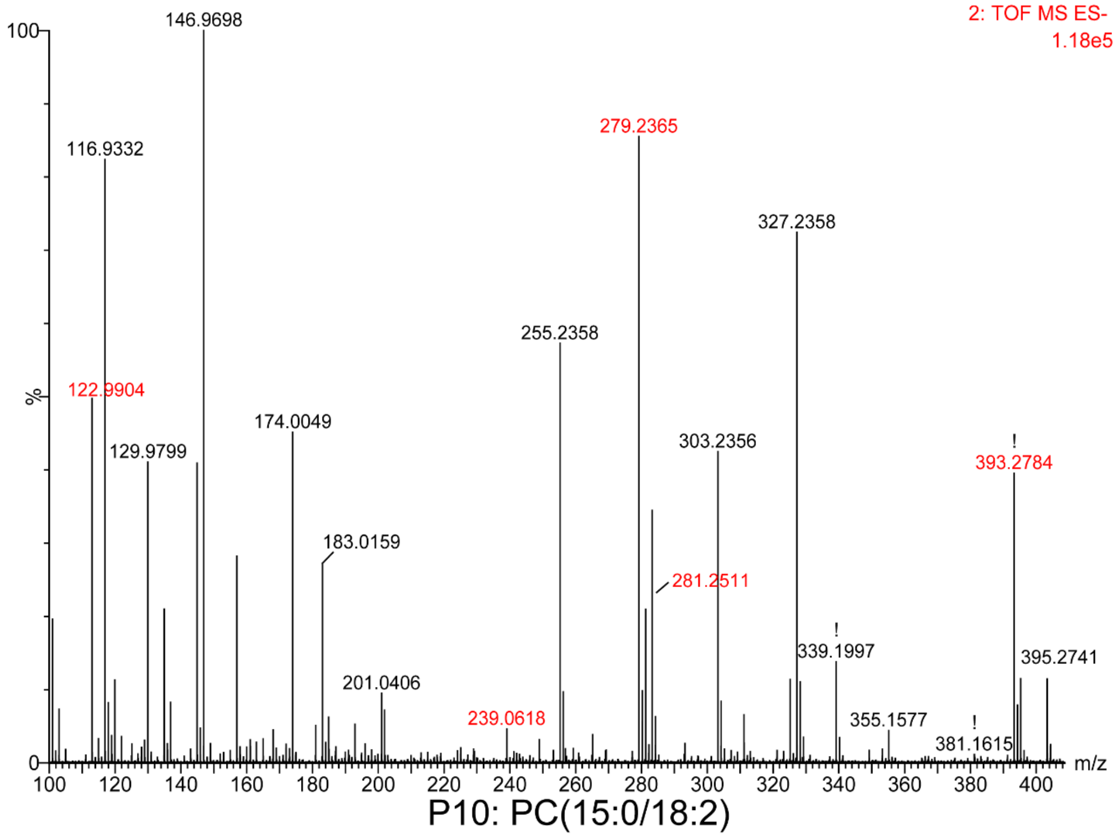

Figure S12: MS/MS spectra of the identified potential marker PC (15:0/18:2) in plasma samples.

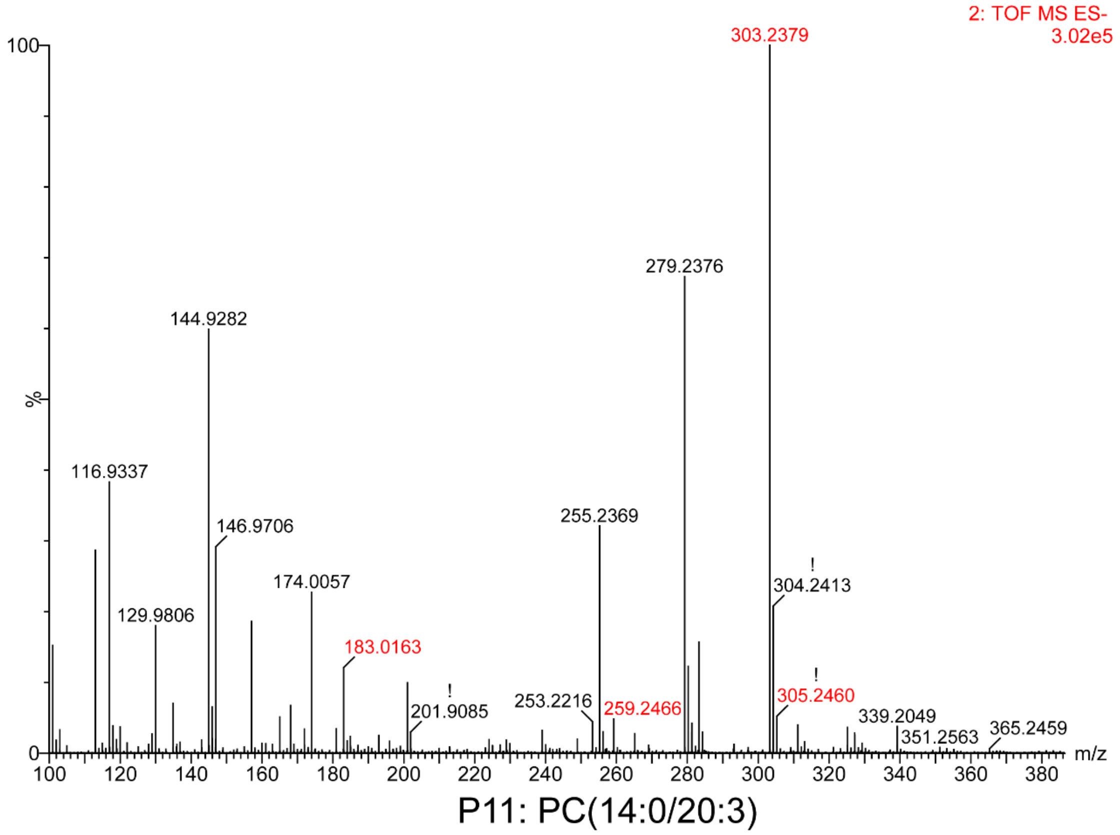

Figure S13: MS/MS spectra of the identified potential marker PC (14:0/20:3) in plasma samples.

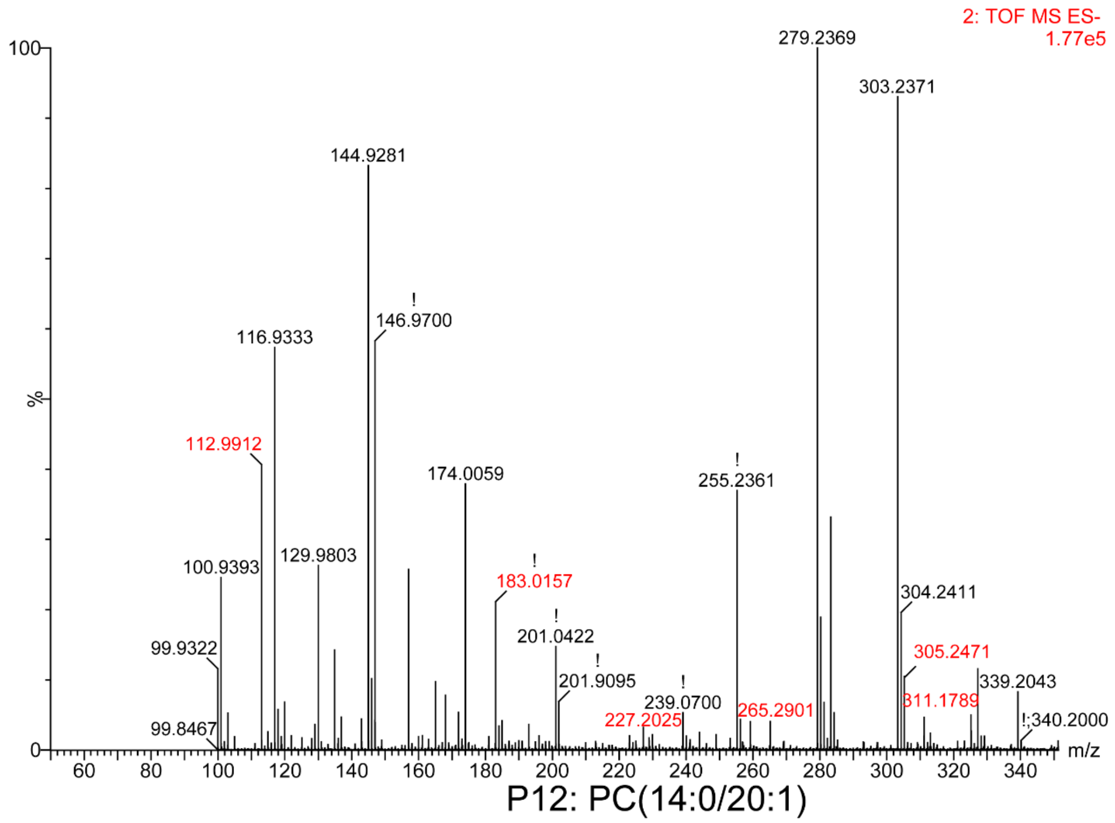

Figure S14: MS/MS spectra of the identified potential marker PC (14:0/20:1) in plasma samples.

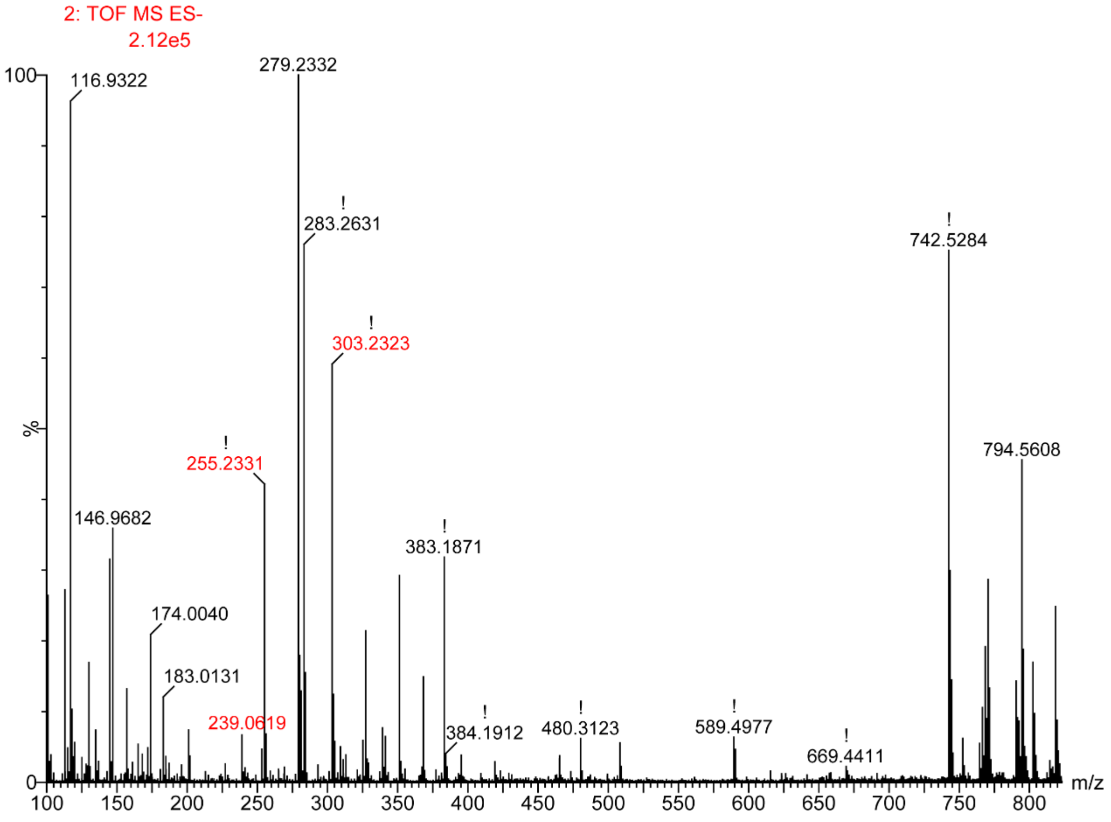

P13: PC(16:0/20:4)

Figure S15: MS/MS spectra of the identified potential marker PC (16:0/20:4) in plasma samples.

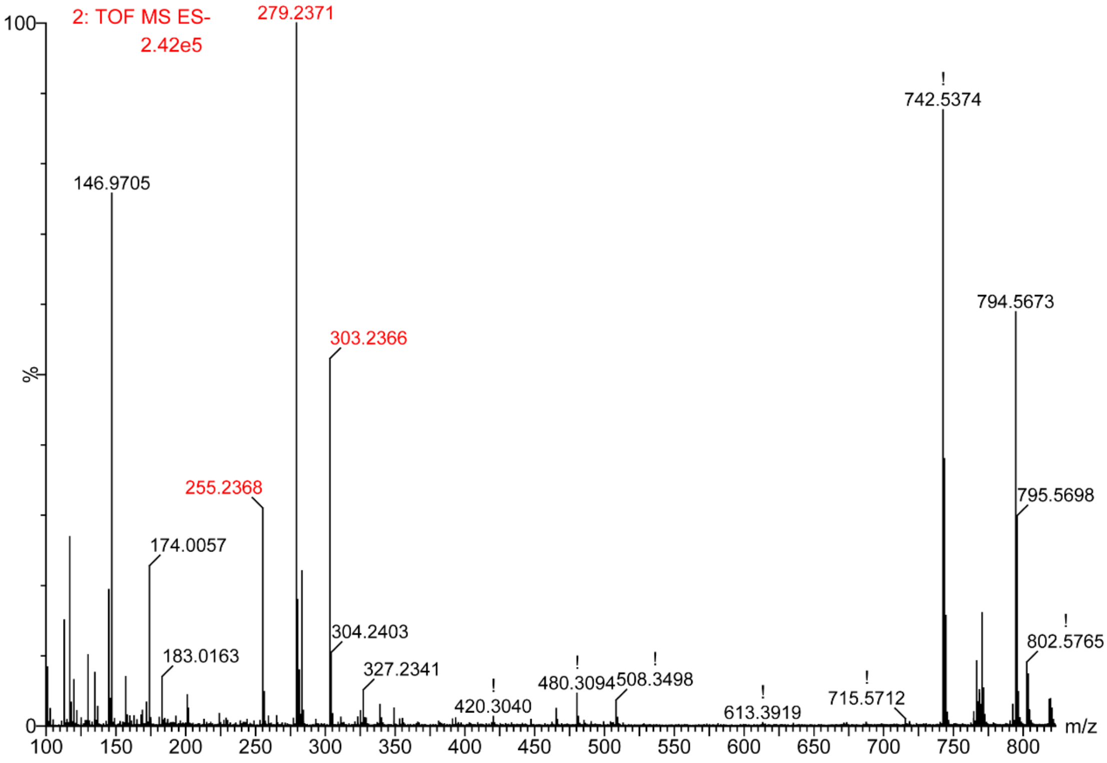

P14: PC(20:4/18:0)

Figure S16: MS/MS spectra of the identified potential marker PC (20:4/18:0) in plasma samples.

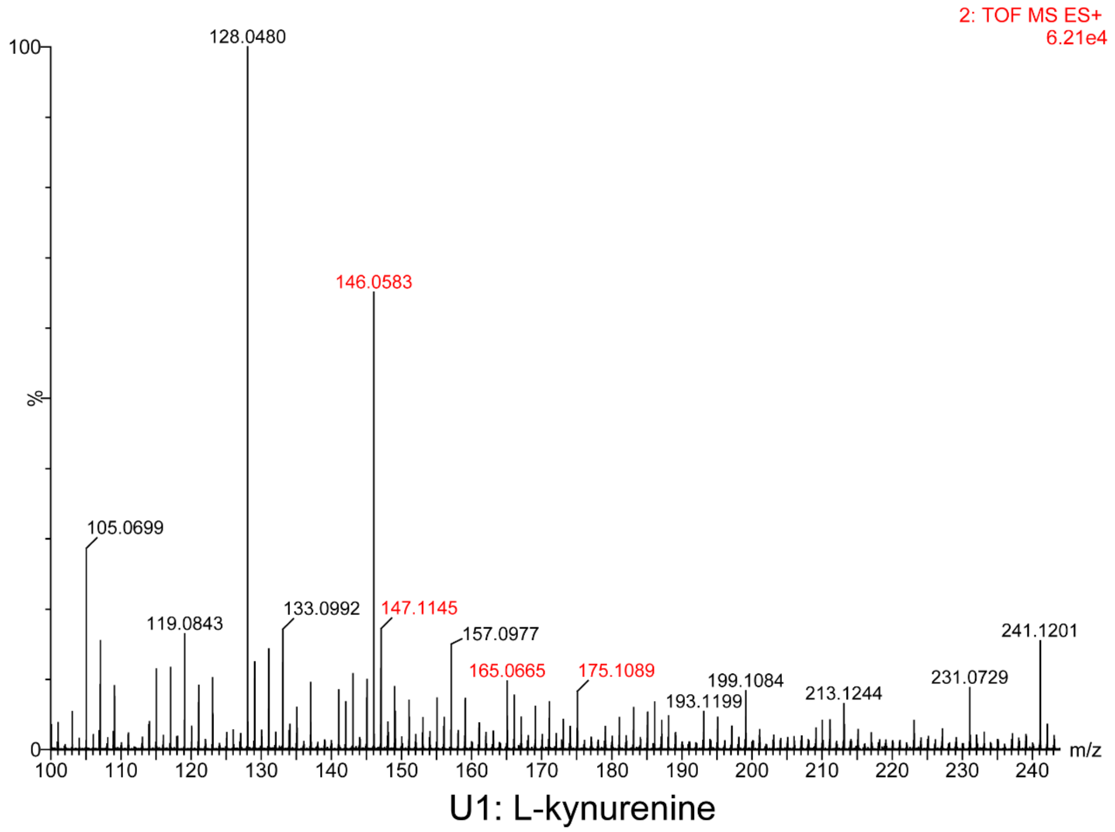

Figure S17: MS/MS spectra of the identified potential marker L-kynurenine in urine samples.

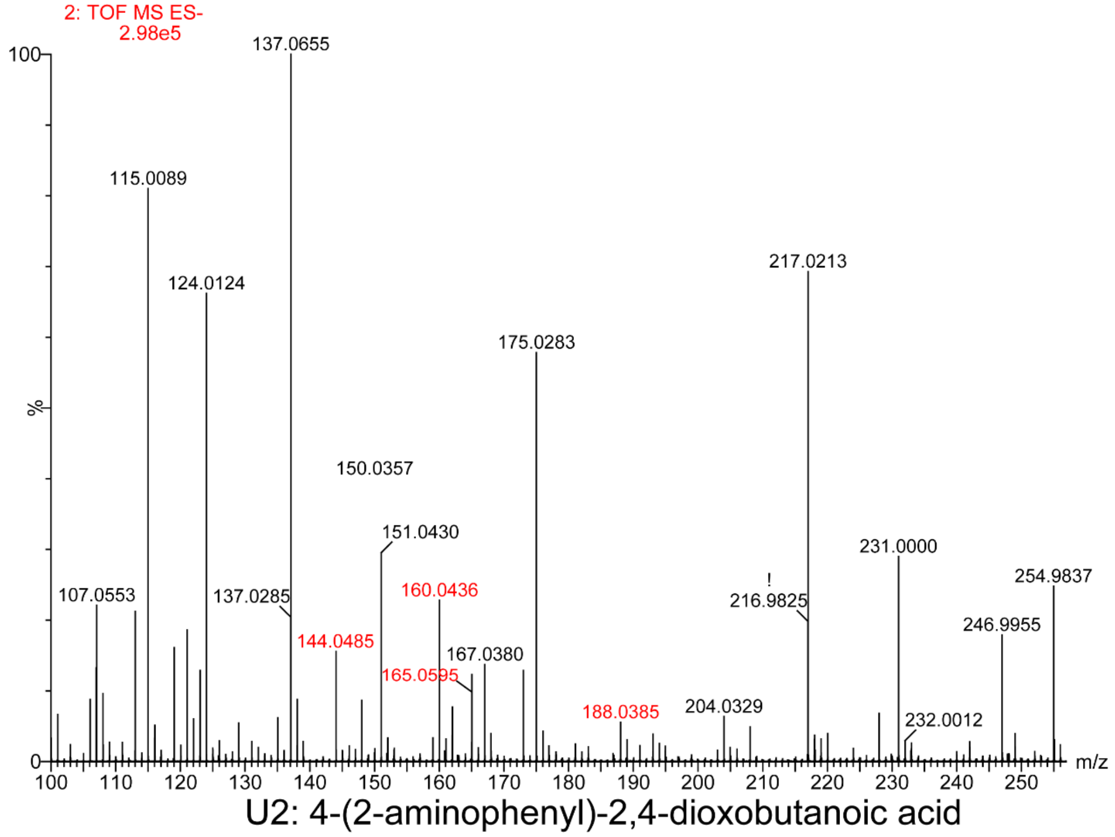

Figure S18: MS/MS spectra of the identified potential marker 4-(2-aminophenyl)-2,4-dioxobutanoic acid in urine samples.

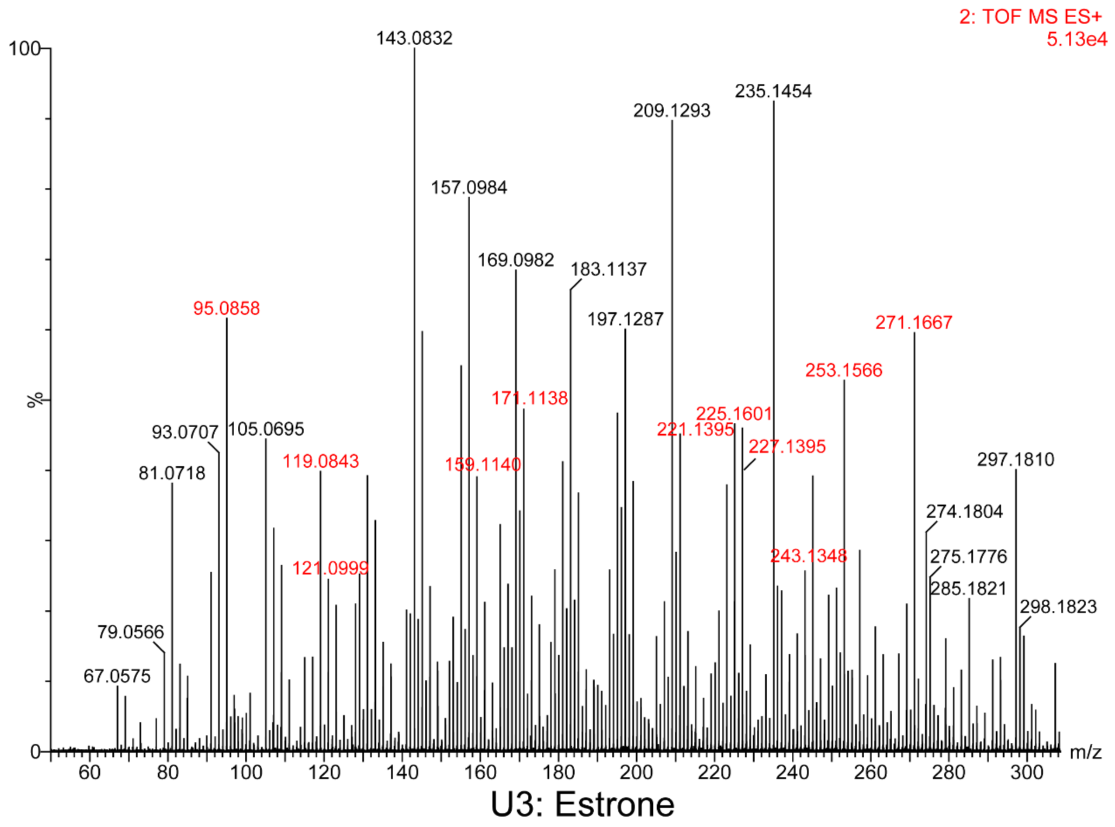

Figure S19: MS/MS spectra of the identified potential marker Estrone in urine samples.

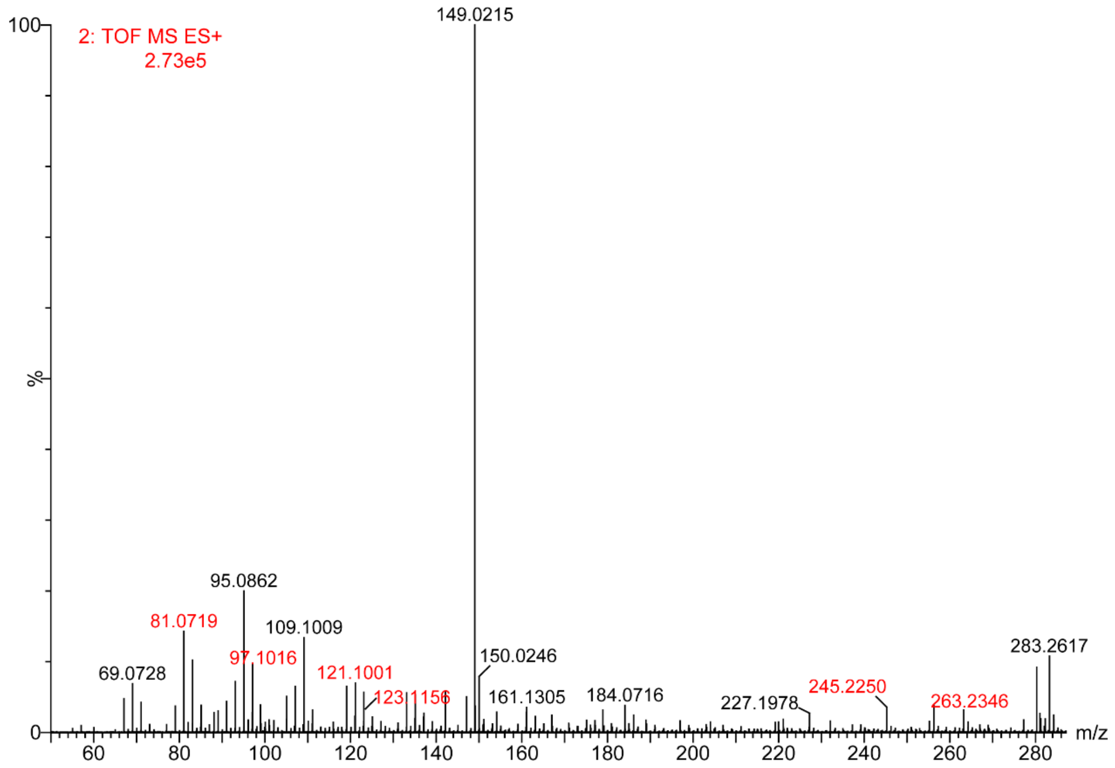

U4: Linoleic acid

Figure S20: MS/MS spectra of the identified potential marker Linoleic acid in urine samples.

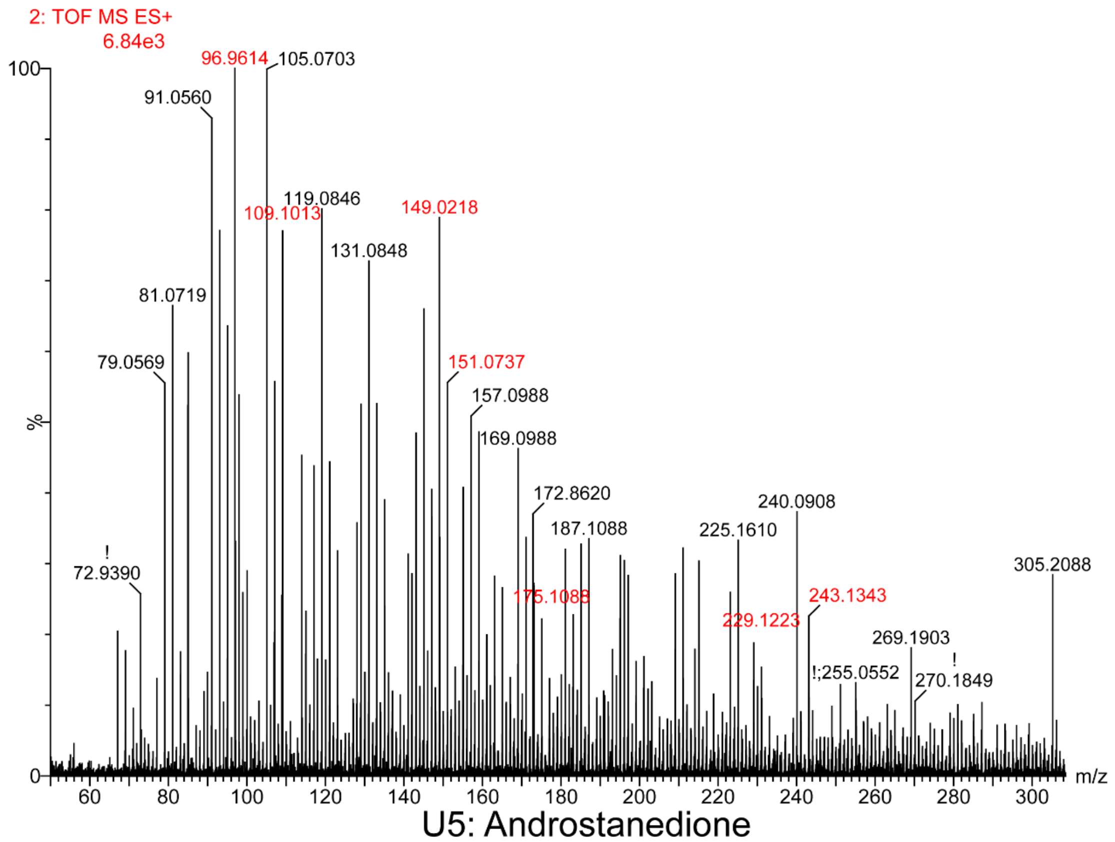

U5: Androstanedione

Figure S21: MS/MS spectra of the identified potential marker Androstanedione in urine samples.

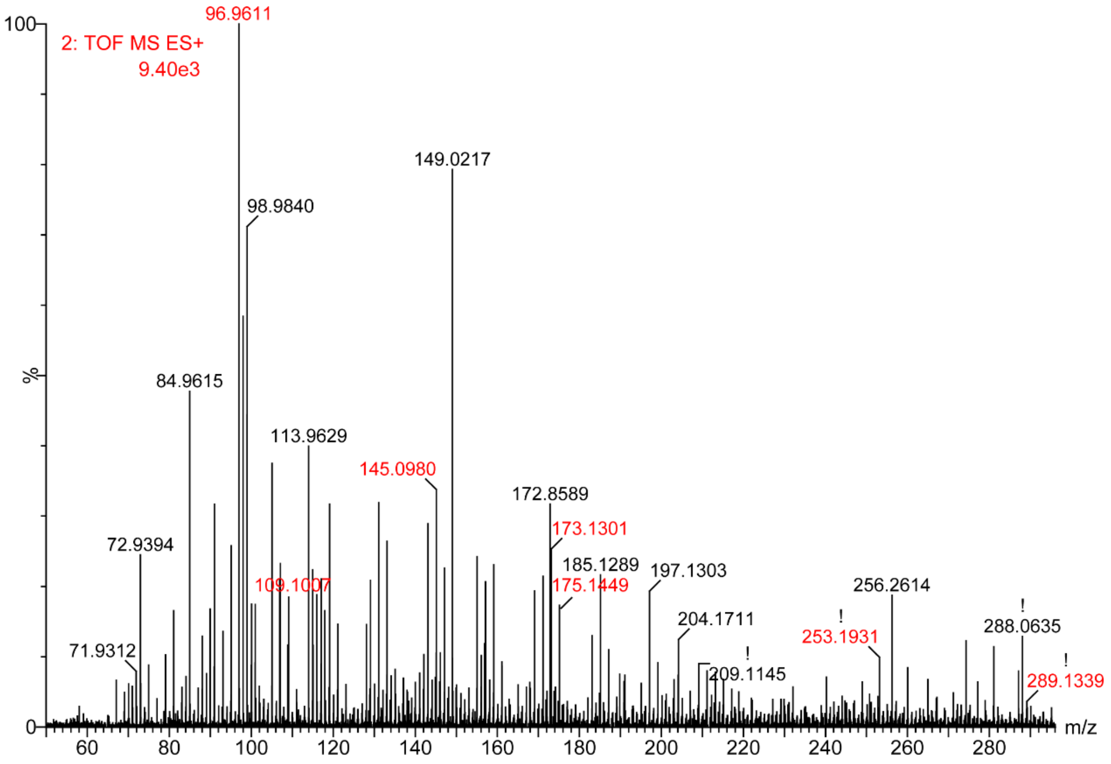

U6: Dehydroepiandrosterone

Figure S22: MS/MS spectra of the identified potential marker Dehydroepiandrosterone in urine samples.

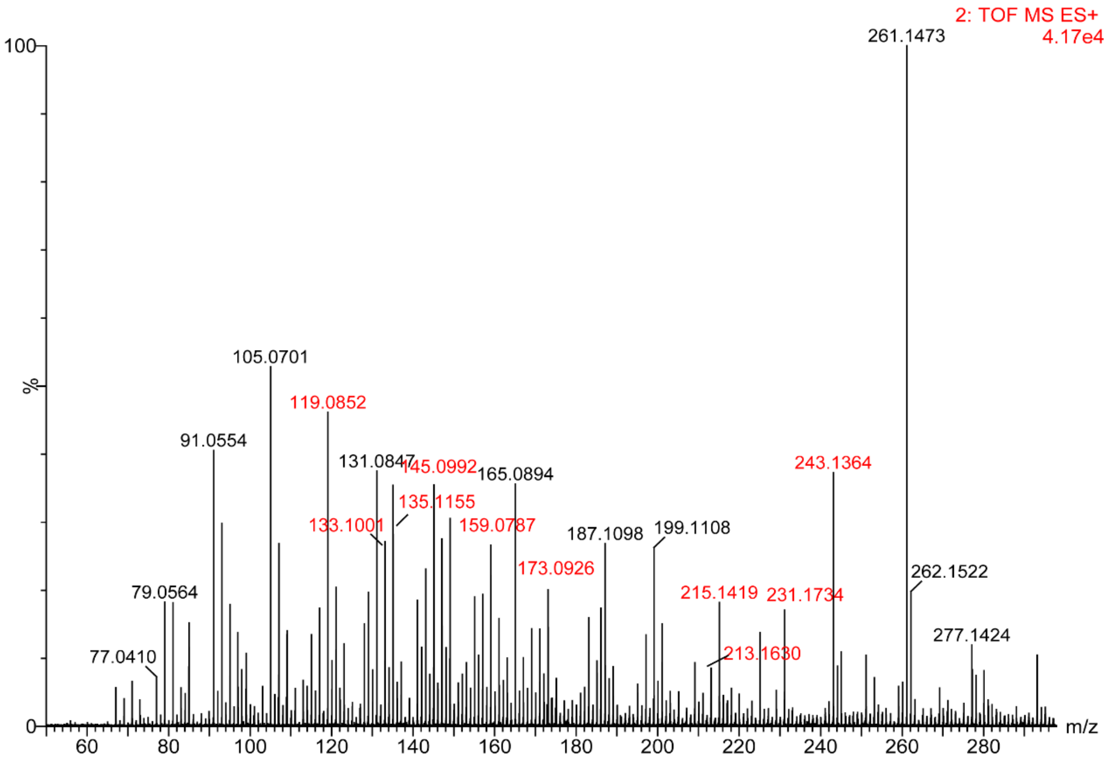

U7: Estradiol

Figure S23: MS/MS spectra of the identified potential marker Estradiol in urine samples.

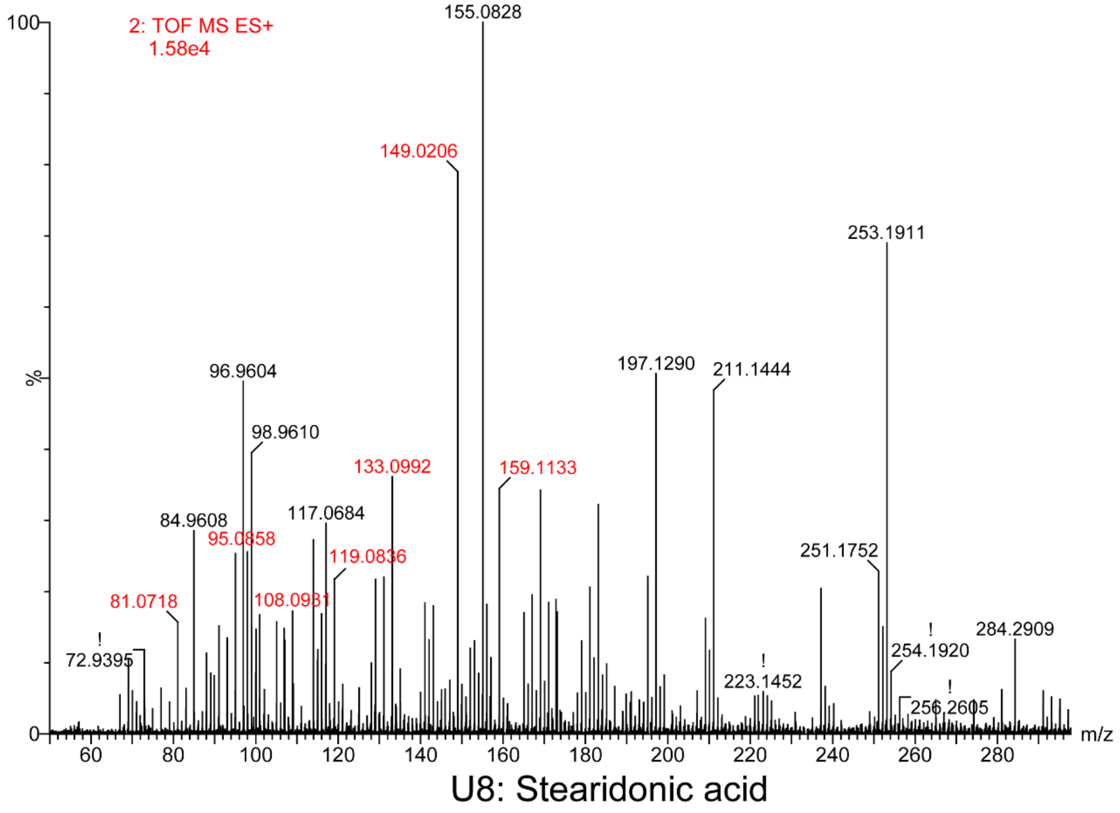

Figure S24: MS/MS spectra of the identified potential marker Stearidonic acid in urine samples.

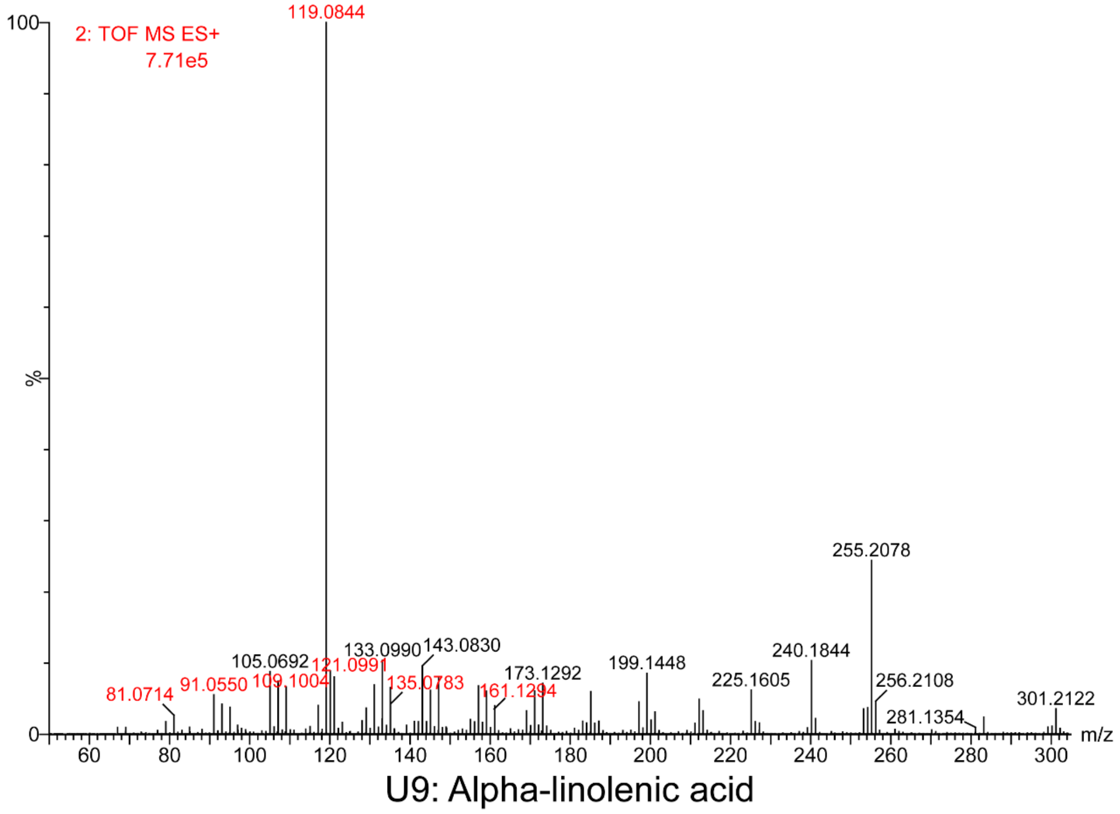

Figure S25: MS/MS spectra of the identified potential marker Alpha-linolenic acid in urine samples.

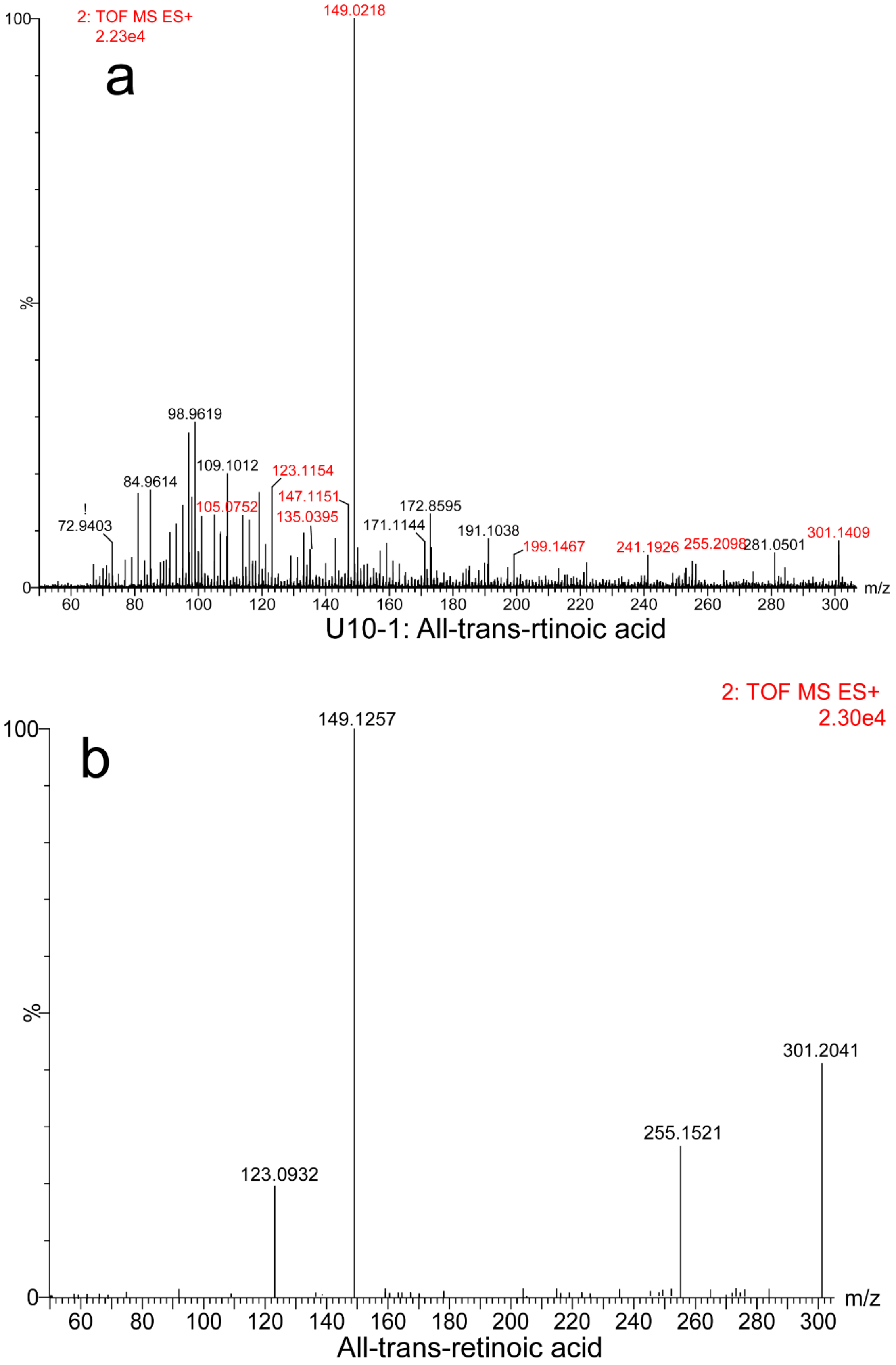

Figure S26: (a) MS/MS spectra of the identified potential marker All-trans-retinoic acid in urine samples. (b) The reference standard chemical spectra of All-trans-retinoic acid in experimental condition.

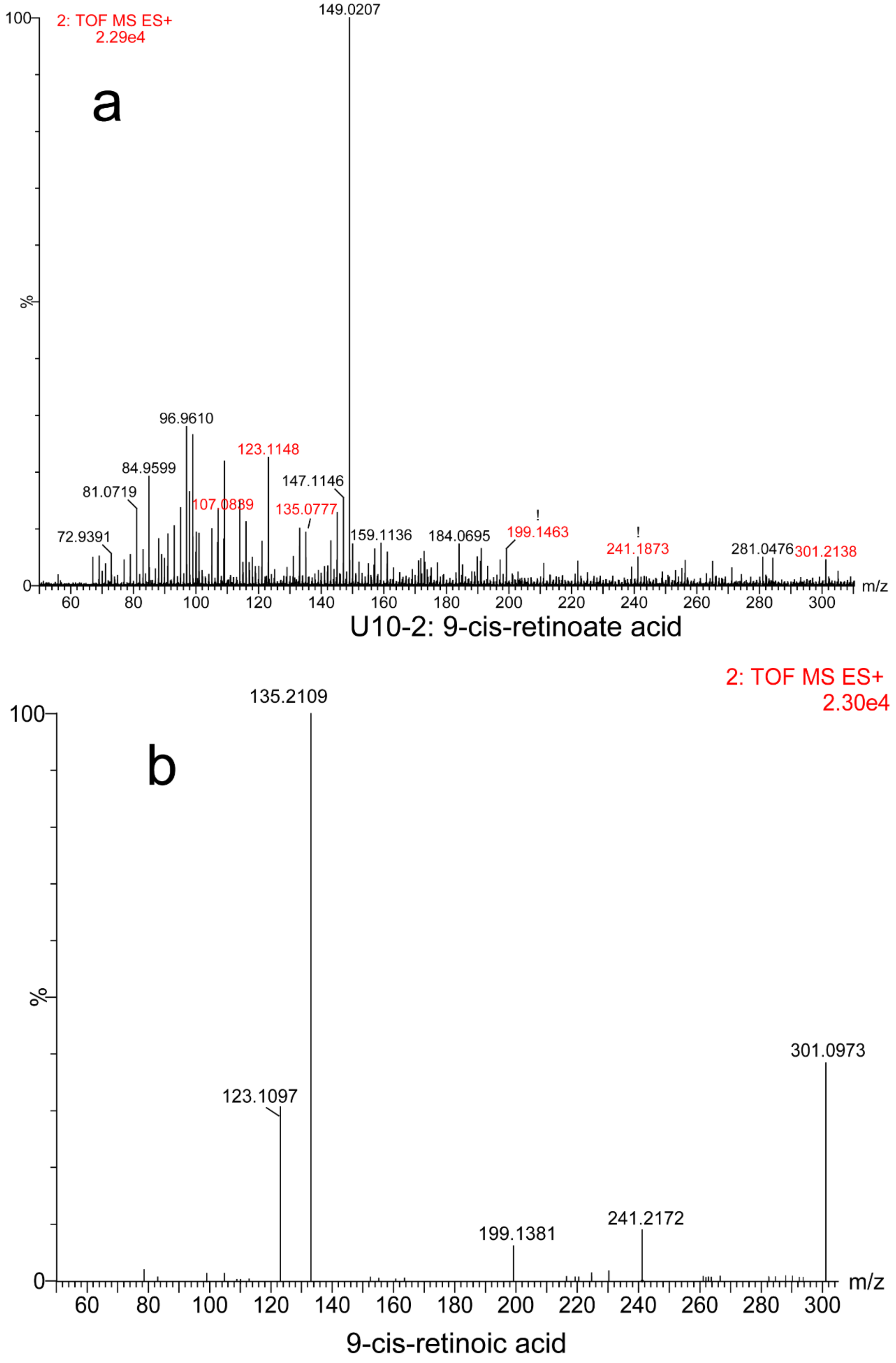

Figure S27: (a) MS/MS spectra of the identified potential marker 9-cis-retinoate acid in urine samples. (b) The reference standard chemical spectra of 9-cis-retinoate acid in experimental

condition.

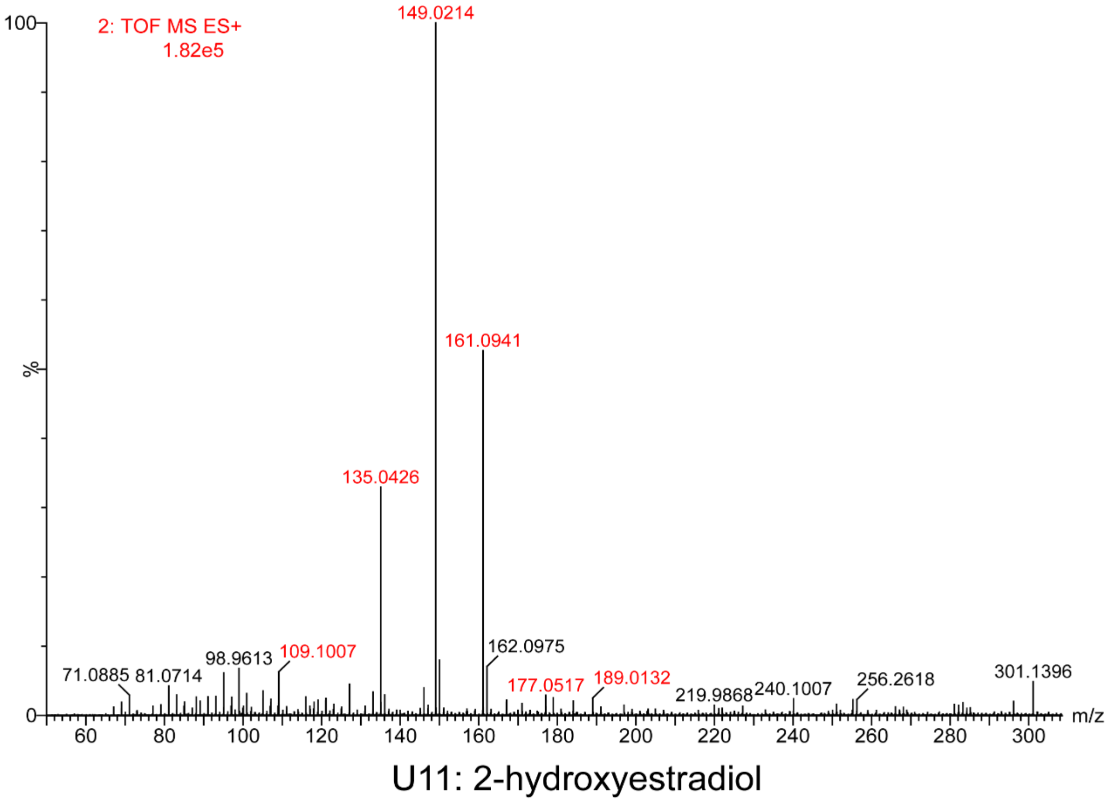

Figure S28: MS/MS spectra of the identified potential marker 2-hydroxyestradiol in urine samples.

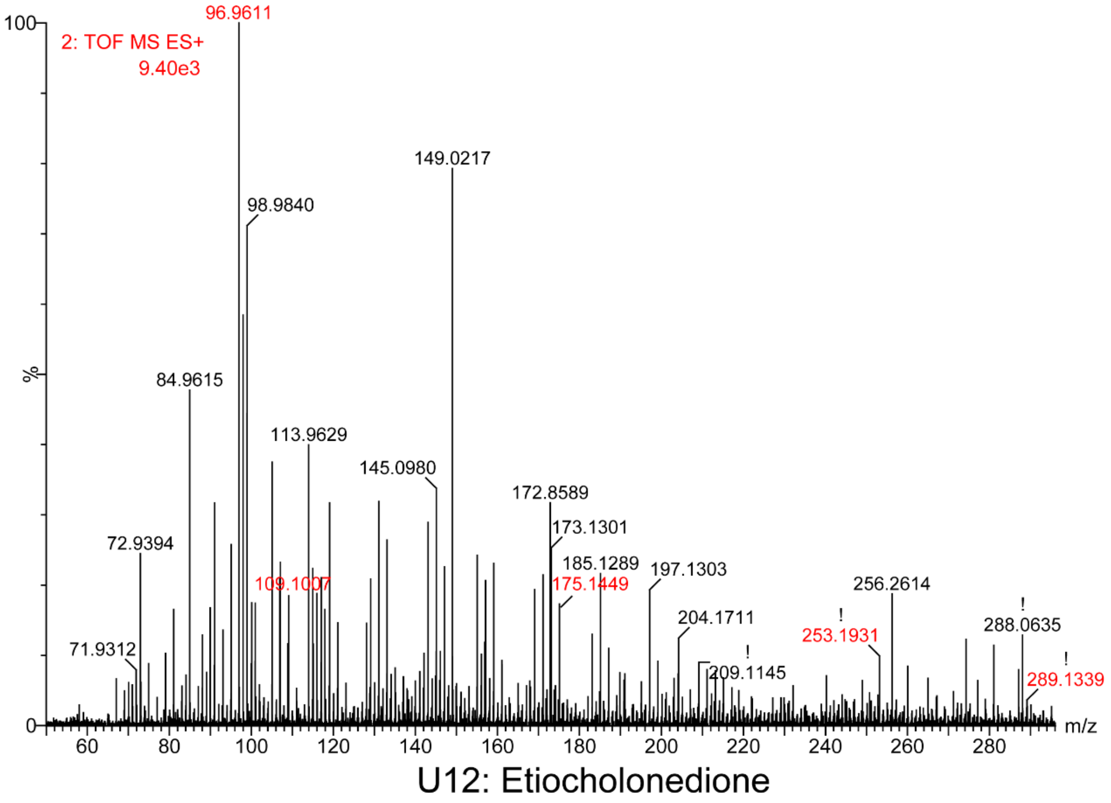

Figure S29: MS/MS spectra of the identified potential marker Etiocholonedione in urine samples.

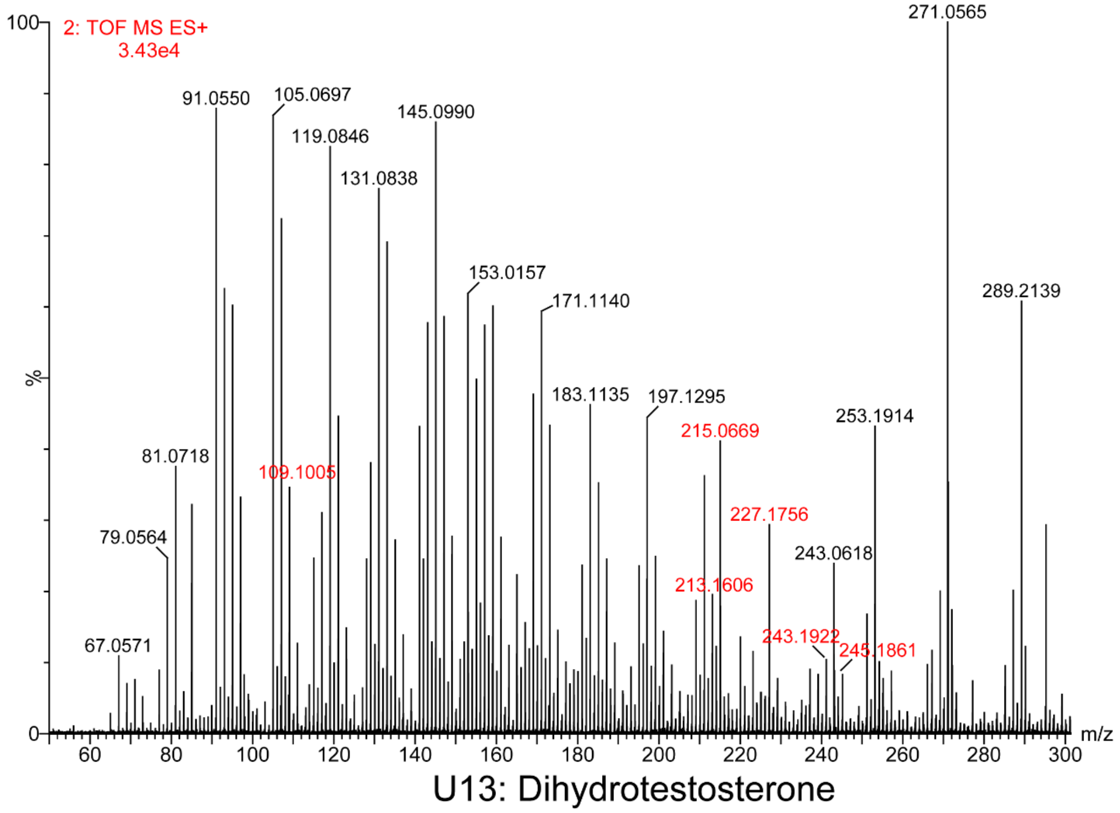

Figure S30: MS/MS spectra of the identified potential marker Dihydrotestosterone in urine samples.

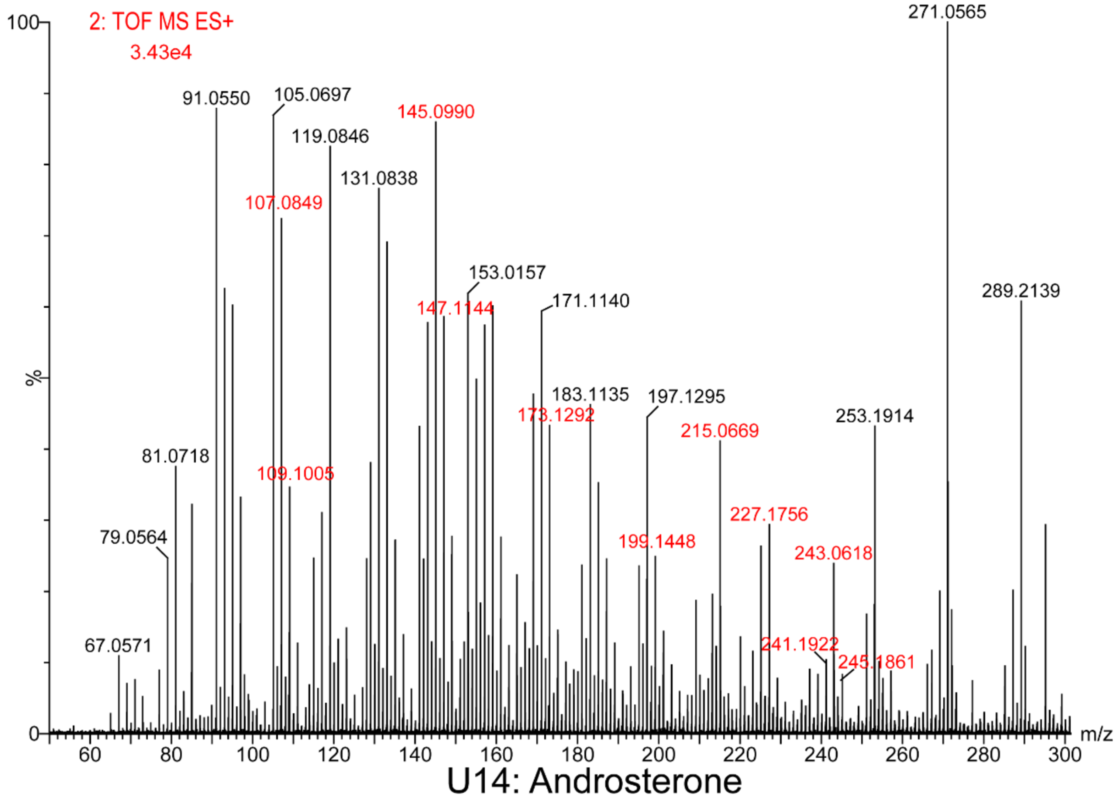

Figure S31: MS/MS spectra of the identified potential marker Androsterone in urine samples.

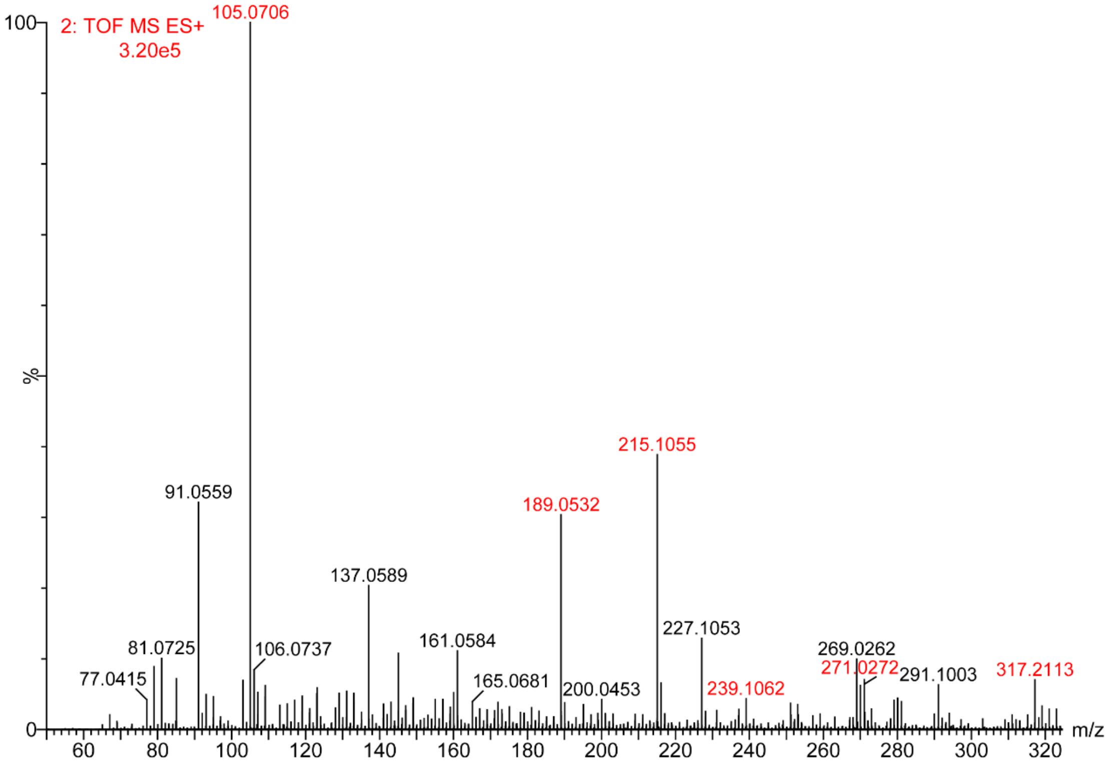

U15: Hydroxyretinoic acid

Figure S32: MS/MS spectra of the identified potential marker Hydroxyretinoic acid in urine samples.

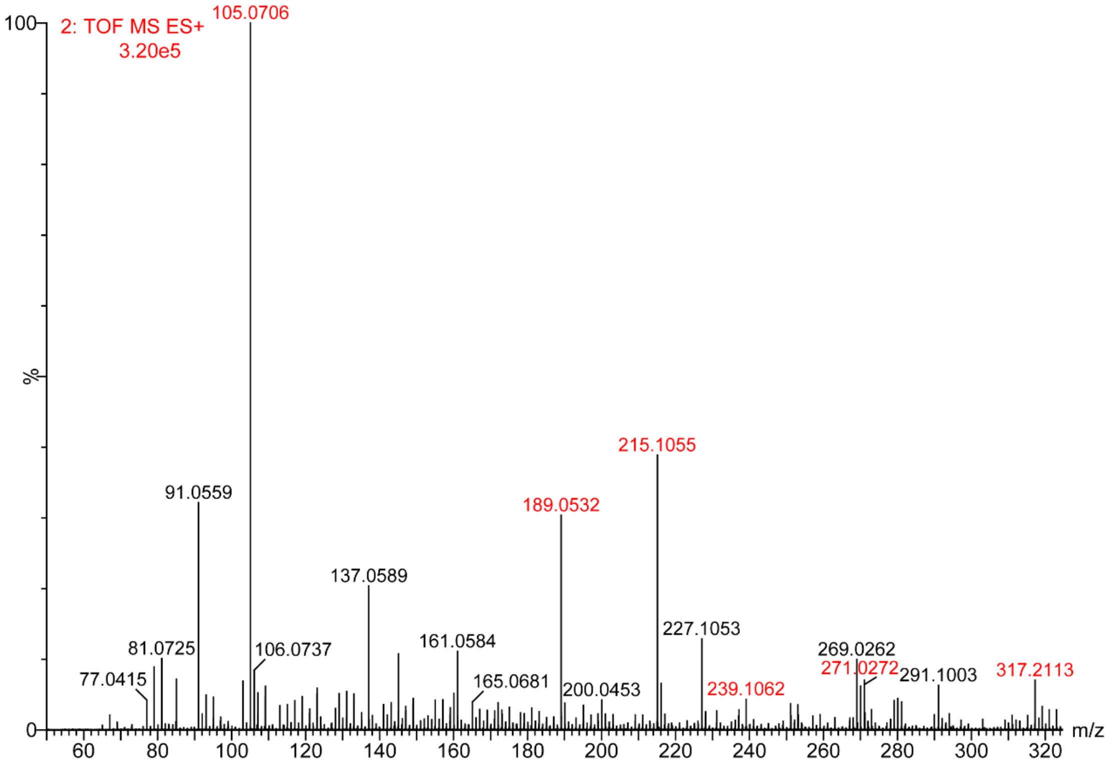

U16: All-trans-5,6-epoxyretinoic

Figure S33: MS/MS spectra of the identified potential marker All-trans-5,6-epoxyretinoic in urine samples.

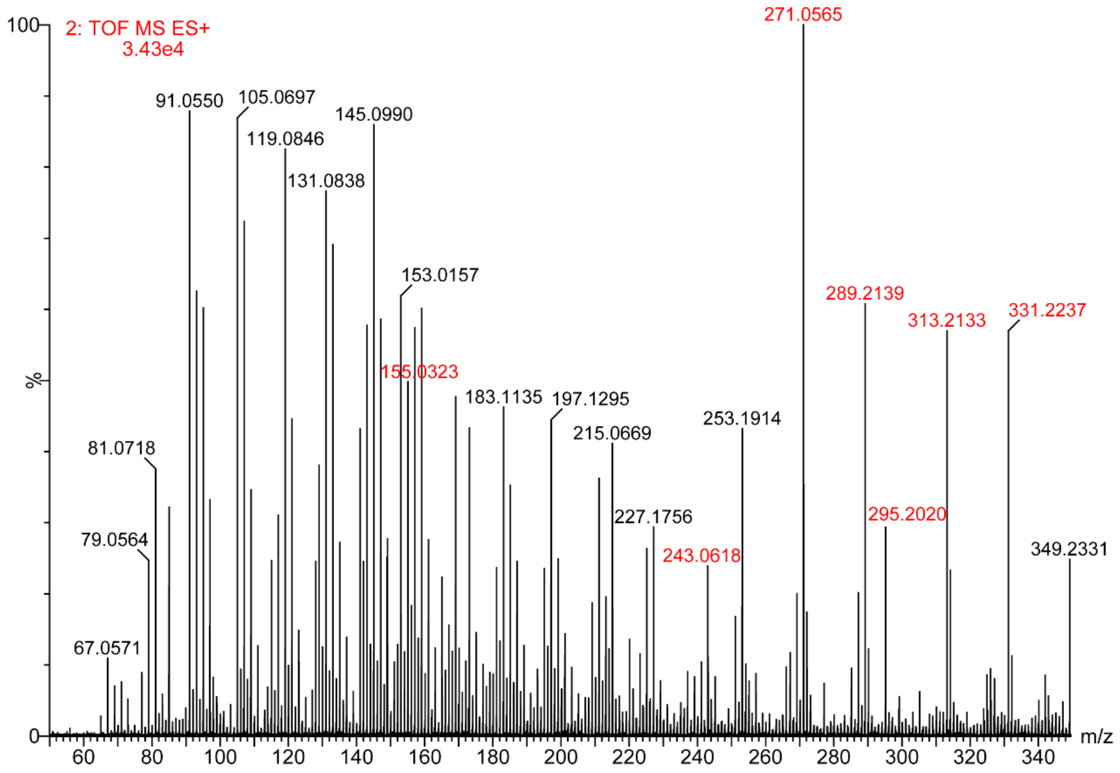

U17: Deoxycorticosterone

Figure S34: MS/MS spectra of the identified potential marker Deoxycorticosterone in urine samples.

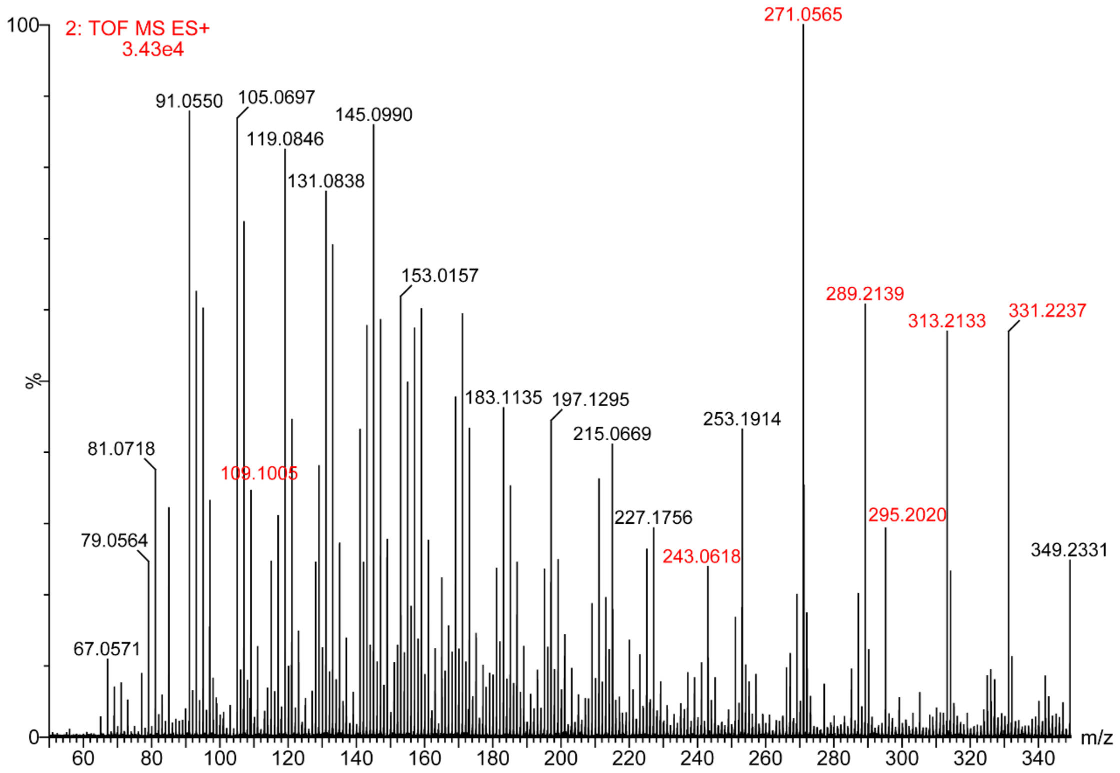

U18: 17-hydroxyprogesterone

Figure S35: MS/MS spectra of the identified potential marker 17-hydroxyprogesterone in urine

samples.

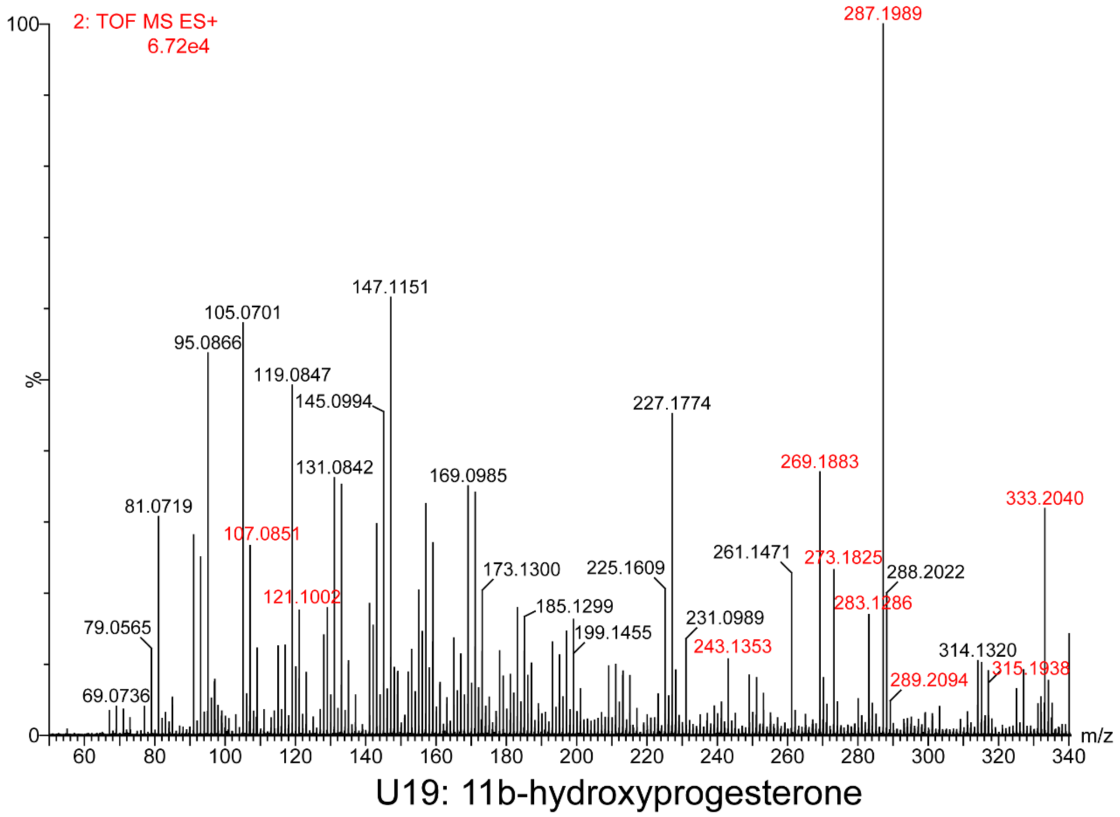

Figure S36: MS/MS spectra of the identified potential marker 11bb-hydroxyprogesterone in urine samples.

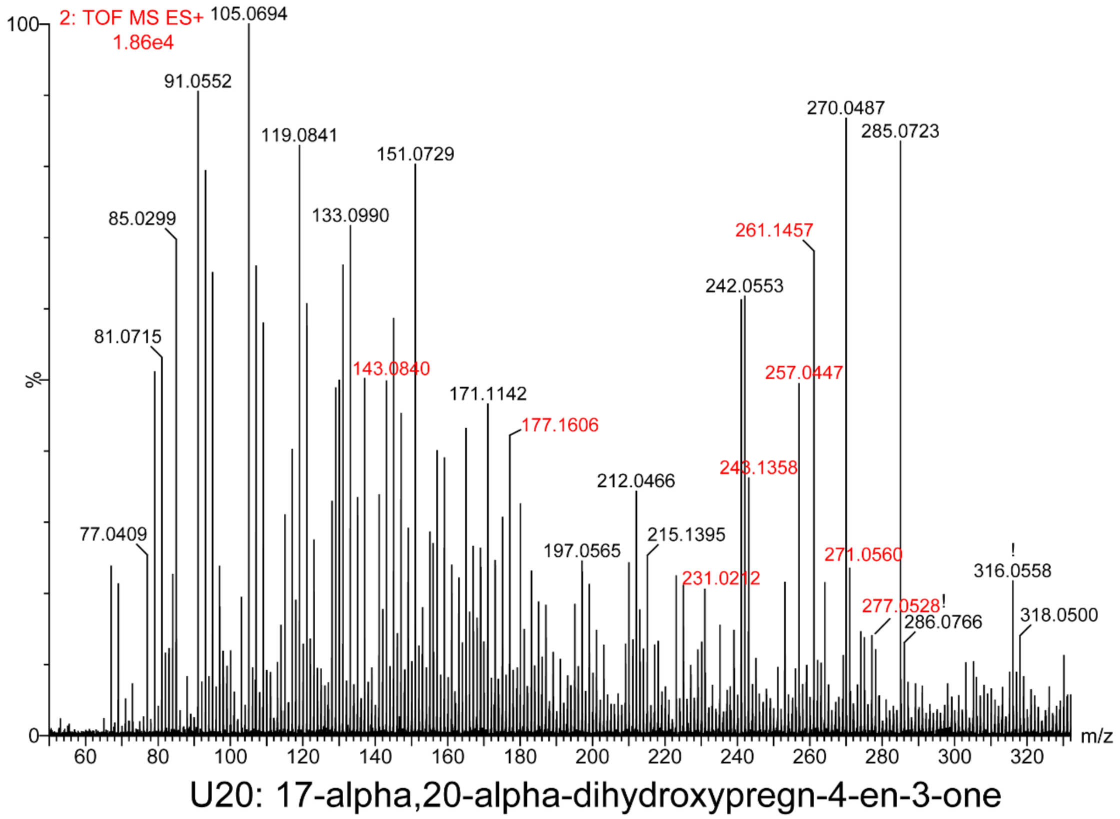

Figure S37: MS/MS spectra of the identified potential marker17-alpha,20-alpha-dihydroxypregn-4-

en-3-one in urine samples.

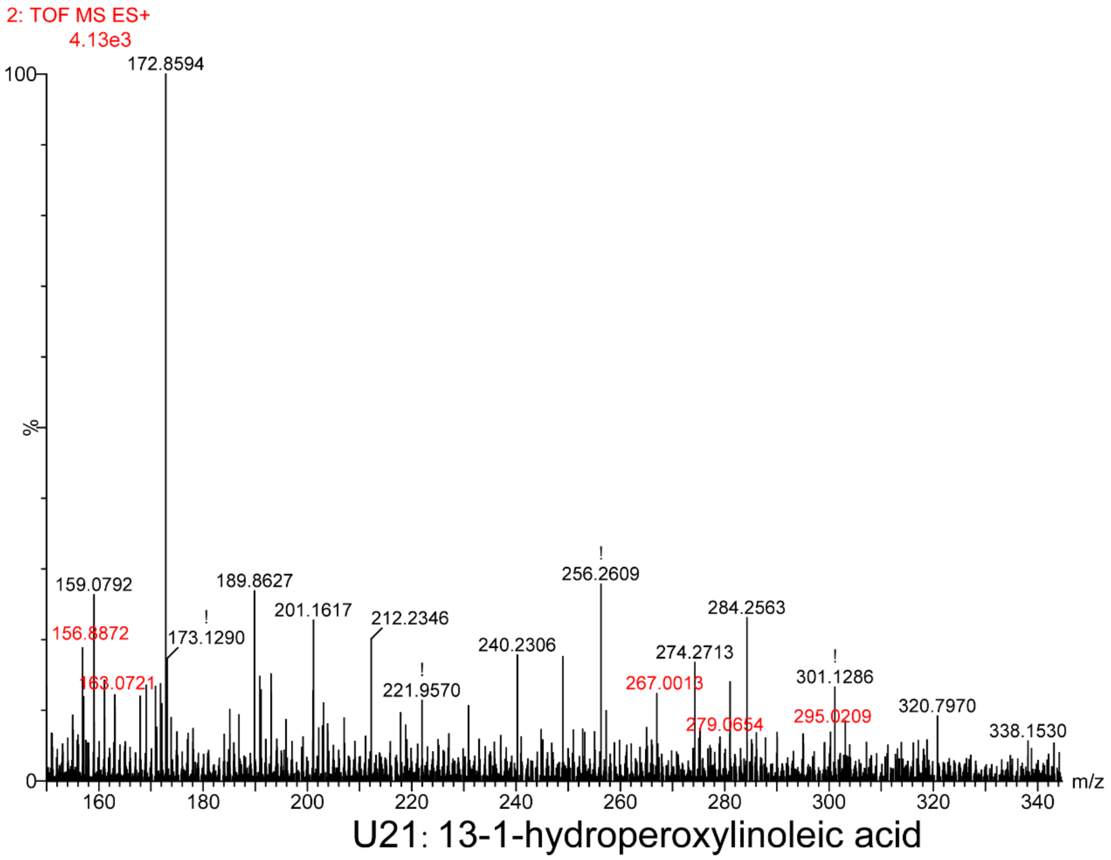

Figure S38: MS/MS spectra of the identified potential marker 13-l-hydroperoxylinoleic acid in urine samples.

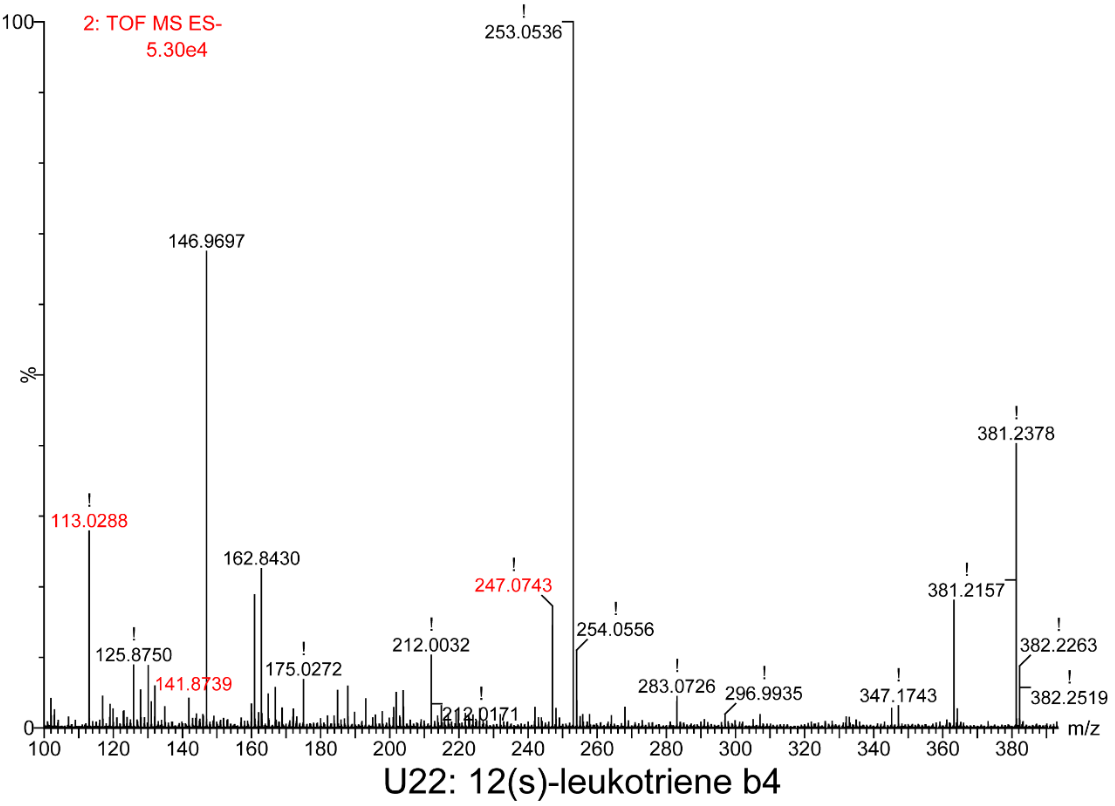

Figure S39: MS/MS spectra of the identified potential marker 12(s)-leukotriene b4 in urine samples.

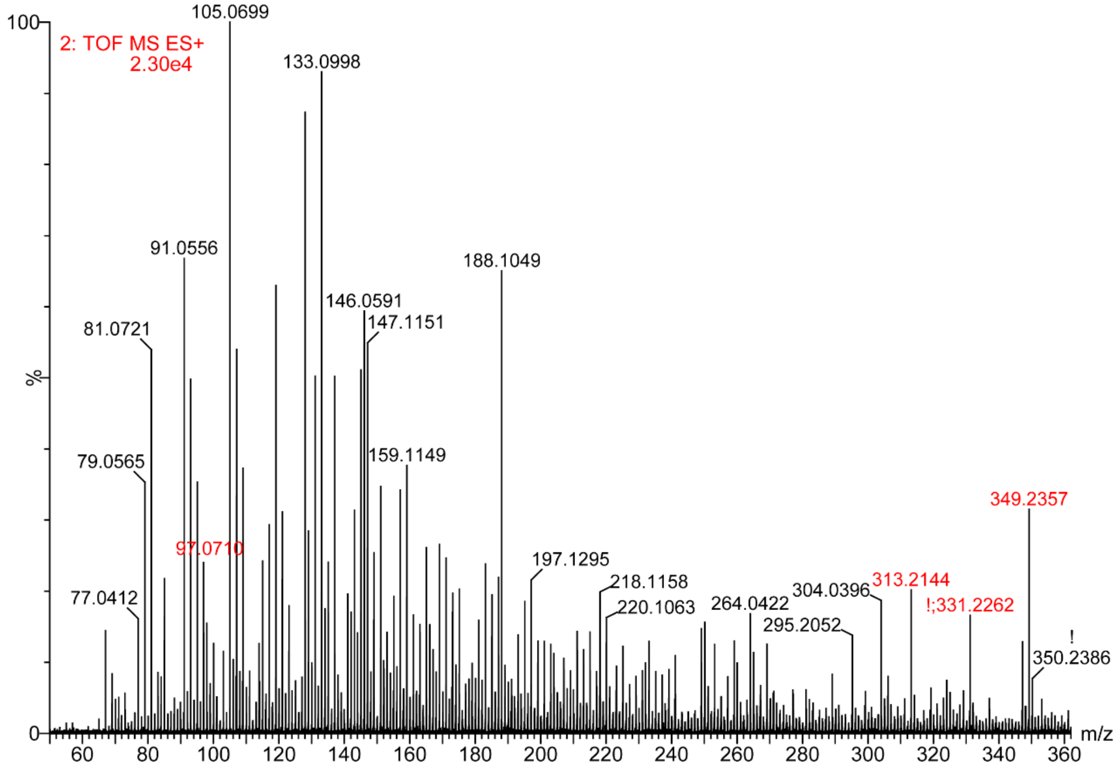

U23: 3a,21-dihydroxy-5b-pregnane-11,20-dione

Figure S40: MS/MS spectra of the identified potential marker 3a,21-dihydroxy-5b-pregnane-11,20-dione in urine samples.

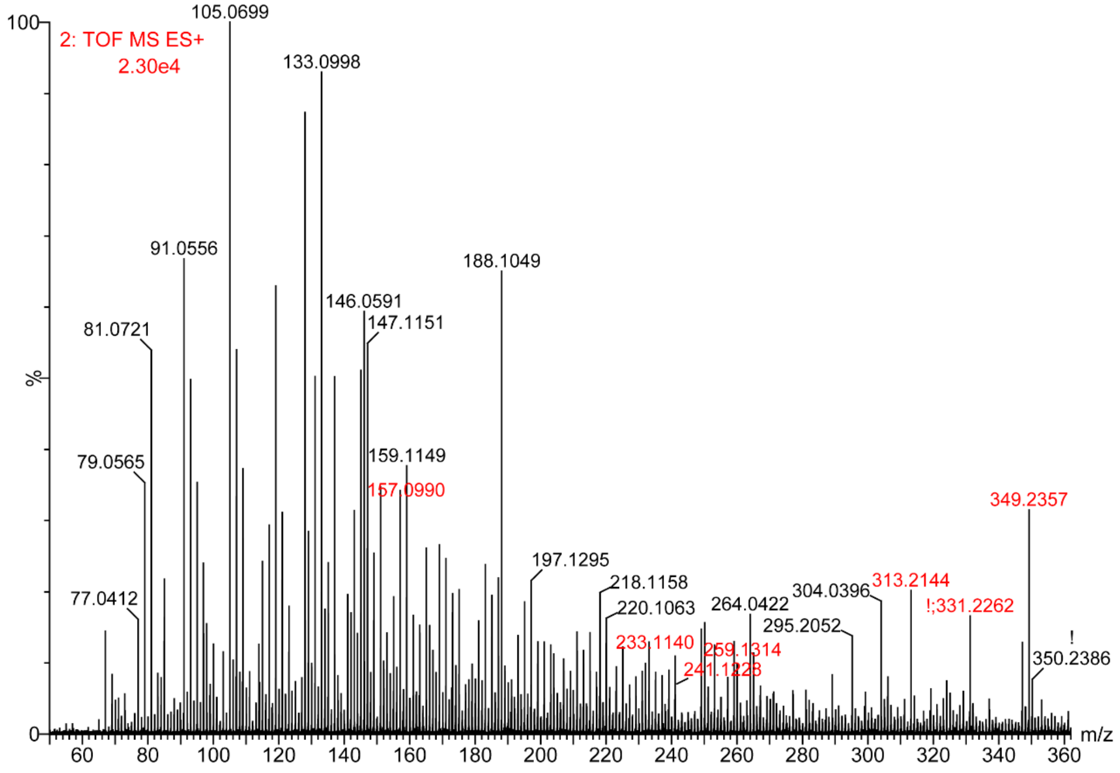

U24: 17a,21-dihydroxypreg-nenolone

Figure S41: MS/MS spectra of the identified potential marker 17a,21-dihydroxypreg-nenolone in

urine samples.

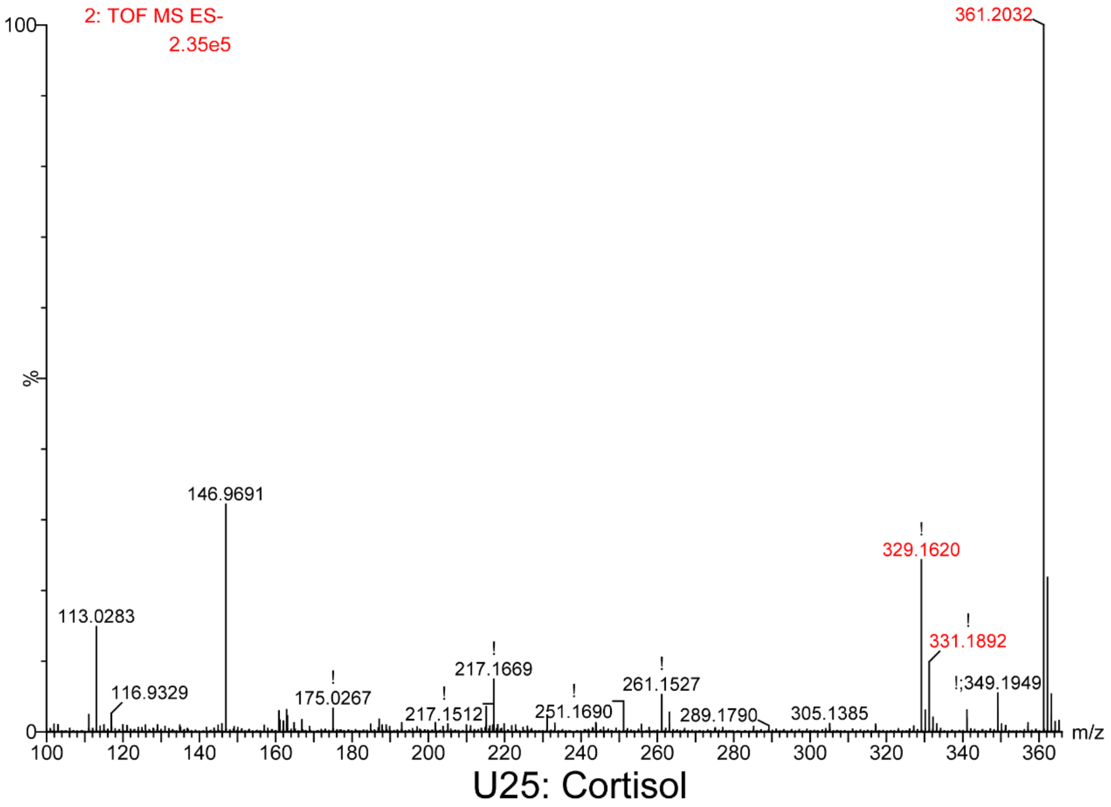

Figure S42: MS/MS spectra of the identified potential marker Cortisol in urine samples.

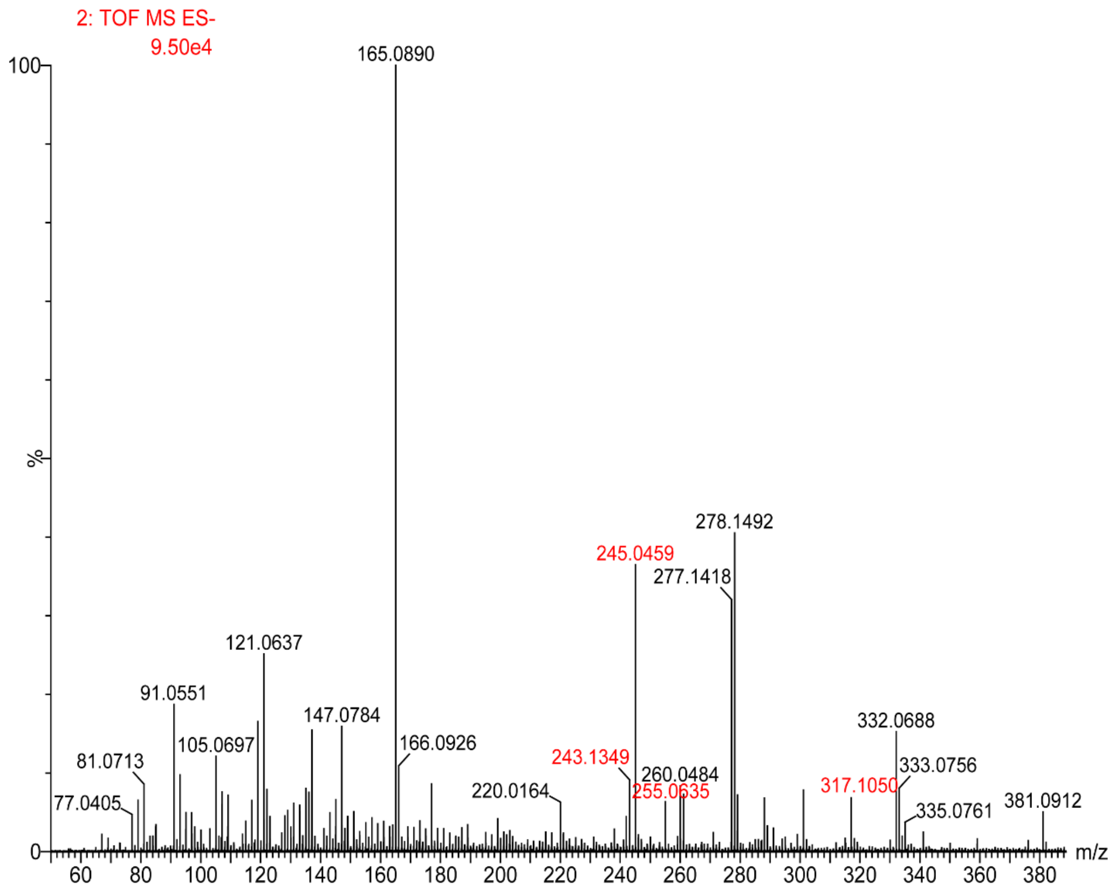

U26: Leukotriene a4

Figure S43: MS/MS spectra of the identified potential marker Leukotriene a4 in urine samples.

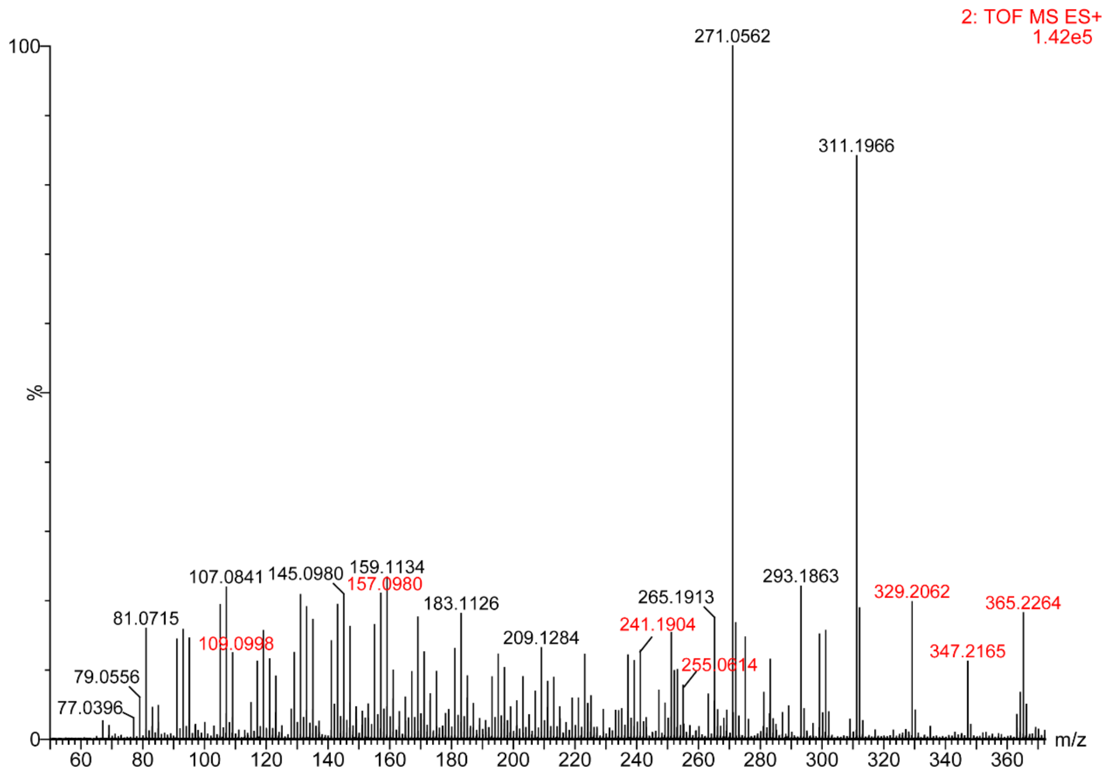

U27: 11b,17a,21-trihydroxypregnenolone

Figure S44: MS/MS spectra of the identified potential marker 11b,17a,21-trihydroxypreg-nenolone in urine samples.

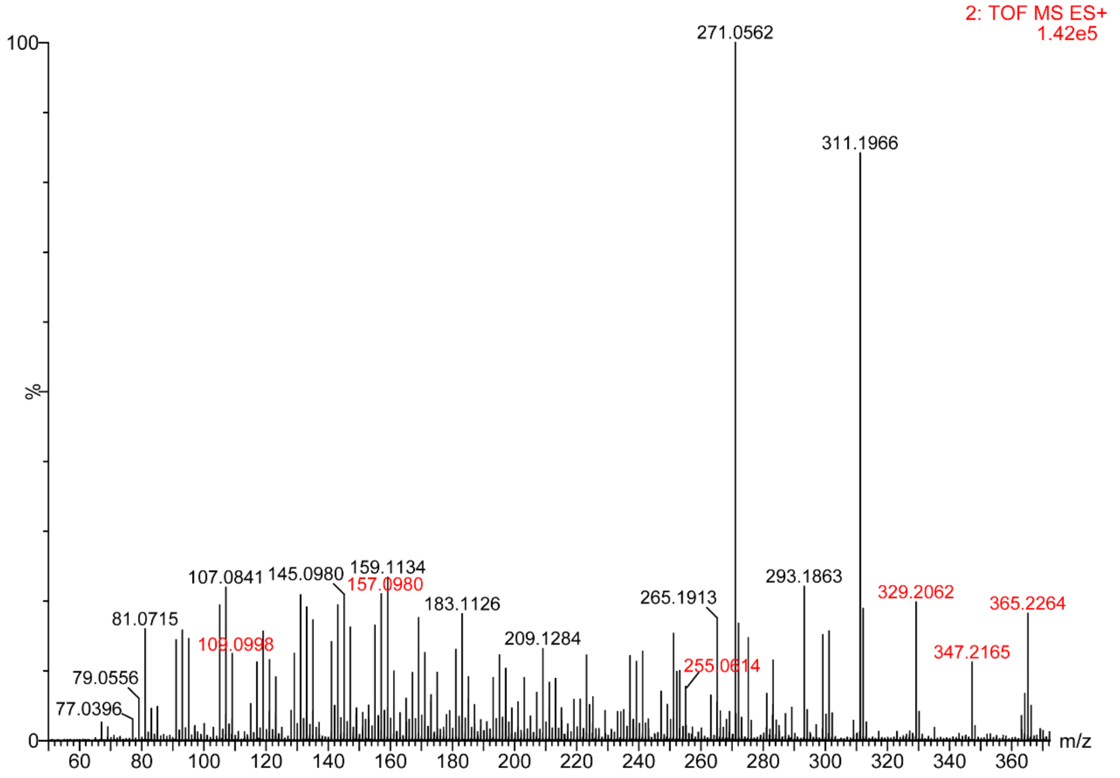

U28: Dihydrocortisol

Figure S45: MS/MS spectra of the identified potential marker Dihydrocortisol in urine samples.

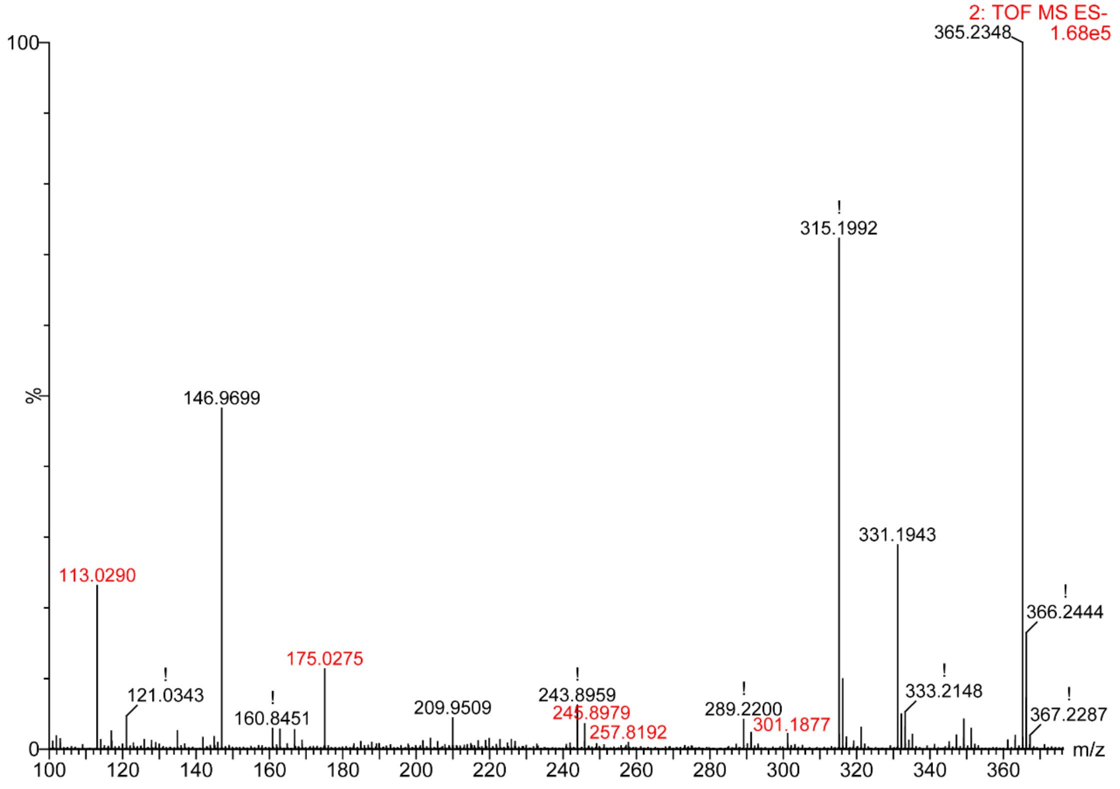

U29: 15(s)-hete

Figure S46: MS/MS spectra of the identified potential marker 15(s)-hete in urine samples.

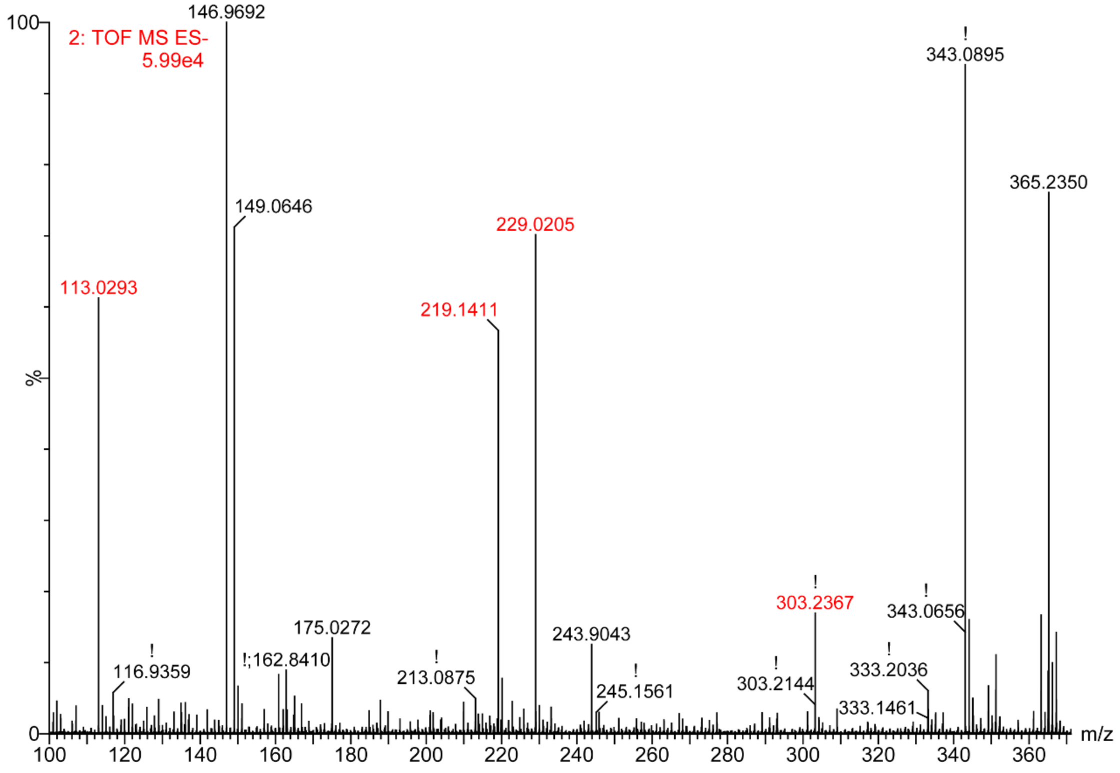

U30: 14,15-epoxy-5,8,11-eicosatrienoic acid

Figure S47: MS/MS spectra of the identified potential marker 14,15-epoxy-5,8,11-eicosatrienoic acid in urine samples.

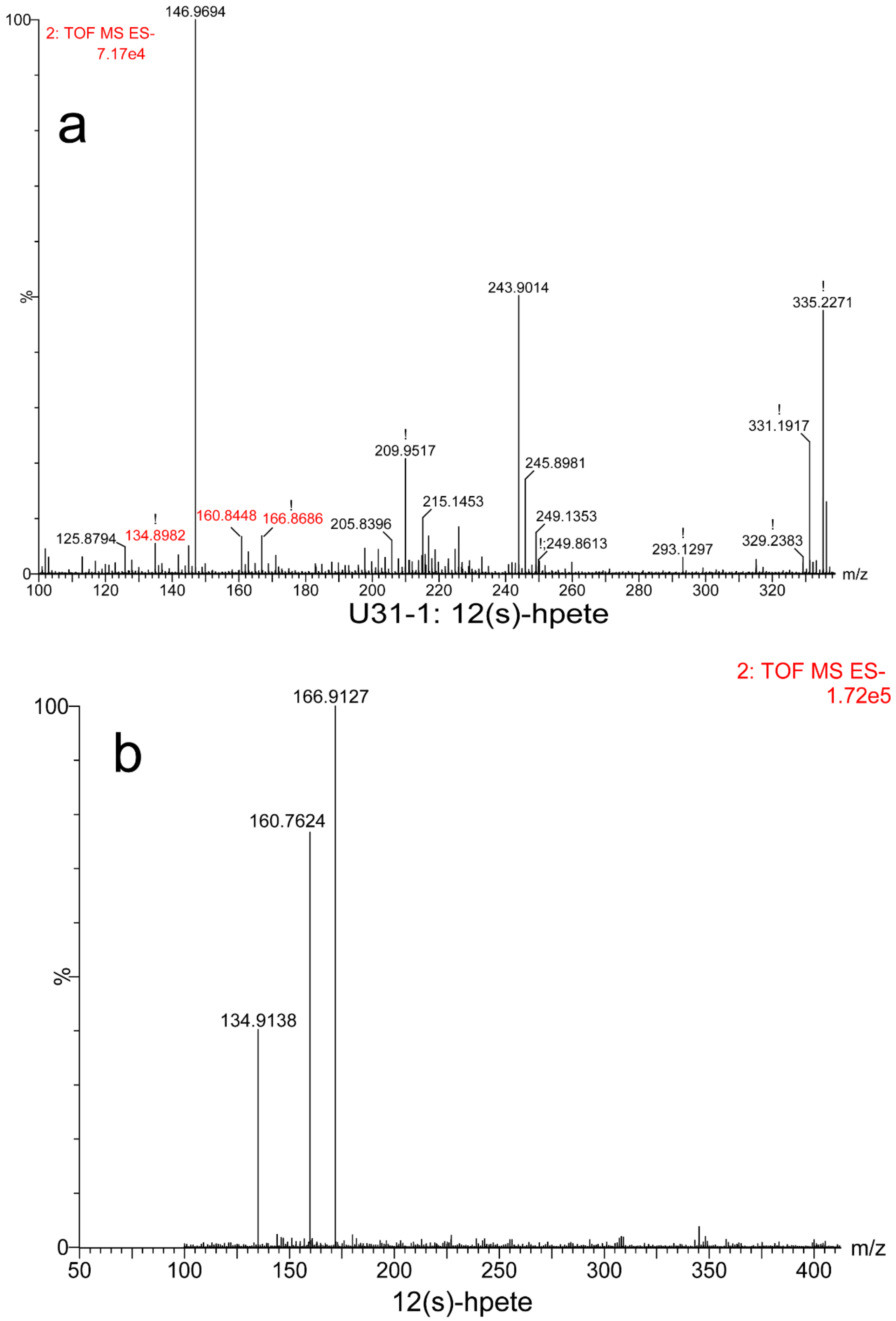

Figure S48: (a) MS/MS spectra of the identified potential marker 12(s)-hpete in urine samples. (b) The reference standard chemical spectra of 12(s)-hpete in experimental condition.

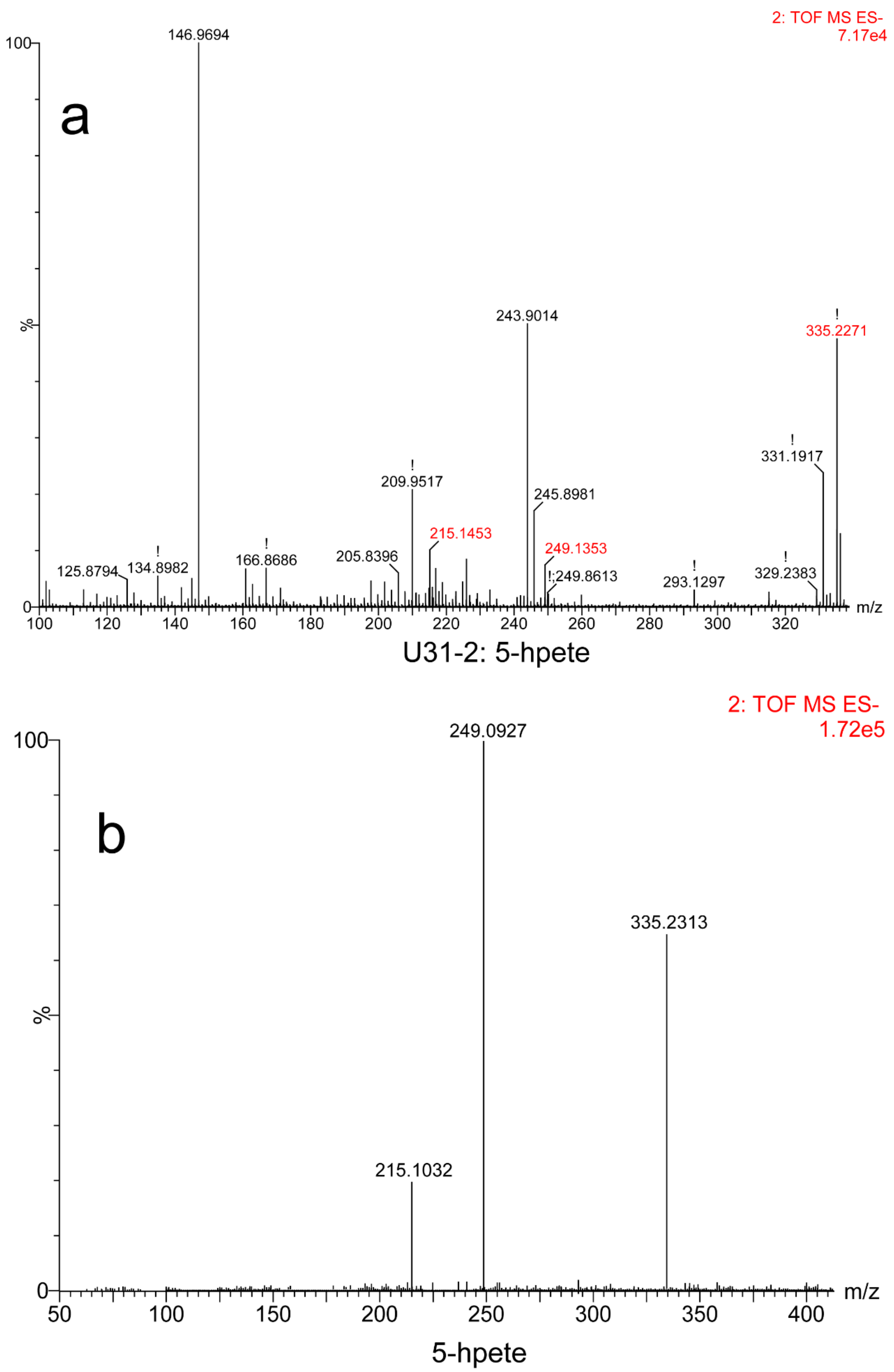

Figure S49: (a) MS/MS spectra of the identified potential marker 5-hpete in urine samples. (b) The reference standard chemical spectra of 5-hpete in experimental condition.

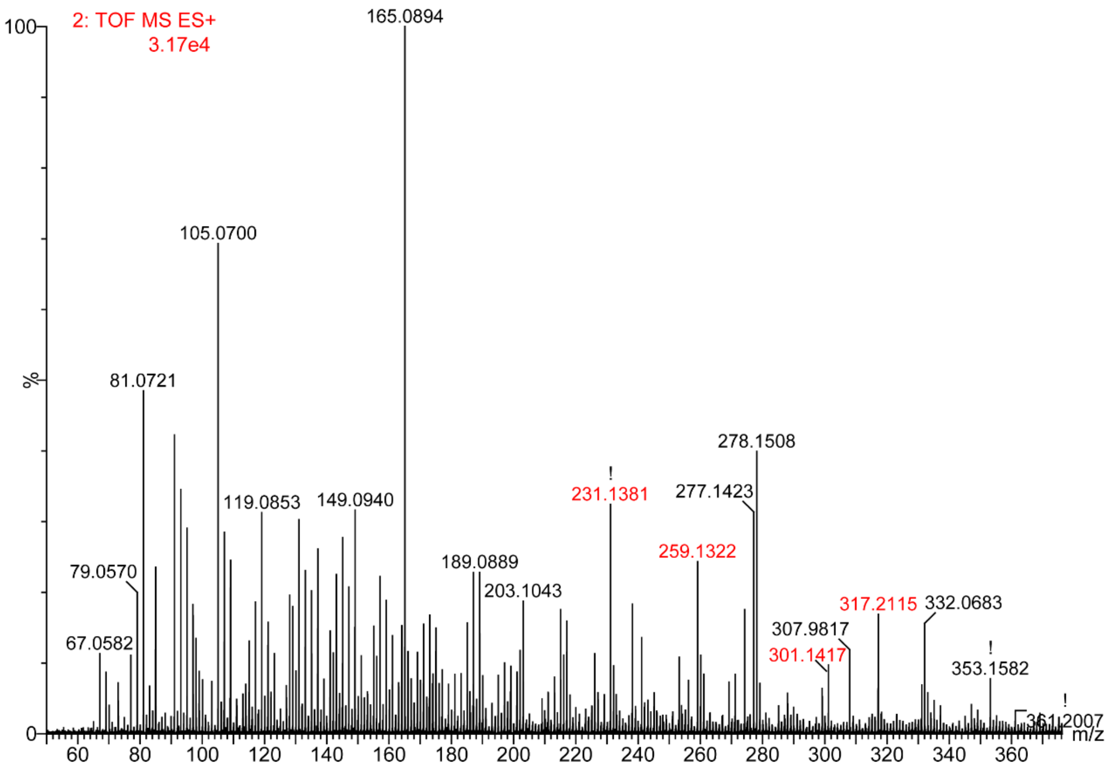

U32: Cortisone

Figure S50: MS/MS spectra of the identified potential marker Cortisone in urine samples.

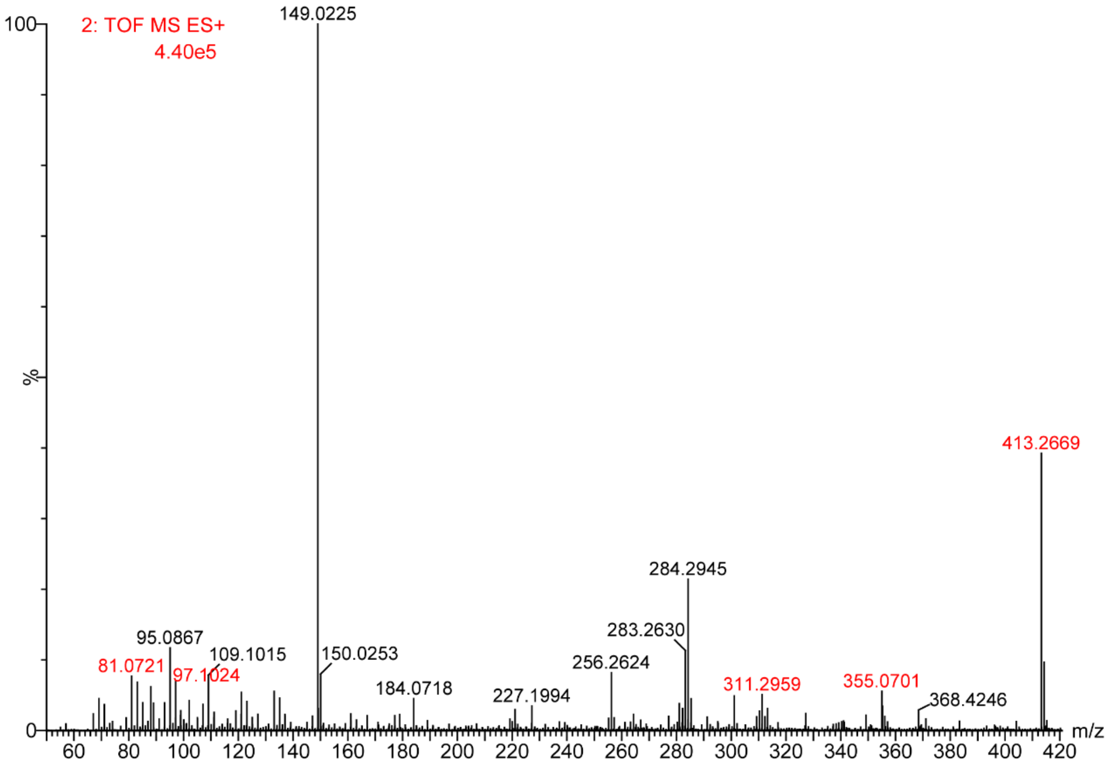

U33: 4a-methylfecosterol

Figure S51: MS/MS spectra of the identified potential marker 4a-methylfecosterol in urine samples.

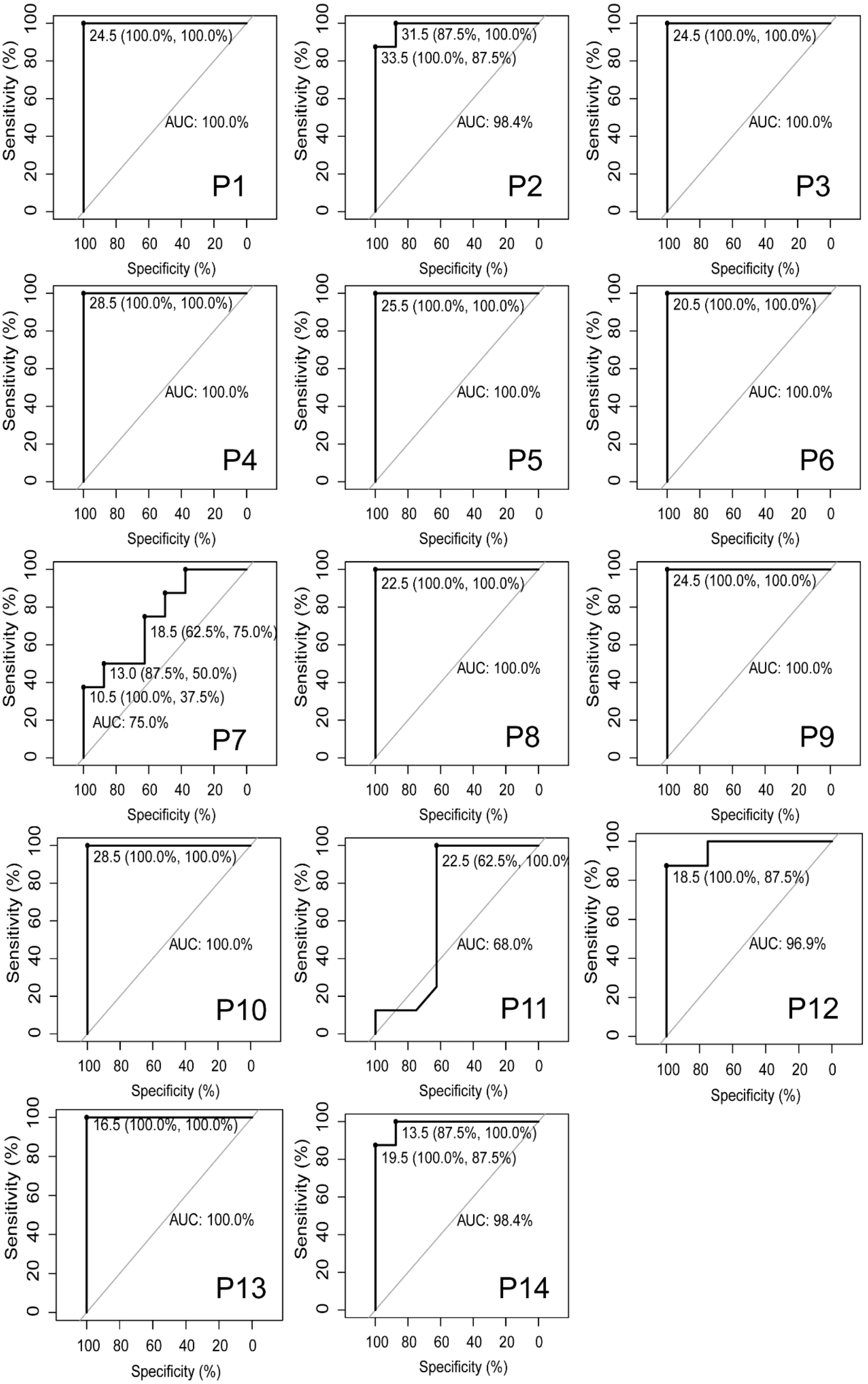
P1
24.5 (100.0%, 100.0%)
AUC: 100.0%
P2
31.5 (87.5%, 100.0%)
33.5 (100.0%, 87.5%)
AUC: 98.4%
P3
24.5 (100.0%, 100.0%)
AUC: 100.0%
P4
28.5 (100.0%, 100.0%)
AUC: 100.0%
P5
25.5 (100.0%, 100.0%)
AUC: 100.0%
P6
20.5 (100.0%, 100.0%)
AUC: 100.0%
P7
18.5 (62.5%, 75.0%)
13.0 (87.5%, 50.0%)
10.5 (100.0%, 37.5%)
AUC: 75.0%
P8
22.5 (100.0%, 100.0%)
AUC: 100.0%
P9
24.5 (100.0%, 100.0%)
AUC: 100.0%
P10
28.5 (100.0%, 100.0%)
AUC: 100.0%
P11
22.5 (62.5%, 100.0%
AUC: 68.0%
P12
18.5 (100.0%, 87.5%)
AUC: 96.9%
P13
16.5 (100.0%, 100.0%)
AUC: 100.0%
P14
13.5 (87.5%, 100.0%)
19.5 (100.0%, 87.5%)
AUC: 98.4%
Sensitivity (%)
Specificity (%)
0 20 40 60 80 100
100 80 60 40 20 0

Figure S52: Evaluation on the accuracy of all differential metabolites to be biomarkers in plasma samples. All metabolites except P7, P11 have the potential to be biomarkers with high diagnostic accuracy. AUC, Area Under Curve.

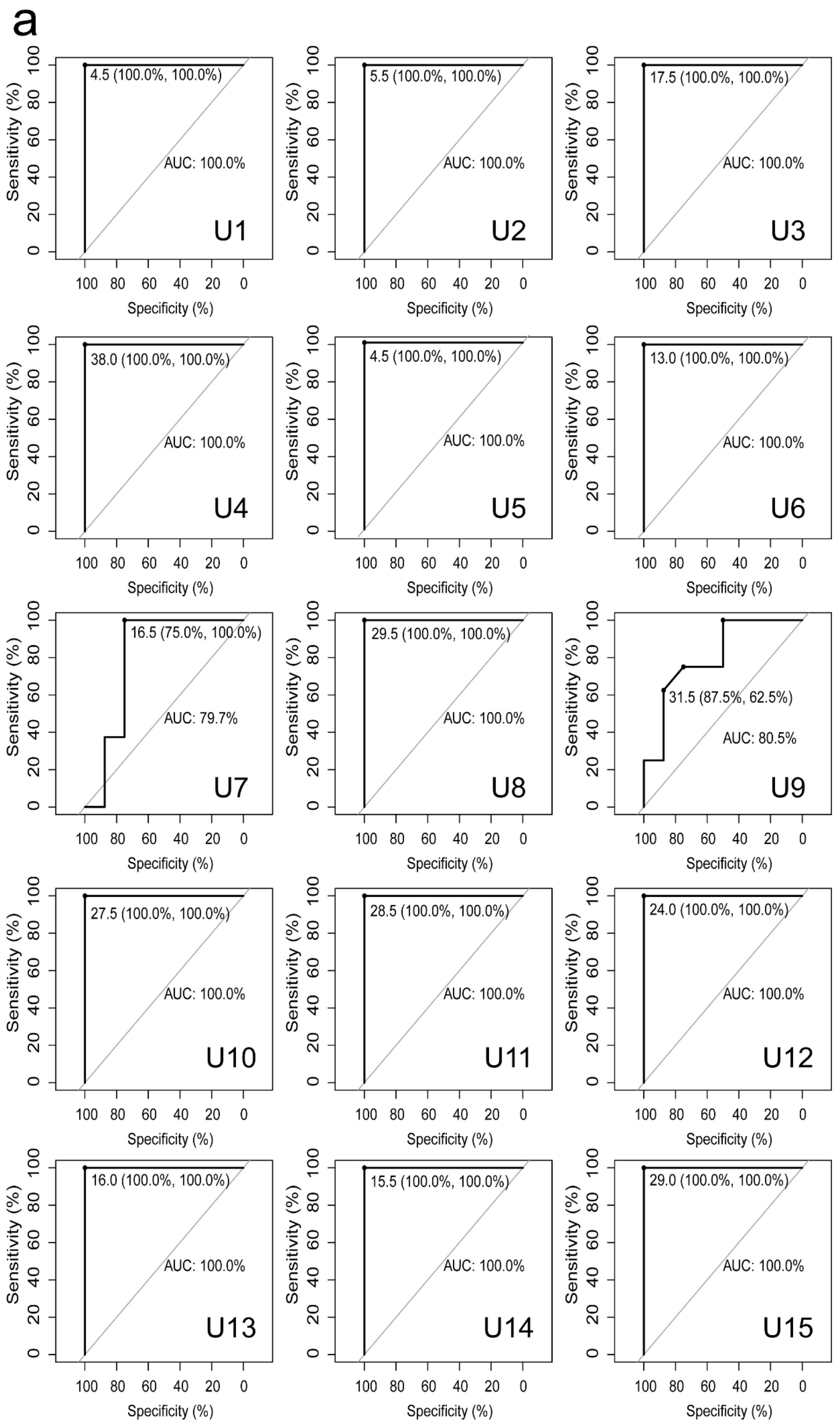
a
U1
4.5 (100.0%, 100.0%)
AUC: 100.0%
U2
5.5 (100.0%, 100.0%)
AUC: 100.0%
U3
17.5 (100.0%, 100.0%)
AUC: 100.0%
U4
38.0 (100.0%, 100.0%)
AUC: 100.0%
U5
4.5 (100.0%, 100.0%)
AUC: 100.0%
U6
13.0 (100.0%, 100.0%)
AUC: 100.0%
U7
16.5 (75.0%, 100.0%)
AUC: 79.7%
U8
29.5 (100.0%, 100.0%)
AUC: 100.0%
U9
31.5 (87.5%, 62.5%)
AUC: 80.5%
U10
27.5 (100.0%, 100.0%)
AUC: 100.0%
U11
28.5 (100.0%, 100.0%)
AUC: 100.0%
U12
24.0 (100.0%, 100.0%)
AUC: 100.0%
U13
16.0 (100.0%, 100.0%)
AUC: 100.0%
U14
15.5 (100.0%, 100.0%)
AUC: 100.0%
U15
29.0 (100.0%, 100.0%)
AUC: 100.0%
Sensitivity (%)
Specificity (%)

b

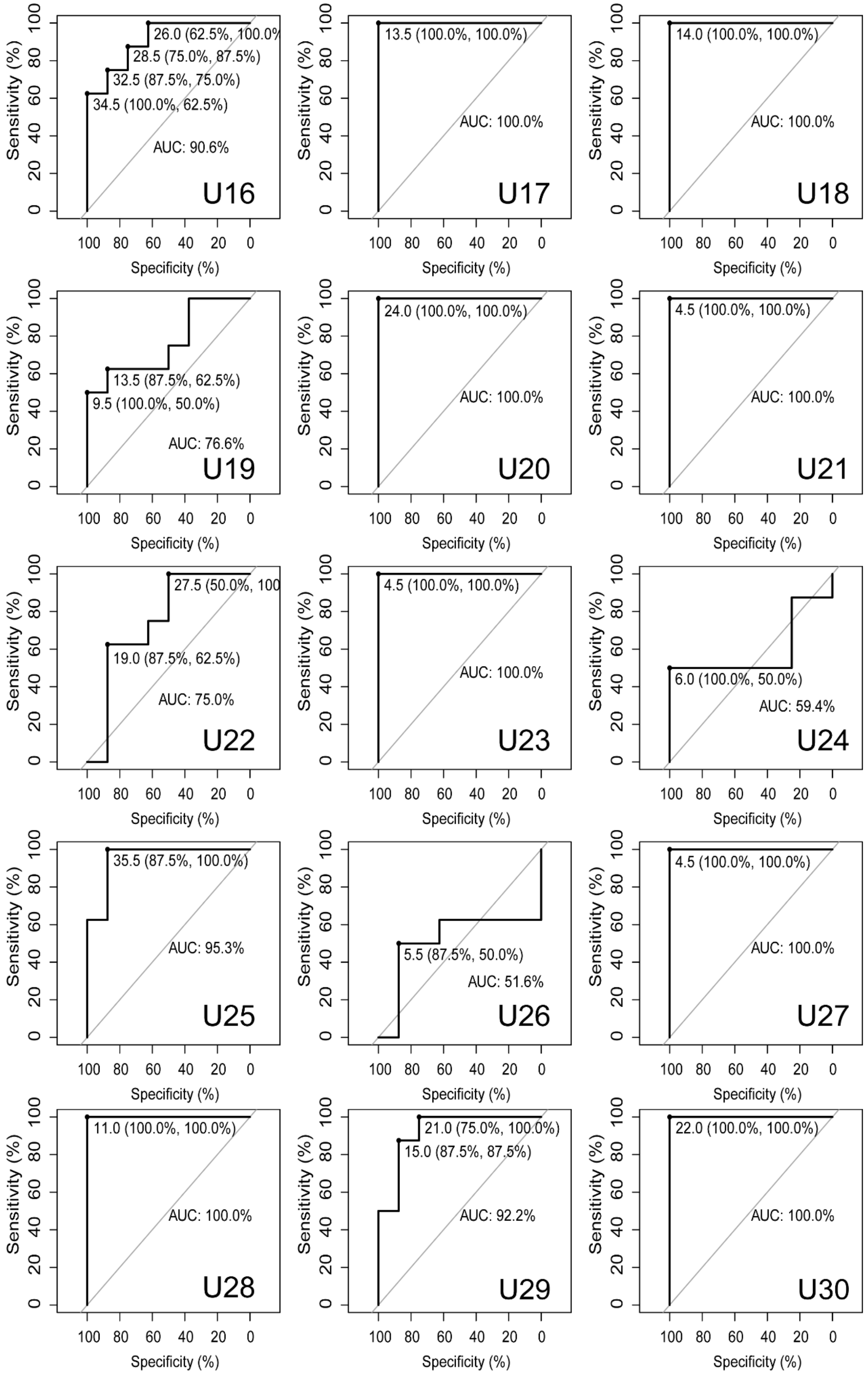

26.0 (62.5%, 100.0%
28.5 (75.0%, 87.5%)
32.5 (87.5%, 75.0%)
34.5 (100.0%, 62.5%)
AUC: 90.6%
U16
13.5 (100.0%, 100.0%)
AUC: 100.0%
U17
14.0 (100.0%, 100.0%)
AUC: 100.0%
U18
13.5 (87.5%, 62.5%)
9.5 (100.0%, 50.0%)
AUC: 76.6%
U19
24.0 (100.0%, 100.0%)
AUC: 100.0%
U20
4.5 (100.0%, 100.0%)
AUC: 100.0%
U21
27.5 (50.0%, 100
19.0 (87.5%, 62.5%)
AUC: 75.0%
U22
4.5 (100.0%, 100.0%)
AUC: 100.0%
U23
6.0 (100.0%, 50.0%)
AUC: 59.4%
U24
35.5 (87.5%, 100.0%)
AUC: 95.3%
U25
5.5 (87.5%, 50.0%)
AUC: 51.6%
U26
4.5 (100.0%, 100.0%)
AUC: 100.0%
U27
11.0 (100.0%, 100.0%)
AUC: 100.0%
U28
21.0 (75.0%, 100.0%)
15.0 (87.5%, 87.5%)
AUC: 92.2%
U29
22.0 (100.0%, 100.0%)
AUC: 100.0%
U30
Sensitivity (%)
Specificity (%)
0
20
40
60
80
100

C

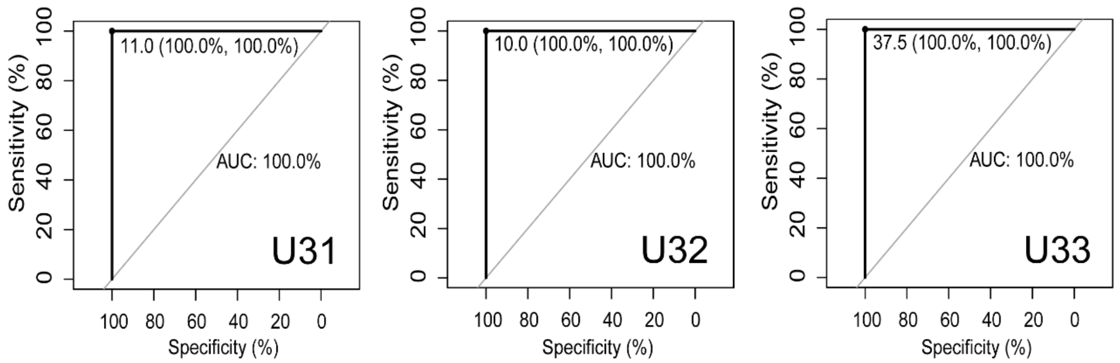

Figure S53: Evaluation on the accuracy of all differential metabolites to be biomarkers in urine samples. (a) 1-13 potential biomarkers in urine samples were evaluated with receiver operating characteristic curve (ROC). (b) 14-30 potential biomarkers in urine samples were evaluated with receiver operating characteristic curve (ROC). (c) 31-33 potential biomarkers in urine samples were evaluated with receiver operating characteristic curve (ROC). All metabolites except U7, U9, U19, U22, U24, U26, U29 have the potential to be biomarkers with high diagnostic accuracy. AUC, Area Under Curve.
